# Influenza Vaccine Effectiveness Against Symptomatic Influenza in Primary Care: A Test Negative Case Control Study Over Two Influenza Seasons 2022/2023 and 2023/2024 in Ireland

**DOI:** 10.1111/irv.70023

**Published:** 2024-12-02

**Authors:** Louise Marron, Adele McKenna, Joan O'Donnell, Michael Joyce, Charlene Bennett, Jeff Connell, Lisa Domegan

**Affiliations:** ^1^ Health Service Executive, Health Protection Surveillance Centre Dublin 1 Ireland; ^2^ ECDC Fellowship Programme, Field Epidemiology Path (EPIET), European Centre for Disease Prevention and Control (ECDC) Stockholm Sweden; ^3^ Irish College of General Practitioners Dublin 2 Ireland; ^4^ National Virus Reference Laboratory University College Dublin Dublin 4 Ireland

**Keywords:** influenza, influenza vaccine, LAIV, sentinel, surveillance, vaccine effectiveness

## Abstract

**Introduction:**

Live attenuated influenza vaccine (LAIV) is recommended in Ireland for all children aged 2–17 years. Quadrivalent influenza vaccine (QIV) is recommended for all others eligible for vaccination, including those ≥ 18 years with underlying medical conditions and all aged ≥ 65 years. We aimed to estimate influenza vaccine effectiveness (IVE) against acute respiratory infection (ARI) presentations to primary care due to influenza over two influenza seasons in Ireland, to inform vaccination recommendations and communication campaigns.

**Methods:**

We undertook a test‐negative case control study within the Irish sentinel general practice surveillance network as part of the Vaccine Effectiveness Burden and Impact Studies (VEBIS) network. We compared influenza vaccination status among influenza PCR positive cases with influenza PCR negative controls, both with ARI presentations, of all ages. We estimated IVE using logistic regression adjusting for age, onset time, medical conditions and sex.

**Results:**

In 2022/2023, there were 288 cases and 765 controls. In 2023/2024, there were 567 cases and 1832 controls. In 2022/2023, overall IVE was 42% (95% CI 9 to 64) and 50% (95% CI −30 to 83) in 2‐ to 17‐year‐olds. Overall IVE in 2023/2024 was 35% (95% CI 15 to 51) and 68% (95% CI 30 to 87) in 2‐ to 17‐year‐olds.

**Conclusion:**

Influenza vaccination reduced the risk of influenza among ARI patients presenting to general practice, demonstrating the benefits of vaccination, particularly among children. Promotion of the seasonal influenza vaccine to recommended groups, should remain a public health priority. Targeted vaccination campaigns for children promoting LAIV should emphasise the effectiveness of LAIV in children.

## 1 Introduction

In 2022/2023 and 2023/2024, the World Health Organization (WHO) vaccine strain selection committee recommended that egg‐based quadrivalent vaccines for use in the northern hemisphere contained, an A/Darwin/9/2021 (H3N2)‐like virus for influenza A(H3N2) and a B/Austria/1359417/2021 (B/Victoria lineage)‐like virus and a B/Phuket/3073/2013 (B/Yamagata lineage)‐like virus for influenza B [1, 2]. In 2022/2023, an A/Victoria/2570/2019 (H1N1)pdm09‐like virus was recommended while in 2023/2024 an A/Victoria/4897/2022 (H1N1)pdm09‐like virus was recommended as the influenza A(H1N1)pdm09 vaccine strain virus [1, 2].

In Ireland, in 2022/2023 and 2023/2024, seasonal influenza vaccination was recommended for all children aged 2–17 years, all adults ≥ 65 years and for specific groups at‐risk of severe disease from influenza infection aged 6 to 23 months and 18 to 64 years (supporting information) [3, 4]. Influenza vaccination is free of charge for all those in recommended groups. Two types/categories of influenza vaccine were licenced for use in Ireland, live attenuated influenza vaccine (LAIV) (Fluenz Tetra), recommended for all children aged 2–17 years, and non‐live quadrivalent influenza vaccine (QIV) (Influvac tetra), recommended for all others eligible for vaccination [3, 4]. Adjuvanted QIV (aQIV) was recommended by the National Immunisation Advisory Committee (NIAC) in Ireland for those aged 65 years and older. QIV was recommended if aQIV was unavailable [3, 4]. In Ireland, for the 2022/2023 and 2023/2024 seasons, aQIV was not available.

In Ireland, influenza vaccine uptake in 2022/2023 in those aged ≥ 65 years was 77% and was 15% in children aged 2–17 years [5]. In 2023/2024, uptake was 75% in those aged ≥ 65 years and was 16% in children aged 2–17 years [6]. Influenza vaccination uptake is not reported nationally by specific medical condition in at‐risk groups. However, in the 2022/2023 season among those aged 6 months to 64 years with an underlying medical risk who were vaccinated, the underlying medical conditions included chronic respiratory disease (29%), immunosuppression (25%), diabetes mellitus (15%) and chronic heart disease (14%) [5].

In 2022/2023 and 2023/2024 in Ireland, influenza A(H3N2), influenza A(H1N1)pdm09 and influenza B co‐circulated [7, 8]. In both seasons, circulation of influenza A predominated. The peak in influenza case numbers was week 51/2022 (ending 18/12/2022) during the 2022/2023 season and week 3/2024 (ending 21/01/2024) during the 2023/2024 season (Figures S1–S4) [7, 8].

We aimed to estimate influenza vaccine effectiveness (IVE) against acute respiratory infection (ARI) presentations to primary care due to influenza over two influenza seasons in Ireland, overall, by influenza (sub)type and by age group to inform vaccination recommendations and communication campaigns.

## 2 Methods

We undertook a test‐negative design (TND) case control study within the framework of the Irish sentinel general practice (GP) surveillance network to estimate IVE. This study was part of the multicentre European Centre for Disease Prevention and Control (ECDC) TND case control studies of vaccine effectiveness against symptomatic laboratory‐confirmed influenza at primary care level and part of the Vaccine Effectiveness, Burden and Impact Studies (VEBIS) of COVID‐19 and influenza network. In Ireland, this is a collaborative project involving the Health Protection Surveillance Centre (HPSC), the National Virus Reference Laboratory (NVRL), the Irish College of General Practitioners (ICGP) and the Irish sentinel GP network. Currently, in Ireland, there are 100 sentinel GP practices, with an estimated population coverage of 18%.

An ARI patient was defined as an individual who consulted a participating GP, presented with sudden onset of symptoms AND at least one of the following four respiratory symptoms: cough, sore throat, shortness of breath and coryza and a clinician's judgement that the illness was due to infection [9]. The ARI case definition has been used since 09/11/2022 to identify cases for respiratory virus swabbing in sentinel GP practices. Prior to this, the influenza‐like illness (ILI) case definition was used [9]. The study population was individuals of all ages who consulted a GP participating in the sentinel GP surveillance network and met the case definition for an ARI during the 2022/2023 or 2023/2024 influenza seasons. From 09/11/2022, sentinel GPs swabbed patients meeting the ARI case definition within 7 days of symptom onset using systematic sampling where GPs were requested to swab the first five ARI patients that consult each week. If the GP did not state that the case met either the ILI or ARI case definition, we excluded participants. Additional inclusion and exclusion criteria are in the supporting information.

In 2022/2023, due to the change in case definition, we included participants with a swab date on or after 09/11/2022. The study period in 2022/2023 ended in ISO week 18 (ending 07/05/2024), the week that the last laboratory‐confirmed influenza case was reported. In 2023/2024, the study period began in ISO week 42 2023 (beginning 16/10/2024), 14 days after the beginning of the influenza vaccination campaign and ended in ISO week 20 2024 (ending 19/05/2024), the week the last influenza case was reported.

We defined a case as a person of any age who presented to a GP and met the ARI case definition (as reported by the GP) with a respiratory sample polymerase chain reaction (PCR) positive for influenza virus. We defined a test‐negative control as a person of any age who presented to a GP and met the ARI case definition (as reported by the GP) with a respiratory specimen PCR negative for all influenza viruses.

We considered an individual vaccinated if they received one dose of the seasonal influenza vaccine ≥ 14 days before symptom onset. Individuals were considered unvaccinated if they did not receive influenza vaccine in the season or were vaccinated on/after symptom onset date. We excluded participants if they had no influenza vaccination date documented and/or were vaccinated 1–13 days before symptom onset. Influenza vaccination status and date was reported by sentinel GPs and was validated at the HPSC. If vaccination status was recorded as unknown or if vaccination date was missing, we verified vaccination status on Ireland's national vaccination database for COVID‐19 and influenza (COVAX).

For each participant, a nasal/throat swab specimen was collected. Specimens were tested at the NVRL using PCR for influenza A(H3), A(H1)pdm09 and B viruses, SARS‐CoV‐2, respiratory syncytial virus (RSV), adenovirus, parainfluenza virus types 1–4 (PIV‐1‐4), human metapneumovirus (hMPV), rhino/enteroviruses and bocavirus.

We calculated sample size using methodology detailed in the ECDC protocol for IVE studies [9], using a dynamic version of a sample size calculator in MS Excel. Assuming a case to control ratio of 1:1 for an estimated vaccine coverage of 30% and a lower confidence limit of the IVE estimate of 30%, we calculated that a sample size of 255 cases and 255 controls was required for detection of an IVE of 40% (OR = 0.6).

GPs entered data (supporting information) onto the national GP online referral system (Healthlink), used for referring swabs for testing/other diagnostic referrals. The data sources for this study were the NVRL (for PCR results), Healthlink (for clinical/epidemiological data) and COVAX (for vaccination status data). A GP code and a unique patient identification number were used to link NVRL results to Healthlink data, and a unique patient identification number was used to link vaccination records on COVAX to Healthlink data.

We described cases and controls by baseline characteristics including sex, age, influenza vaccination status, presence of at least one chronic medical condition and time of symptom onset. For categorical variables, we calculated frequencies and percentages. We compared cases and controls using the Mann–Whitney *U* test for continuous variables and the chi‐square test or Fisher's exact test for categorical variables. Key variables known to be associated with IVE were identified a priori; age, sex, presence of a chronic medical condition and time of symptom onset [10]. Following initial descriptive analysis, we excluded records with missing values for key variables to create a complete case dataset for the univariable and multivariable analysis.

We calculated crude odds ratios and adjusted odds ratios (aORs) overall, by age and for influenza (sub)type and age‐specific (sub)type using univariable and multivariable logistic regression. IVE was computed as IVE = (1 − aOR)*100. A 95% confidence interval was calculated around the point estimate. All models were adjusted for age, time of symptom onset, sex and presence of a chronic condition. To identify best model fit, we varied the functional forms of the age and time of symptom onset variables which we modelled as continuous, categorical (with various numbers of categories) and as restricted cubic splines with 3 or 4 knots. We compared the Akaike information criterion (AIC) for each full model to determine which functional form resulted in the best model fit. We used the following criteria when conducting multivariable analysis to determine whether a valid measure of IVE could be calculated: (i) there were 10 cases or controls (whichever was smaller) for each parameter in the model (10 events per variable), (ii) there were at least 10–15 cases or controls (whichever was smaller) and (iii) precision of the IVE estimate did not span −200 and 90%. If any criteria were violated, we undertook a sensitivity analysis using Firth's penalised regression. If the IVE estimate from the penalised logistic regression and the standard logistic regression largely differed (by ≥ 10%) or if the precision spanned −200 and 90%, we did not report the estimate. As a correlation between influenza and COVID‐19 vaccination may bias IVE estimates [11], we conducted a sensitivity analysis calculating the overall IVE for all ages for each season excluding SARS‐CoV‐2 positive controls. All data analysis was conducted in R version 4.3.1 [12].

## 3 Results

In 2022/2023, 1094 participants were included in the descriptive analysis; 296 cases (27%) and 798 controls (73%). In the complete case dataset, 1053 (60%) participants were included, 288 cases (27%) and 765 controls (73%). In 2023/2024, 2641 participants were included in the descriptive analysis; 626 cases (27%) and 2015 controls (73%). In the complete case dataset, 2399 (76%) participants were included; 567 cases (24%) and 1832 controls (76%). Details of restrictions and exclusions are reported in the supporting information.

The median age of cases and controls was 33 years (IQR 15–47) and 35 years (IQR (11–55) in 2022/23 (Table 1) and was 35 years (19–49) and 39 years (IQR 19–57) in 2023/2024 (Table 2). In both seasons, overall vaccination coverage was 23% (246/1094 in 2022/2023 and 624/2641 in 2023/2024). In 2022/2023, 25.8% of controls were vaccinated compared with 14.2% of cases (*p*‐value < 0.001) (Table 1). In 2023/2024, 26.1% of controls were vaccinated compared with 16.9% of cases (*p*‐value < 0.001) (Table 2). In 2022/2023 among all cases (*n* = 296), 34% were positive for influenza A (H1N1)pdm09 and 34% were positive for influenza A (H3N2) infection. Influenza B cases accounted for 28% of infections (Table 1). In 2023/2024 among all cases (*n* = 626), 53.4% were positive for influenza A(H3N2), 25.1% were positive for influenza A(H1N1)pdm09 and 15.5% were positive for influenza B (Table 2). The distribution of ARI infection among cases and controls and a descriptive analysis of cases and controls (by age‐group, influenza (sub)type and age‐specific influenza (sub)type) for both seasons are included in Figures S5–S12 and Tables S1–S29.

**TABLE 1:** Descriptive characteristics of cases and controls, primary care influenza vaccine effectiveness study, Ireland 2022/2023 influenza season.

Exposure		Cases		Controls		
		(*n* = 296)		(*n* = 798)		
Continuous variables		Median	IQR	Median	IQR	*p*‐value^a^
	Age (years)	33	15–47	35	11–55	0.852
	Time from symptom onset to swab (days)	3	2–4	3	2–4	**0.005**
Categorical variables		*n*	%	*n*	%	*p*‐value^b^
Sex	Female	160	54.1	479	60.1	0.083
	Male	136	45.9	318	39.9	
	Missing	0	—	1	—	
Age group (years)	< 2	3	1	42	5.3	**< 0.001**
	2–17	83	28.1	219	27.4	
	18–64	183	61.8	412	51.6	
	65+	27	9.1	125	15.7	
Vaccination status	Unvaccinated	253	85.8	587	74.2	**< 0.001**
	Vaccinated	42	14.2	204	25.8	
	Missing^c^	1	—	7	—	
Influenza vaccine type	Quadrivalent influenza vaccine (QIV)	36	12.2	167	21.1	**0.001**
	Live attenuated influenza vaccine (LAIV)	5	1.7	26	3.3	
	Other	1	0.3	11	1.4	
	Unvaccinated	253	85.8	587	74.2	
	Missing^§^	1	—	7	—	
Chronic condition	Chronic condition	61	21.1	228	29.5	**0.008**
	No chronic condition	228	78.9	545	70.5	
	Missing	7	—	25	—	
Month of symptom onset	November to December 2022	169	57.1	273	34.2	**< 0.001**
	January to February 2023	94	31.8	281	35.2	
	March to May 2023	33	11.1	244	30.6	

		Cases (*n* = 296)		Controls (*n* = 798)		
Exposure		*n*	%	*n*	%	*p*‐value^b^
Time from symptom onset to swab (days)	0	5	1.7	15	1.9	0.092
	1	52	17.6	105	13.2	
	2	77	26.0	164	20.6	
	3	68	23.0	206	25.8	
	4	42	14.2	137	17.2	
	5	28	9.5	68	8.5	
	6	8	2.7	36	4.5	
	7	16	5.4	67	8.4	
Pregnant	No	287	98.0	781	99.1	0.215
	Yes	6	2.0	7	0.9	
	Missing	3	—	10	—	
Influenza subtype	Control	—	—	798	100.0	—
	Influenza A(H1N1)pdm09	100	33.8	—	—	
	Influenza A(H3N2)	100	33.8	—	—	
	Influenza B/lineage unknown	83	28.0	—	—	
	Coinfection Influenza A (H3N2)/Influenza B	8	2.7	—	—	
	Coinfection Influenza A (H3N2)/Influenza A(H1N1)pdm09	2	0.7	—	—	
	Coinfection Influenza A (H3N2)/influenza A(H1N1)pdm09/Influenza B	2	0.7	—	—	
	Influenza A ‐ not subtyped	1	0.3	—	—	

^a^ Mann–Whitney *U* test.

^b^ Chi‐squared test or Fisher's exact test when cell counts < 5.

^c^ Missing as vaccinated 1–13 days before onset of symptoms.

**TABLE 2:** Descriptive characteristics of cases and controls, primary care influenza vaccine effectiveness study, Ireland 2023/2024 influenza season.

Exposure		Cases		Controls		
		(*n* = 626)		(*n* = 2015)		
Continuous variables		Median	IQR	Median	IQR	*p*‐value^a^
	Age (years)	35	19–49	39	19–57	**< 0.001**
	Time from symptom onset to swab (days)	3	2–4	3	2–5	**< 0.001**
Categorical variables		*n*	%	*n*	%	*p*‐value^b^
Sex	Female	363	58.1	1256	62.3	0.063
	Male	262	41.9	759	37.7	
	Missing	1	—	0	—	
Age group (years)	< 2	13	2.1	79	3.9	**< 0.001**
	2–17	129	20.6	398	19.8	
	18–64	435	69.5	1221	60.6	
	65+	49	7.8	317	15.7	
Vaccination status	Unvaccinated	516	83.1	1466	73.9	**< 0.001**
	Vaccinated	105	16.9	519	26.1	
	Missing^c^	5	—	30	—	
Influenza vaccine type	Quadrivalent influenza vaccine	95	15.3	453	22.8	
	Live attenuated influenza vaccine	10	1.6	65	3.3	**0.001**
	Other vaccination type (not specified)	0	0	1.0	0	
	Unvaccinated	516	83.1	1466	73.9	
	Missing^c^	5	—	30	—	
Chronic condition	No chronic condition	443	77.3	1288	69.2	**< 0.001**
	Chronic condition	130	22.7	573	30.8	
	Missing	53	—	154	—	
Month of symptom onset	October to December 2023	157	25.1	749	37.2	**< 0.001**
	January to February 2024	393	62.8	758	37.6	
	March to May 2024	76	12.1	508	25.2	

		Cases (*n* = 626)		Controls (*n* = 2015)		
Exposure		*n*	%	*n*	%	*p*‐value^b^
Symptom onset to swab (days)	0	10	1.6	41	2.0	**< 0.001**
	1	79	12.6	243	12.1	
	2	193	30.8	431	21.4	
	3	145	23.2	440	21.8	
	4	91	14.5	318	15.8	
	5	48	7.7	178	8.8	
	6	31	5.0	120	6.0	
	7	29	4.6	244	12.1	
Pregnant	No	593	98.3	1940	98.4	1.000
	Yes	10	1.7	31	1.6	
	Missing	23	—	44	—	
Influenza subtype	Control	—	—	2015	100	—
	Influenza A ‐ not subtyped	37	5.9	—	—	
	Influenza A(H1N1)pdm09	157	25.1	—	—	
	Influenza A(H3N2)	334	53.4	—	—	
	Influenza B	97	15.5	—	—	
	Coinfection influenza A (H3)/influenza B	1	0.2	—	—	

^a^ Mann–Whitney *U* test.

^b^ Chi‐squared test or Fisher's exact test when cell counts < 5.

^c^ Missing as vaccinated 1–13 days before onset of symptoms.

IVE against any influenza for all ages was 42% (95% CI 9 to 64) in 2022/2023 and 35% (95% CI 15 to 51) in 2023/2024 (Tables 3 and 4). In 2‐ to 17‐year‐olds, IVE against any influenza was 50% (95% CI −30 to 83) in 2022/2023 and 68% (95% CI 30 to 87) in 2023/2024. In those aged 18–64 years, IVE was 50% (95% CI 9 to 73) in 2022/2023 and 35% (95% CI 9 to 54) in 2023/2024. In those aged ≥ 65 years, IVE was 15% (95% CI −142 to 69) in 2022/2023 and 16% (95% CI −83 to 60) in 2023/2024.

**TABLE 3:** Influenza vaccine effectiveness (IVE) estimates by age group and influenza subtype, primary care IVE study, Ireland 2022/2023.

Influenza type	Age category	Total	Cases			Controls					
			Total	Vaccinated		Total	Vaccinated		IVE^a^	CI lower	CI upper
		*n*	*n*	*n*	%	*n*	*n*	%	%	%	%
All influenza	All ages	1053	288	42	14.6	765	198	25.9	42	9	64
	2–17^b^	293	82	5	6.1	211	23	10.9	50	−30	83
	18–64	568	176	19	10.8	392	80	20.9	50	9	73
	65+^b^	148	27	18	66.7	121	95	78.5	15	−142	69
Influenza A(H1N1)pdm09	All ages	699	102	21	20.6	597	156	26.1	17	−57	58
	18–64	383	75	13	17.5	308	66	21.4	24	−63	66
Influenza A(H3N2)	All ages	582	110	17	15.5	472	119	25.2	55	8	79
	2–17^b^	166	36	3	8.3	130	13	10.0	16	−182	80
	18–64^b^	300	57	4	7.0	243	51	21.0	60	−10	88
	65+^b^	91	16	10	62.5	75	55	73.3	52	−83	81
Influenza B	All ages^b^	849	85	4	4.7	764	198	25.9	68	18	91
	2–17^b^	245	35	1	2.9	210	23	11.0	74	−14	97
	18–64^b^	441	49	2	4.1	392	80	20.4	71	6	94

*Note:* No IVE estimates reported for influenza A(H1N1)pdm09 in children aged 2–17 years or adults aged 65 years and older as conditions for reporting were violated. No IVE estimates reported for influenza B in adults aged 65 years and older as conditions for reporting were violated.

^a^ Adjusted for age, time of symptom onset, medical condition and sex.

^b^ Penalised logistic regression using Firth's method.

**TABLE 4:** Crude and adjusted influenza vaccine effectiveness (IVE) estimates by age group and influenza subtype, primary care IVE study, Ireland 2023/2024.

Influenza type	Age category	Total	Cases			Controls					
			Total	Vaccinated		Total	Vaccinated		IVE^a^	CI lower	CI upper
		*n*	*n*	*n*	%	*n*	*n*	%	%	%	%
All influenza	All ages	2399	567	97	17.1	1832	488	26.6	35	15	51
	2–17	502	124	8	6.5	378	52	13.8	68	30	87
	18–64	1458	368	58	15.0	1090	241	22.1	35	9	54
	65+^b^	331	44	31	70.5	287	195	67.9	16	−83	60
Influenza A(H1N1)pdm09	All ages	2004	142	28	19.7	1826	486	26.6	30	−13	58
	2–17^b^	182	23	2	8.7	377	51	13.5	51	−66	91
	18–64	1188	102	16	15.7	1086	241	22.2	39	−8	67
Influenza A(H3N2)	All ages	2136	307	61	19.9	1829	487	26.6	25	−6	47
	2–17	544	77	6	7.8	378	52	13.8	65	15	87
	18–64	300	194	36	18.6	1087	240	22.1	16	−29	46
	65+^b^	91	29	19	65.5	287	195	67.9	25	−80	67
Influenza B	All ages^b^	1546	85	2	2.4	1832	488	26.6	84	53	97
	2–17^b^	245	21	0	0	378	52	13.8	82	−39	100
	18–64^b^	1154	64	2	3.1	1090	241	22.1	80	38	96

*Note:* No IVE estimate is reported for influenza A(H1N1)pdm09 or influenza B in adults aged 65 years and older as the conditions for reporting were violated.

^a^ Adjusted for age, time of symptom onset, medical condition and sex.

^b^ Penalised logistic regression using Firth's method.

For the 2022/2023 and 2023/2024 seasons, respectively, IVE for all ages was 68% (95% CI 18 to 91) and 84% (95% CI 53 to 97) against influenza B, 55% (95% CI 8 to 79) and 25% (95% CI −6 to 47) against influenza A(H3N2), and 17% (95% CI −57 to 58) and 30% (95% CI −13 to 58) against influenza A(H1N1)pdm09 (Tables 3 and 4). Additional figures showing age‐specific and (sub)type specific IVE estimates are included in as Figures S13 and S14.

In the sensitivity analysis, in 2022/2023, 68/765 (8.9%) controls were positive for SARS‐CoV‐2 and in 2023/2024, 131/1832 (7.2%) controls were positive for SARS‐CoV‐2 and were excluded. In this analysis, the overall IVE point estimate was unchanged in 2022/2023 (IVE 42%, 95% CI 7 to 64) and in 2023/2024 there was a 2% difference in IVE (IVE 37%, 95% CI 16 to 53) (Table S30).

## 4 Discussion

IVE against any influenza for all ages was 42% (95% CI 9 to 64) in 2022/2023 and 35% (95% CI 15 to 51) in 2023/2024, suggesting that vaccination reduced the risk of influenza among ARI patients presenting to primary care during two influenza seasons in Ireland. Compared with the overall IVE point estimates, the IVE point estimate in children aged 2–17‐years was higher in both influenza seasons, 50% (95% CI −30 to 83) in 2022/2023 and 68% (95% CI 30 to 87) in 2023/2024. This finding suggests that children may have been better protected by influenza vaccination in both seasons compared with the overall population. Compared with the overall IVE point estimates, the IVE point estimates were lower in those aged ≥ 65 years in both seasons, 15% (95% CI −142 to 69) in 2022/2023 and 16% (95% CI −83 to 60) in 2023/2024, suggesting that older adults were less well protected by influenza vaccination compared with the overall population.

In 2022/2023 and 2023/2024, IVE point estimates were higher for influenza B compared with the overall IVE point estimates, 68% (95% CI 18 to 91) in 2022/2023 and 84% (95% CI 53 to 97) in 2023/2024 and IVE was higher against influenza B in 2023/2024 compared with 2022/2023. In both seasons, protection against influenza A(H1N1)pdm09 was lower than the overall IVE point estimate, 17% (95% CI −57 to 58) in 2022/2023 and 30% (95% CI −13 to 58) in 2023/2024 and IVE against influenza A(H1N1)pdm09 was higher in 2023/2024 compared with 2022/2023. IVE against influenza A(H3N2)pdm09 was higher than the overall point estimate in 2022/2023 but was lower than the point estimate in 2023/2024, 55% (95% CI 8 to 79) in 2022/2023 and 25% (95% CI −6 to 47) in 2023/2024. IVE against influenza A(H3N2) was higher in 2022/2023 compared with 2023/2024.

However, all comparisons of IVE to the overall IVE point estimates and comparison of IVE between influenza seasons by age and by influenza (sub)type were limited by small sample size and a lack of precision of confidence intervals which overlapped. In particular, IVE estimates for children aged 2–17‐years contained the null value in the 2022/2023 season and IVE estimates for those aged ≥ 65 years contained the null value in both seasons.

Genetic characterisation data for the 2022/2023 and the 2023/2024 seasons in Ireland suggested that overall, the seasonal influenza vaccine was protective against the circulating virus clades detected in Ireland for all influenza (sub)types [7, 13]. However, the A(H3) viruses genetically characterised in Ireland in 2023/2024 were attributed to clade 2a.3a.1, represented by the A/Thailand/8/2022 virus and contained the signature amino acid substitutions characterised by this clade [7]. The 2023/2024 Northern Hemisphere influenza vaccine strains had reduced reactivity against viruses expressing HA genes from subclades 2a.3a.1 such as A/Thailand/8/2022 virus [14, 15]. Therefore, protection against A(H3) viruses may have been be lower in 2023/2024 [7]. This is consistent with the lower IVE against influenza A(H3N2) in 2023/2024 reported in this study.

A change in the influenza vaccine composition was recommended by the WHO for influenza A(H1N1)pdm09 in 2023/2024 and influenza A(H1N1)pdm09 was changed from 5a.2a to 5a.2a.1 [16]. Higher IVE against influenza A(H1N1)pdm09 for the 2023/2024 season compared with the 2022/2023 season has been reported internationally, potentially explained by this change in vaccine composition [16, 17]. This is consistent with the higher IVE against influenza A(H1N1)pdm09 reported in this study.

In Europe in 2022/2023, IVE of between 36% and 42% was reported in adults aged 18–64 years against presentations to primary care and emergency care settings against influenza A(H3N2) [18]. Portugal reported IVE of 44.5% (95% CI −5.6 to 70.8) against influenza A(H3N2) for QIV in adults aged ≥ 18 years [19]. These international IVE estimates are similar to the overall 55% estimate reported for the 2022/2023 season in this study. In primary and emergency care settings in Europe in 2022/2023, IVE against influenza A(H1N1)pdm09 was 49% [18]. This was higher than the 17% overall IVE estimate reported in this study. Similar to the findings of this study, pooled European estimates reported that IVE was highest against influenza B in 2022/2023 [20], an IVE of 66% was reported in older adults in a primary care and emergency care setting [18].

In children in 2022/2023, pooled European IVE estimates reported IVE of 62% against presentations to primary care and emergency care settings with influenza A(H3N2) [18]. Pooled European estimates reported IVE of 88–95% against influenza B in those aged < 18 years in 2022/2023 [18]. Thee observation of higher IVE in children internationally in 2022/2023 is consistent with the findings of this study.

In 2023/2024, interim IVE against presentation to primary care with ARI due to influenza A(H3N2) was 30% (95% CI −3 to 53) [16]. In Canada in 2023/2024, interim IVE was 40% (95% CI 5 to 61) against influenza A(H3N2) presentation to primary care [17]. These estimates are consistent with the overall end of season IVE estimate of 25% against influenza A(H3N2) that are reported in this study. In 2023/2024, pooled European interim IVE against presentation to primary care with ARI due to influenza A(H1N1)pdm09 was 53% (95% CI 41 to 63) [16], while in Canada in 2023/2024, IVE was 63% (95% CI 51 to 72) [17]. These estimates are also higher than the overall IVE estimate of 30% reported in this study. IVE of 69% in adults was reported in the United States in 2023/2024 against influenza B [21]. This aligns with the finding of higher IVE against influenza B reported in this study but is lower than the overall estimated of 84% reported here.

In 2023/2024, IVE was 85% in children against influenza A(H1N1)pdm09 [16], and IVE of 64%–89% in children was reported in the United States against influenza B [21]. Higher IVE in children reported internationally in 2023/2024 aligns with the findings of our study.

The findings of our study suggest that influenza vaccination reduced medical attendance to general practice with ARI due to influenza demonstrating the benefits of the influenza vaccination programme at supporting the protection of population health and the health service in primary care from the impact of influenza.

Despite the limitations of small sample size and overlapping confidence intervals, the findings of a higher point IVE in two seasons in children aged 2–17 years reported in this study may be useful to inform communication for future LAIV programmes, emphasising that the effectiveness of vaccination may be higher in children, reducing the proportion of children requiring medical consultation and also possibly reducing viral circulation in children and subsequent onward transmission to older age groups and those with underlying risk factors for severe infection.

Higher IVE in children can be explained by a number of factors including a better response to vaccines among those in younger age groups [22], or the national recommendation for all children aged 2–17 years to receive influenza vaccine rather than targeted vaccination to those in risk groups only [4]. It may also reflect a better performance of the LAIV compared with the QIV.

Vaccination of children has well established benefits for the broader population [23]. There was poor vaccination uptake nationally among children aged 2–17 years in the 2022/2023 and 2023/2024 influenza seasons in Ireland (15% and 16%, respectively) [6]. In the 2023/2024 influenza season in Ireland, the LAIV programme was rolled out in schools for certain class groups, specifically targeting senior infant classes (usually comprising of children aged 6–7 years). Data on vaccine uptake in the school settings are pending; however, there is strong international evidence that the delivery of vaccination in school‐based settings increases vaccination uptake [23, 24, 25, 26]. This findings of this study can support continued efforts to increase influenza vaccination coverage among children and can inform subsequent influenza vaccination programmes and vaccination communication materials.

Additionally, the observation of lower IVE in those aged ≥ 65 years is consistent with known lower IVE in older age groups due to immunosenescence [27, 28]. Adjuvanted seasonal influenza vaccines and non‐adjuvanted higher‐dose influenza vaccines have been shown to increase vaccine effectiveness in older age groups [29, 30, 31]. Most influenza related deaths occur in those aged ≥ 65 years and morbidity and mortality are reduced in older adults who are vaccinated [4, 32]. In Ireland aQIV is recommended for those aged ≥ 65 years when available [4]. However, adjuvant vaccines were not used in Ireland during the 2022/2023 or 2023/2024 influenza seasons. While in this study age‐specific analysis was limited by small sample size and wide confidence intervals, the findings of lower point IVE estimates in Ireland over two influenza seasons in those aged ≥ 65 years can be used to inform decisions about influenza vaccination recommendations and policy for older age groups. Increasing access to aQIV nationally has the potential to increase the effectiveness of vaccination for older populations and may represent an area for improvement in the national seasonal influenza vaccination programme in Ireland.

Overall, within the population, understanding the protection provided by influenza vaccines is important to inform vaccination recommendations and vaccination programmes to support preparation for future influenza seasons, including communication with the public and healthcare professionals about the benefits of vaccination. This is particularly important in the context of suboptimal influenza vaccination uptake. Continued surveillance of respiratory viruses, including influenza, is crucial to understand the epidemiological situation, including the performance of seasonal vaccination programmes to inform vaccination recommendations for the population. Ireland contributes to the estimate of pooled IVE estimates at a European level as part of the VEBIS network. This collaboration is important to ensure reliable, precise and timely IVE estimates annually for Europe to inform public health action.

Our study had several limitations. Firstly, the analysis, particularly subgroup analysis by age group and by influenza (sub)type, was limited by the small sample size. To control for this, sensitivity analyses using Firth's penalised regression were conducted. However, this limitation impacted the precision of the estimates and the accuracy of comparison of IVE estimates between influenza seasons.

Secondly, changes took place within the sentinel GP network in Ireland during the 2022/2023 influenza season. The network expanded to improve population representativeness. It had 61 GP practices in October 2022 and had expanded to 100 practices by February 2023. This changed the representatives during the study period. The study period was restricted to the time period after the change in case definition from ILI to ARI, reducing the study time period and the sample size in 2022/2023.

Thirdly, vaccination uptake was low in the study population (23% overall in both seasons). There may have been differences in those vaccinated compared with those who were unvaccinated. This may have introduced selection bias and/or positive confounding and overestimated IVE. Additionally, age specific differences reported in this study, could be explained by healthy vaccinee effect in children, as all children were recommended to receive the influenza vaccine while in those aged 18–65 years vaccination was recommended based on belonging to risk groups.

Fourthly, vaccination status was reported by GPs and verified on COVAX if the vaccination status was unknown. There may have been errors in GP reported vaccination status which may have caused non‐differential misclassification. The latter could have led to overestimation of the IVE estimates reported.

Additionally, there may have been a risk of negative confounding as high‐risk groups (people more likely to develop symptomatic infection) were more likely to be vaccinated and this may have underestimated the IVE. To minimise negative confounding, IVE estimates were adjusted for age and the presence of a chronic disease.

Finally, a correlation between influenza and COVID‐19 vaccination can introduce bias and can underestimate IVE estimates [11]. To address this, a sensitivity analysis excluding SARS‐CoV‐2 positive controls was conducted. This was done for the calculation of the overall IVE estimate for each season but was not conducted for subgroup analysis due to small sample size. However, SARS‐CoV‐2 positive controls represented 8.9% of all controls in 2022/2023 and 7.2% of all controls in 2023/2024, the risk of bias was likely low [11], and additionally, the point IVE estimate was unchanged in the 2022/2023 season and there was a 2% difference in the 2023/2024 season. This is in keeping with other international studies that have shown minimal difference in IVE estimates when excluding SARS‐CoV‐2 positive controls in sensitivity analyses [33].

## 5 Conclusion

In two influenza seasons in Ireland, influenza vaccination reduced the risk of influenza among ARI patients presenting to general practice, particularly among children, for whom LAIV was recommended. Influenza vaccination protected the population and the health service in primary care from the full impact of influenza during the 2022/2023 and 2023/2024 influenza seasons in Ireland. Promotion of the seasonal influenza vaccine to recommended groups, should remain a public health priority. The findings of this study can inform communication campaigns to support vaccine decision making. Targeted vaccination campaigns for children promoting LAIV should emphasise the effectiveness of LAIV in children.

## Author Contributions

**Louise Marron:** writing – original draft, methodology, writing – review and editing, visualization, formal analysis. **Adele McKenna:** data curation, validation, writing – review and editing. **Joan O’Donnell:** supervision, writing – review and editing, methodology. **Michael Joyce:** data curation, writing – review and editing. **Charlene Bennett:** data curation, writing – review and editing. **Jeff Connell:** data curation, writing – review and editing. **Lisa Domegan:** supervision, writing – original draft, writing – review and editing, formal analysis, methodology.

## Disclosure

This study is part of a study commissioned by the European Centre for Disease Prevention and Control (ECDC), as part of the activities referring to Framework contract N. ECDC/2021/016 ‘Vaccine Effectiveness, Burden and Impact Studies (VEBIS) of COVID‐19 and Influenza, Lot 5’ Direct Contract N. ECD.11486 ‘Developing an infrastructure and performing vaccine effectiveness studies for COVID‐19 vaccines in the EU/EEA’.

## Ethics Statement

The Health Service Executive (HSE) in Ireland classifies clinical and virological surveillance of influenza, COVID‐19, RSV and other seasonal respiratory viruses and the ongoing monitoring of vaccine effectiveness as routine operational public health activities conducted in the public interest, rather than health research. Monitoring of vaccine effectiveness (VE) can therefore be conducted without informed consent under Article 6 and Article 9 (2)(i) of the General Data Protection Regulations (GDPR). In this regard, the HSE Health Protection Surveillance Centre's (HPSC's) Information Governance Committee and Data Protection Officer (DPO) have been consulted.

The data reported to HPSC for this project included named patient data. All data collected in this project were considered private and confidential. Data security and confidentiality were maintained at all times at HPSC which is accredited for information security management IS027001.

HPSC is compliant with the requirements of data protection legislation specifically the GDPR and the Data Protection Act 2018. The VE surveillance data stored at HPSC will be anonymised after a defined period (8 years) as per the current HPSC data retention policy.

## Conflicts of Interest

The authors declare no conflicts of interest.

### Peer Review

The peer review history for this article is available at https://www.webofscience.com/api/gateway/wos/peer‐review/10.1111/irv.70023.

## Supporting information


**Figure S1.** Sentinel GP influenza‐like illness (ILI) consultation rates per 100,000 population from week 402,021 to week 202,023, Ireland.
**Figure S2.** Number of positive influenza specimes tested by the National Virus Reference Laboratory, by influenza type/subtype and week, week 402,022 to week 402,023, Ireland.
**Figure S3.** Sentinel GP influenza‐like illness (ILI) and acute respiratory infection (ARI) consultation rates per 100,000 population from week 402,022 to week 202,024, Ireland.
**Figure S4.** Number of positive influenza specimens tested by the National Virus Reference Laboratory, by influenza type/subtype and week, week 402,023 to week 402,024, Ireland.
**Figure S5.** Count of cases (*n* = 296) and controls (*n* = 798), by week of symptom onset, primary care influenza vaccine effectiveness study, week 442,022 to week 182,023, Ireland.
**Figure S6.** Count of all influenza cases (*n* = 296)* by week of onset and influenza (sub)type, primary care influenza vaccine effectiveness study, 442,022 to week 182,023, Ireland.
**Figure S7.** Count of cases (*n* = 296) with all influenza (sub)types by age group, primary care influenza vaccine effectiveness study, week 442,022 to week 182,023, Ireland.
**Figure S8.** Influenza cases by age group and by influenza (sub)type, primary care influenza vaccine effectiveness study, week 442,022 to week 182,023, Ireland.
**Figure S9.** Count of cases (*n* = 626) and controls (*n* = 2015) by week of symptom onset, primary care influenza vaccine effectiveness study, week 422,023 to week 202,024, Ireland.
**Figure S10.** Epidemiological curve of all influenza cases (*n* = 626) by (sub)type, primary care influenza vaccine effectiveness study, 422,023 to week 202,024, Ireland.
**Figure S11.** Count of cases (n = 626) all influenza (sub)types by age group, primary care influenza vaccine effectiveness study, week 422,023 to week 202,024, Ireland.
**Figure S12.** Count of influenza cases (*n* = 626) by age group and by influenza (sub)type, primary care influenza vaccine effectiveness study, week 422,023 to week 202,024, Ireland.
**Figure S13.** Influenza vaccine effectiveness (IVE) by age group, primary care IVE study Ireland 2022/2023 and 2023/2024.
**Figure S14.** Influenza vaccine effectiveness (IVE) by influenza (sub)type, primary care IVE study Ireland 2022/2023 and 2023/2024.
**Table S1.** Descriptive characteristics of cases and controls aged 2–17 years, primary care influenza vaccine effectiveness study, Ireland 2022/2023.
**Table S2.** Descriptive characteristics of cases and controls aged 18–64 years, primary care influenza vaccine effectiveness study, Ireland 2022/2023.
**Table S3.** Descriptive characteristics of cases and controls aged 65 years and older, primary care influenza vaccine effectiveness study, Ireland 2022/2023.
**Table S4.** Descriptive characteristics of cases with Influenza A(H1N1)pdm09 and controls, primary care influenza vaccine effectiveness study, Ireland 2022/2023.
**Table S5.** Descriptive characteristics of cases with Influenza A(H3N2) and controls, primary care influenza vaccine effectiveness study, Ireland 2022/2023.
**Table S6.** Descriptive characteristics of cases with Influenza B and controls, primary care influenza vaccine effectiveness study, Ireland 2022/2023.
**Table S7.** Descriptive characteristics of cases and controls age 2–17 years:Influenza A(H3N2), primary care influenza vaccine effectiveness study, Ireland 2022/2023.
**Table S8.** Descriptive characteristics of cases and controls aged 18–64 years:Influenza A(H3N2), primary care influenza vaccine effectiveness study, Ireland 2022/2023.
**Table S9.** Descriptive characteristics of cases and controls aged 65 + years:Influenza A(H3N2), primary care influenza vaccine effectiveness study, Ireland 2022/2023.
**Table S10.** Descriptive characteristics of cases and controls aged 2–17 years:Influenza A(H1N1)pdm09, primary care influenza vaccine effectiveness study, Ireland 2022/2023.
**Table S11.** Descriptive characteristics of cases and controls aged 18–64 years:Influenza A(H1N1)pdm09, primary care influenza vaccine effectiveness study, Ireland 2022/2023.
**Table S12.** Descriptive characteristics of cases and controls aged 65 + years:Influenza A(H1N1)pdm09, primary care influenza vaccine effectiveness study, Ireland 2022/2023.
**Table S13.** Descriptive characteristics of cases and controls aged 2‐17 years:Influenza B, primary care influenza vaccine effectiveness study, Ireland 2022/2023.
**Table S14.** Descriptive characteristics of cases and controls aged 18–64 years:Influenza B, primary care influenza vaccine effectiveness study, Ireland 2022/2023.
**Table S15.** Descriptive characteristics of cases and controls aged 65 + years:Influenza B, primary care influenza vaccine effectiveness study, Ireland 2022/2023.
**Table S16.** Descriptive characteristics of cases and controls aged 2–17 years, primary care influenza vaccine effectiveness study, Ireland 2023/2024.
**Table S17.** Descriptive characteristics of cases and controls aged 18–64 years, primary care influenza vaccine effectiveness study, Ireland 2023/2024.
**Table S18.** Descriptive characteristics of cases and controls aged 65 years and older, primary care influenza vaccine effectiveness study, Ireland 2023/2024.
**Table S19.** Descriptive characteristics of cases with influenza A(H1N1)pdm09 and controls, primary care influenza vaccine effectiveness study, Ireland 2023/2024.
**Table S20.** Descriptive characteristics of cases with influenza A(H3N2) and controls, primary care influenza vaccine effectiveness study, Ireland 2023/2024.
**Table S21.** Descriptive characteristics of cases with influenza B and controls, primary care influenza vaccine effectiveness study, Ireland 2023/2024.
**Table S22.** Descriptive characteristics of cases and controls age 2–17 years:Influenza A(H3N2), primary care influenza vaccine effectiveness study, Ireland 2023/2024.
**Table S23.** Descriptive characteristics of cases and controls aged 18–64 years:Influenza A(H3N2), primary care influenza vaccine effectiveness study, Ireland 2023/2024.
**Table S24.** Descriptive characteristics of cases and controls aged 65 + years:Influenza A(H3N2), primary care influenza vaccine effectiveness study, Ireland 2023/2024.
**Table S25.** Descriptive characteristics of cases and controls aged 2–17 years:Influenza A(H1N1)pdm09, primary care influenza vaccine effectiveness study, Ireland 2023/2024.
**Table S26.** Descriptive characteristics of cases and controls aged 18–64 years:Influenza A(H1N1)pdm09, primary care influenza vaccine effectiveness study, Ireland 2023/2024.
**Table S27.** Descriptive characteristics of cases and controls aged 65 + years:Influenza A(H1N1)pdm09, primary care influenza vaccine effectiveness study, Ireland 2023/2024.
**Table S28.** Descriptive characteristics of cases and controls aged 2‐17 years:Influenza B, primary care influenza vaccine effectiveness study, Ireland 2023/2024.
**Table S29.** Descriptive characteristics of cases and controls aged 18–64 years:Influenza B, primary care influenza vaccine effectiveness study, Ireland 2023/2024.
**Table S30.** Sensitivity analysis:Overall influenza vaccine effectiveness (IVE) estimates, for all age groups and all (sub)types primary care IVE study, Ireland 2022/2023 and 2023/2024, excluding SARS‐CoV‐2 positive controls.

## Data Availability

National surveillance data used for this study may identify individuals and are therefore not available.
