# Supplementary material for: Influenza Vaccine Effectiveness Against Symptomatic Influenza in Primary Care: A Test Negative Case Control Study Over Two Influenza Seasons 2022/2023 and 2023/2024 in Ireland
Source: Influenza Other Respir Viruses. 2024 Dec 2;18(12):e70023. doi: 10.1111/irv.70023 (PMC11611713; doi:10.1111/irv.70023)
Supplement: Supplementary file 1 — Figure S1. Sentinel GP influenza‐like illness (ILI) consultation rates per 100,000 population from week 402,021 to week 202,023, Ireland. Figure S2. Number of positive influenza specimes tested by the National Virus Reference Laboratory, by influenza type/subtype and week, week 402,022 to week 402,023, Ireland. Figure S3. Sentinel GP influenza‐like illness (ILI) and acute respiratory infection (ARI) consultation rates per 100,000 population from week 402,022 to week 202,024, Ireland. Figure S4. Number of positive influenza specimens tested by the National Virus Reference Laboratory, by influenza type/subtype and week, week 402,023 to week 402,024, Ireland. Figure S5. Count of cases (n = 296) and controls (n = 798), by week of symptom onset, primary care influenza vaccine effectiveness study, week 442,022 to week 182,023, Ireland. Figure S6. Count of all influenza cases (n = 296)* by week of onset and influenza (sub)type, primary care influenza vaccine effectiveness study, 442,022 to week 182,023, Ireland. Figure S7. Count of cases (n = 296) with all influenza (sub)types by age group, primary care influenza vaccine effectiveness study, week 442,022 to week 182,023, Ireland. Figure S8. Influenza cases by age group and by influenza (sub)type, primary care influenza vaccine effectiveness study, week 442,022 to week 182,023, Ireland. Figure S9. Count of cases (n = 626) and controls (n = 2015) by week of symptom onset, primary care influenza vaccine effectiveness study, week 422,023 to week 202,024, Ireland. Figure S10. Epidemiological curve of all influenza cases (n = 626) by (sub)type, primary care influenza vaccine effectiveness study, 422,023 to week 202,024, Ireland. Figure S11. Count of cases (n = 626) all influenza (sub)types by age group, primary care influenza vaccine effectiveness study, week 422,023 to week 202,024, Ireland. Figure S12. Count of influenza cases (n = 626) by age group and by influenza (sub)type, primary care influenza vaccine effectiveness study [file IRV-18-e70023-s001.docx]

# Supplementary materials

This supplementary material is supporting information for the article **Influenza vaccine effectiveness against symptomatic influenza in primary care: A test negative design case control study over two influenza seasons 2022/2023 and 2023/2024 in Ireland**

## Influenza vaccination programme in Ireland during the 2022/2023 and 2023/2024

In 2022/2023, the influenza vaccination campaign for QIV officially began on 3^rd^ October 2022 (week 40 2022) with the launch of a national communications campaign. However, deliveries of the QIV vaccine began on 19^th^ September 2022 and therefore QIV may have been administered from that date. For LAIV, the influenza vaccination programme officially began on 17^th^ October 2022 with the launch of a national communications campaign. However, deliveries of the LAIV began on 3^rd^ October 2022, therefore LAIV may have been administered from that date.

In Ireland in 2023/2024, the Autumn /Winter Vaccination Programme was launched on 02/10/2023 (Week 40 2023). The influenza vaccination campaign for the QIV was launched 02/10/2023. The LAIV programme launched on the 09/10/2023, however deliveries of the vaccine began on 02/10/2023 and therefore LAIV may have been available form that date.

In 2022/2023 and 2023/2024, the influenza vaccine was recommended for: ^3,4^

- All children aged 2 to 17 years at the time of vaccination
- Persons aged 65 years and older
- Pregnant women
- Healthcare workers (HCWs)
- Residents of nursing homes and other residential care facilities
- Cares and household contacts of people with increased medical risk
- People with regular contact with pigs, poultry or waterfowl
- Those aged 6 to 23 months and aged 18 to 64 years with:
  - Chronic heart disease including acute coronary syndrome
  - Chronic liver disease
  - Chronic neurological disease including multiple sclerosis, hereditary and degenerative disorders of the central nervous system
  - Chronic renal failure
  - Cancer patients
  - Chronic respiratory disease, including chronic obstructive pulmonary disease, cystic fibrosis, moderate or severe asthma or bronchopulmonary dysplasia
  - Diabetes mellitus
  - Down syndrome
  - Haemoglobinopathies
  - Immunosuppression due to disease or treatment, including asplenia or splenic dysfunction and cancer patients
  - Morbid obesity, i.e. body mass index of 40 or over
  - On long-term aspirin therapy (because of the risk of Reyes syndrome)
  - Any condition (e.g. cognitive dysfunction, spinal cord injury, seizure disorder, or other neuromuscular disorder) that can compromise respiratory function especially those attending special schools/day centres
  - Moderate to severe neurodevelopmental disorders such as cerebral palsy and intellectual disability

###

### Vaccination uptake

Data on vaccination uptake published by the Health Protection Surveillance Centre (HPSC) were obtained from the Health Service Executive (HSE) Integrated Information Service (ISS) influenza vaccination dashboard.^5^ Data were sourced from the COVAX system which is the national COVID-19 and influenza vaccination information system which collects vaccination data related to COVID-19 and influenza vaccines administered to the population in Ireland.^5^ For the purposes of reporting on seasonal influenza vaccination uptake, the reporting period is September to August of the following year. Rates by age groups were calculated using the National Health Intelligence Unit (NHIU) using 2021 population projection estimates.

In 2022/2023, the national influenza uptake in those ≥65 years was 76%; similar to the 2021/2022 influenza season uptake of 75%. Vaccine uptake in children aged 2 to 17 years was 15%, slightly lower than the 2021/2022 influenza season (17%). The highest influenza vaccine uptake was reported in those aged 75 years and older (87%).^5^ In 2023/2024, the national influenza uptake in those aged 65 years and older was also 76%. Vaccine uptake in children aged 2 to 17 years was 16%.^6^

## The 2022/2023 influenza season in Ireland

During the influenza season in 2022/2023 in Ireland (week 40 2022 (beginning 03/10/2022) to week 20 2023 (ending 21/05/2023)), there was a slow increase in influenza case numbers from week 40 2022.^8^ Sentinel General Practice (GP) influenza like illness (ILI) consultation rates first exceeded baseline threshold levels in week 45 2022 and remained above the threshold until week 5 2023.^8^ There was a peak in case numbers and sentinel ILI rates in week 51 2022 (Figure S1). Influenza A predominated during the season until week 4 2023, with circulation of both influenza A(H3N2) and influenza A(H1N1)pdm09 (Figure S2). There was a decrease in weekly cases until week 6 2023 however there was then an increase in influenza cases until week 13 (Figure S1). This increase was due to the circulation of influenza B which predominated later in the 2022/2023 season and peaked during weeks 10 and 13 2023 (Figure S1 and Figure S2). This resulted in a long influenza season in 2022/2023.


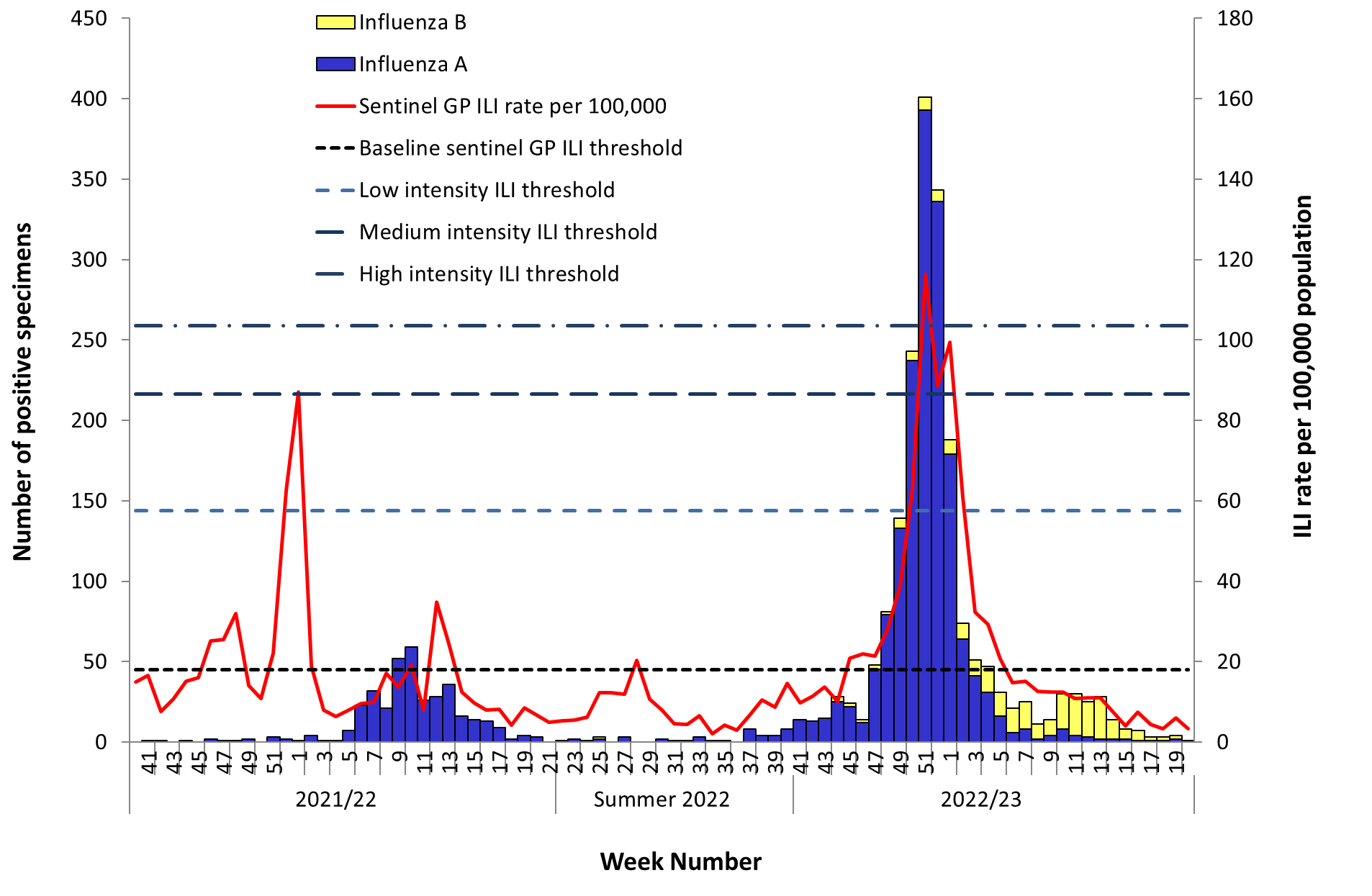


Figure S1. Sentinel GP influenza-like illness (ILI) consultation rates per 100,000 population from week 40 2021 to week 20 2023, Ireland^8^

**
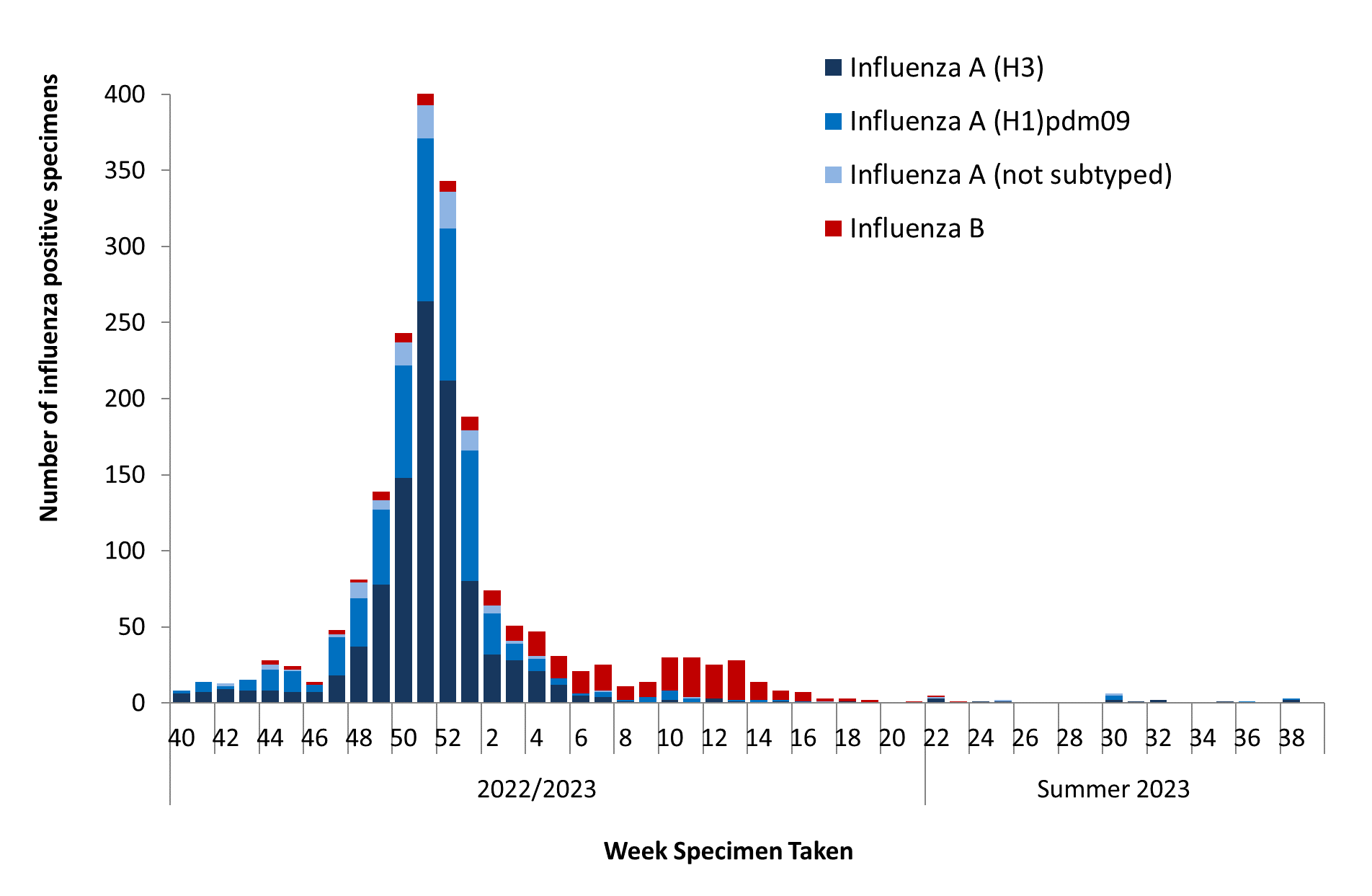
**

Figure S2. Number of positive influenza specimes tested by the National Virus Reference Laboratory, by influenza type/subtype and week, week 40 2022 to week 40 2023, Ireland^8^

## The 2023/2024 influenza season in Ireland

During the influenza season in 2023/2024 in Ireland (week 40 2023 (beginning 02/10/2023) to week 20 2024 (ending 19/05/2023), there was a slow increase in influenza case numbers from week 40 2023.^7^ There was a peak in case numbers and sentinel ILI rates in week 3 2024 (Figure S3). Influenza A predominated during the season until week 13 2024 (ending 31/03/2024), with circulation of both influenza A(H3N2) and influenza A(H1N1)pdm09, with A(H3N2) predominating (Figure S4). Influenza B circulated at lower levels than influenza A throughout the season, however from week 14 2024 (beginning 01/04/2024), there was an increase in the proportion of influenza B viruses detected (Figure S4).


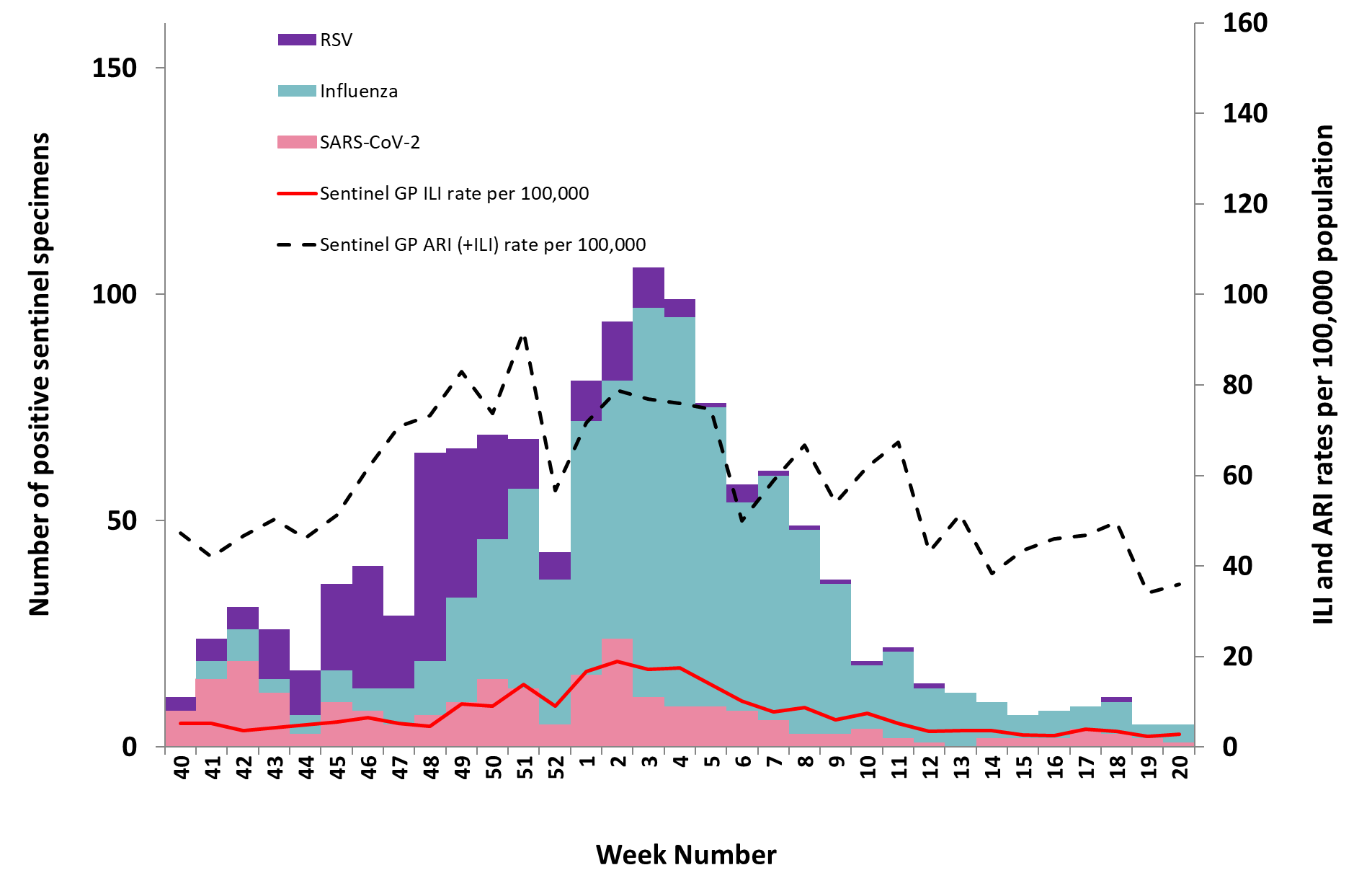


Figure S3. Sentinel GP influenza-like illness (ILI) and acute respiratory infection (ARI) consultation rates per 100,000 population from week 40 2022 to week 20 2024, Ireland^7^


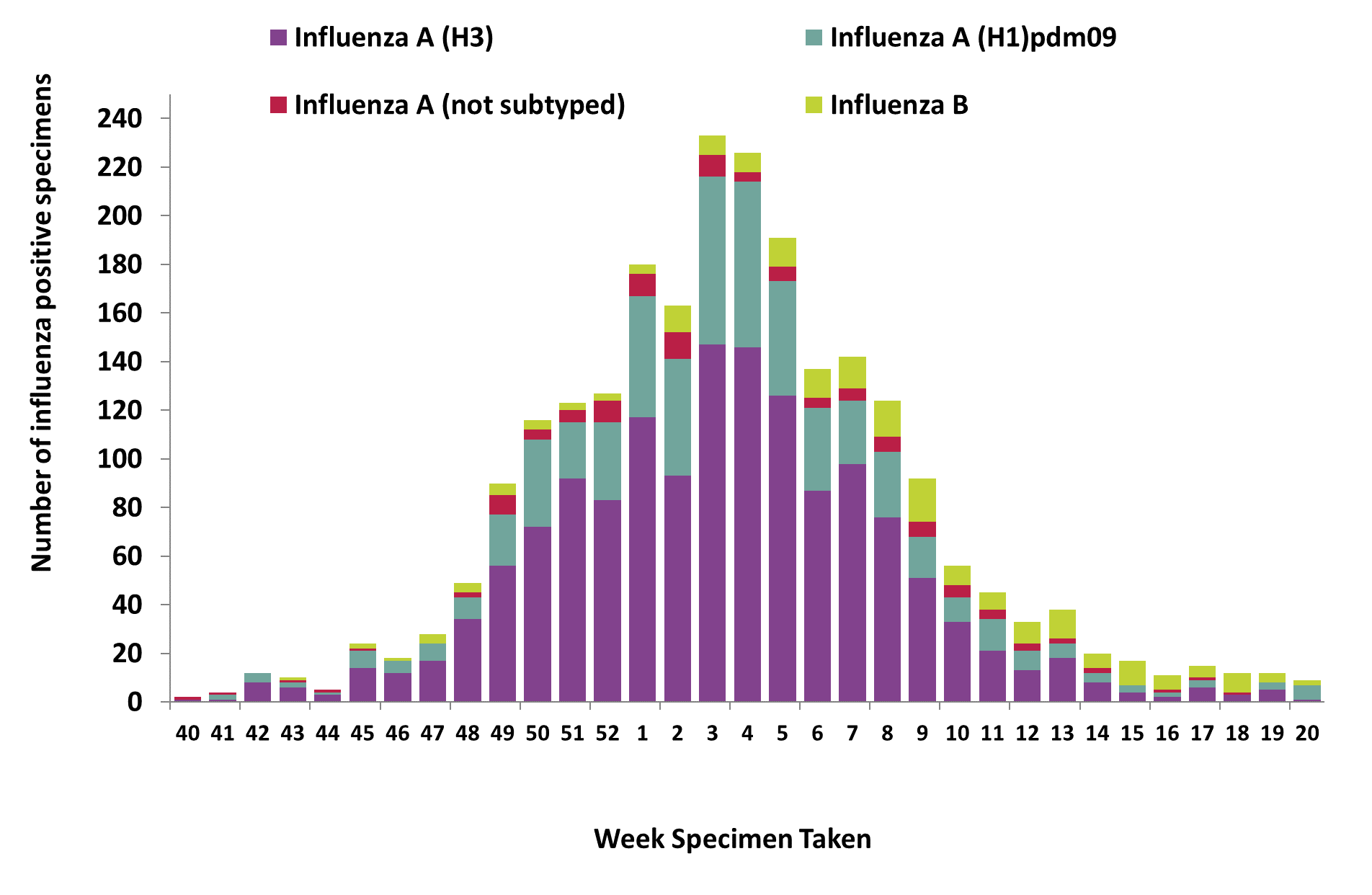


Figure S4. Number of positive influenza specimens tested by the National Virus Reference Laboratory, by influenza type/subtype and week, week 40 2023 to week 40 2024, Ireland^7^

## Inclusion criteria

Patients were included in the study if they attended GP, had a swab taken and were reported by the GP as meeting the case definition for ARI.

## Exclusion criteria

Patients were excluded from the primary analysis if they:

- Refused to participate in the study.
- Were not swabbed.
- Were unable to follow an interview because of aphasia, reduced consciousness, or other reasons.
- Could not be swabbed due to severe septum deviation, obstruction or other conditions that contra-indicate swabbing.
- Were swabbed more than 7 days after symptom onset.
- Were not reported by the GP as having met the case definition for either ARI.
- Had an inconclusive PCR test or specimen not tested (could not be classified as a case or control).
- Had tested positive before for any influenza virus in the current season.
- Had a duplicate record reporting results of two positive influenza swabs within 14 days (duplicate record excluded).
- Had received antivirals within 14 days of symptom onset.
- Did not have an influenza vaccination status documented (by GP or on COVAX).
- Did not have a date of influenza vaccination documented (by GP on COVAX).
- Attended GP with ARI after study period had ended.

# Data dictionary

| **Variable name** | **Type** | **Values and Coding** | **Definition** |
| --- | --- | --- | --- |
| ***ID variables*** | | | |
| ExtractionDate | Date | dd/mm/yyyy | Date of extraction of data |
| PatSpecID | Number | [needs to be unique] | Unique and persistent identifier for each record |
| COVID_ID | Number | [needs to be unique] | Unique identifier for each participant |
| SpecID | Number | [needs to be unique] | Unique identifier for each specimen |
| sentinelGPID | Number | [needs to be unique] | Unique identifier for each GP |
| ***Demographic variables*** | | | |
| AgeY | Numeric (continuous) | Integer | Age of each participant in years |
| age10 | Categorical | Age in 10-year age bands from 0 to 70+ | Age in 10-year age bands from 0 to 70+ |
| age_group | Categorical | 0 = 0-14  1 = 15-64  2 = 65+ | Age group with three levels (o-14 years, 15-64 years, and 65 years and older) |
| Gender | Categorical (binary) | M=male  F=female  U = Unknown | Sex of study participant |
| ***Case definition variables*** | | | |
| ARI_casedef | Categorical | Yes=ARI case No=not ARI case  Missing = No data entered | ARI case definition |
| ILI_casedef | Categorical | Yes=ILI case No=not ILI case | ILI case definition |
| ari | Categorical | 0 = Not ARI or ILI case  1 = ARI or ILI case  NA = Missing/Not classified as either ARI or ILI | Those with missing in ARI_casedef variable and those with No in the ARI who are recorded as ILI = Yes recoded as ARI = Yes  Any patient recorded as ILI also meet case definition for ARI and are recoded as ARI. |
| **Variable name** | **Type** | **Values and Coding** | **Definition** |
| ***Variables related to symptom onset, specimen collection*** | | | |
| GP referral date | Date | dd/mm/yyyy | GP referral date |
| Fluswab_request | Categorical | Yes=requested No=not requested | Flu swab request |
| gp_ref_date | Date | yyyy-mm-dd | Date recoded as Date format in R (no missing or implausible values in this variable) |
| DateSpecimenTaken | Date | dd/mm/yyyy | Date of specimen taken to test |
| swabdate | Date | yyyy-mm-dd | Date recoded as Date format in R (no missing or implausible values in this variable) |
| swabdelay | Numeric | Number of days (0 - 7 days) | Number of days between symptom onset and swab date |
| TestType | Categorical | Flu+= Flu+ test SARS-CoV-2 only=only SARS-CoV-2 test | Test Type |
| dateofonset | Date | dd/mm/yyyy | Date of onset of symptoms |
| onsetdate | Date | yyyy-mm-dd | Date recoded as Date format in R (no missing or implausible values in this variable) |
| onsetweek | Character | yyyy-ww | Week of onset (isoweek) |
| onsetmonth | Categorical | Numeric (1-12 by month of year January-December) | Month of onset |
| onsetmonth2 | Categorical | 0 = Oct-Dec 2023  1 = Jan-March 2024  2 = April-May 2024 | Month of onset as three categories |
| ***Symptom variables*** | | | |
| symp_all | Text |  | Listing all symptoms |
| symp_suddenonset | Text |  | Sudden onset |
| symp_fever | Text |  | Fever |
| symp_cough | Text |  | Cough |
| symp_sob | Text |  | Shortness of breath |
| symp_smelltaste | Text |  | Anosmia (Loss of sense of smell) or Aguesia/dysgeusia (Loss or distortion of sense of taste) |
| **Variable name** | **Type** | **Values and Coding** | **Definition** |
| symp_headache | Text |  | Headache |
| symp_sorethroat | Text |  | Sore throat |
| symp_myalgia | Text |  | Myalgia |
| symp_malaise | Text |  | Malaise |
| symp_other | Text |  | Other symptoms |
| antiviraldrugs_14d | Categorical | 1 = Yes/exposed 0 = No/not exposed NA = Unknown | Exposed to antiviral drugs in 14 days prior to consultation |
| ***Risk groups/underlying condition variables*** | | | |
| pregnant | Categorical | 0 = No/not pregnant 1 = Yes/pregnant NA = Unknown | Pregnancy status |
| BMI | Categorical | BMI > = 40= if BMI equal to 40 or over  BMI 30-39= if BMI between 30-39  N/A = Not applicable  Unknown=unknown | BMI category |
| Medical risk group unspecified | Text |  | Risk groups |
| chroniccon | Categorical | Yes=having chronic condition No=not having chronic condition | Any chronic condition |
| anychron | Categorical | 0 = No chronic condition  1 = Chronic condition  NA = Unknown/missing | 0 = No chronic condition (chronicon = No)  1 = At least one chronic condition (chronicon = Yes or Pregnant = Yes or BMI > 40 = Yes or Medical_risk_group_unspecified = At risk group |
| chroniccon_all | Text |  | Listing all medical conditions |
| chron_cancer | Text |  | Cancer |
| chron_heart | Text |  | Chronic heart disease |
| chron_kidney | Text |  | Chronic kidney disease |
| chron_liver | Text |  | Chronic liver disease |
| chron_neuro | Text |  | Chronic neurological disease/condition |
| **Variable name** | **Type** | **Values and Coding** | **Definition** |
| chron_resp | Text |  | Chronic respiratory disease (includes asthma) |
| chron_diabetes | Text |  | Diabetes mellitus (type 1 & 2) |
| chron_immuno | Text |  | Immunocompromised (disease/treatment) |
| chron_intelldis | Text |  | Intellectual disability |
| chron_severedep | Text |  | Severe mental illness |
| chron_other | Text |  | Other medical conditions |
| ***Classification as case or control variables*** | | | |
| PositiveInfluenzaResult | Categorical | 0=negative 1=positive 9=not tested/inhibitory/equivocal  Blank = no data entered | Influenza test result |
| case | Categorical | 0 = control  1 = case  9 = NA  Blank = NA | Case variable classifying those negative for any influenza as controls, those positive for any influenza as cases and those with a not tested/inhibitory/equivocal or those that are blank are NA (missing) |
| ***Influenza laboratory results variables*** | | | |
| result | Text |  | Name of respiratory pathogen |
| Coinfection | Text |  | Coinfection |
| flu_coinfection | Categorical | 0 = Control  1 = Infected  2 = Coinfected  NA = Missing/blank | Classifying those infected with > 1 influenza subtype  0 = Control = No positive influenza result  1 = Infected = specimen positive for one influenza subtype  2 = Coinfected = specimen positive for > 1 influenza subtype |
| Result2 | Text |  | If there is coinfection, name of the pathogen |
| Result3 | Text |  | If there is coinfection, name of the pathogen |
| Result4 | Text |  | If there is coinfection, name of the pathogen |
| Result5 | Text |  | If there is coinfection, name of the pathogen |

| **Variable name** | **Type** | **Values and Coding** | **Definition** |
| --- | --- | --- | --- |
| ***Influenza vaccination variables*** | | | |
| influenzavaccine_complete | Categorical | Yes=completed No=not completed Unknown=unknown | Current seasonal influenza vaccine status |
| influenzavaccine_complete2 | Categorical | Yes=completed No=not completed Unknown=unknown  No vaccine record = Vaccine record could not be sourced | Current seasonal influenza vaccine status after validation |
| influenzavaccine_date | Date | dd/mm/yyyy  Missing = no date or implausible date | Date of influenza vaccine |
| fluvacc | Categorical | 0 = Not vaccinated  1 = Vaccinated  NA = excluded | Not vaccinated: influenza vaccination not completed or vaccinated on or after date of symptom onset  Vaccinated: influenza vaccination completed 14 or more days before symptom onset  NA: Unknown vaccination status or vaccinated with symptom onset between 1-13 days after vaccination or date of vaccination is missing |
| fluvaccdate | Date | yyyy-mm-dd  NA = missing/implausible date | Recoded as date in R with implausible dates corrected or coded as NA |
| fluvaccweek | Date | yyyy-ww | Week of influenza vaccination (isoweek) |
| influenzavaccine_type | Categorical | 0 = LAIV  1 = QIV  2 = Other  NA = Missing | Influenza vaccine type  LAIV = Live attenuated influenza vaccine  QIV = Quadrivalent influenza vaccine  Other = Other vaccine/not specified |
| fluvaccdelay | Numeric | Number of days | Number of days between influenza vaccine and symptom onset |

| **Variable name** | **Type** | **Values and Coding** | **Definition** |
| --- | --- | --- | --- |
| ***Influenza subtype variables*** | | | |
| caseh3 | Categorical | 0 = control  1 = case_ah3  NA =missing, unknown, excluded | 0 = Not an influenza case  1 = Positive for Influenza A (H3N2)  NA = Positive for another influenza subtype in 2023/2024 season or missing/unknown influenza result |
| Caseh1 | Categorical | 0 = control  1 = case_ah1  NA =missing, unknown, excluded | 0 = Not an influenza case  1 = Positive for Influenza A (H1N1)pdm09  NA = Positive for another influenza subtype in 2023/2024 season or missing/unknown influenza result |
| caseb | Categorical | 0 = control  1 = case_b  NA =missing, unknown, excluded | 0 = Not an influenza case  1 = Positive for Influenza b  NA = Positive for another influenza subtype in 2023/2024 season or missing/unknown influenza result |
| Flu_subtype | Categorical | 0 = control  1 = influenza A not subtyped  2 = influenza A (H3N2)  3 = Influenza A (H1N1)pdm09  4 = Influenza B/lineage unknown  NA = missing/no result | Classification based on subtypes reported in result, result 2 and result 3 variables. |

**Exclusions and restrictions for each season**

Restriction flow chart for primary analysis, influenza vaccine effectiveness study, Ireland 2022/2023

| **Restriction flow chart for primary analysis** | **n** |
| --- | --- |
| Total records in sentinel GP dataset for 2022/2023 influenza season | 1,747 |
| Excluded duplicate records with incomplete data | 1,745 |
| Excluded duplicate records with two positive influenza swabs within 14 days^†^ | 1,743 |
| Excluded records where GP had not stated that patient met either ARI or ILI case definition | 1,717 |
| Excluded anyone swabbed before influenza case definition changed from ILI to ARI on 09/11/2022 | 1,685 |
| Excluded ARI cases after week 19 2023 (last influenza case had onset in week 18 2023) | 1,522 |
| Excluded those swabbed more than 7 days after symptom onset | 1,323 |
| Excluded those without an influenza vaccination date | 1,300 |
| Excluded those who received antiviral drugs within 14 days of consultation | 1,294 |
| Excluded those without a case or control classification^‡^ | 1,094 |
| Excluded those whose vaccination status was unknown^§^ | 1,086 |
| Excluded those who had unknown status for an underlying chronic condition^§^ | 1,054 |
| Excluded those who had unknown status for sex**^§^** | 1,053 |

^†^ Second record excluded, first test result and record included in the analysis

^‡^This is the total number Included in initial descriptive analysis (n = 1,094), those with no case/control classification had no valid test or no result reported or result report as inhibitory or equivocal

**^§^** Included for the complete case analysis for crude, stratified and multivariable analysis for primary analysis (n = 1,053)

Restriction flow chart for primary analysis, influenza vaccine effectiveness study, Ireland 2023/2024

| **Restriction flow chart for primary analysis** | **n** |
| --- | --- |
| Total records in sentinel GP dataset for 2023/2024 influenza season analysis | 3,336 |
| Excluded participants with two swabs taken within 14 days^†^ | 3,333 |
| Excluded records where GP had not stated that patient met ARI case definition | 3,273 |
| Excluded records before study period began in week 42 2023 | 3,056 |
| Excluded those swabbed more than 7 days after symptom onset | 2,665 |
| Excluded those without an influenza vaccination date | 2,661 |
| Excluded those who received antiviral drugs within 14 days of consultation | 2,647 |
| Excluded those without a case or control classification^‡^ | 2,641 |
| Excluded those whose vaccination status was unknown**^§^** | 2,606 |
| Excluded those who had unknown status for an underlying chronic condition | 2,400 |
| Excluded those who had unknown status for sex**^§^** | 2,399 |

^†^Second record excluded, first test result and record included in the analysis

^‡^ Those with no case/control classification had no valid test or no result reported or result report as inhibitory or equivocal. Included in initial descriptive analysis (n = 2,641).

**^§^** Included for the complete case analysis for crude, stratified and multivariable analysis for primary analysis (n = 2,399)

**Epidemiological curves 2022/2023**

The peak in ARI infections among cases and controls was in Week 51 2022 (beginning 19/12/2022) (Figure S5) as was the peak in cases (Figure S6). From Week 44 2022 (beginning 31/10/2022) until Week 2 2023 (ending 15/01/2023), influenza A was the dominant circulating influenza type, with influenza subtypes A(H3N2) and A(H1N1)pdm09 circulating. From Week 4 2023 (beginning 23/01/2023) until the end of the study period (Week 18 2023, ending 07/05/2023), influenza B was the dominant influenza type circulating (Figure S6). Among cases aged 2-17 years, and in those aged 18-64 years, influenza A(H1N1)pdm09 and influenza A(H3N2) were the dominant circulating sub(types) until week 4 2023 (ending 29/01/2023) when influenza B became the dominant circulating influenza virus type(subtype) (Figure S7).

**
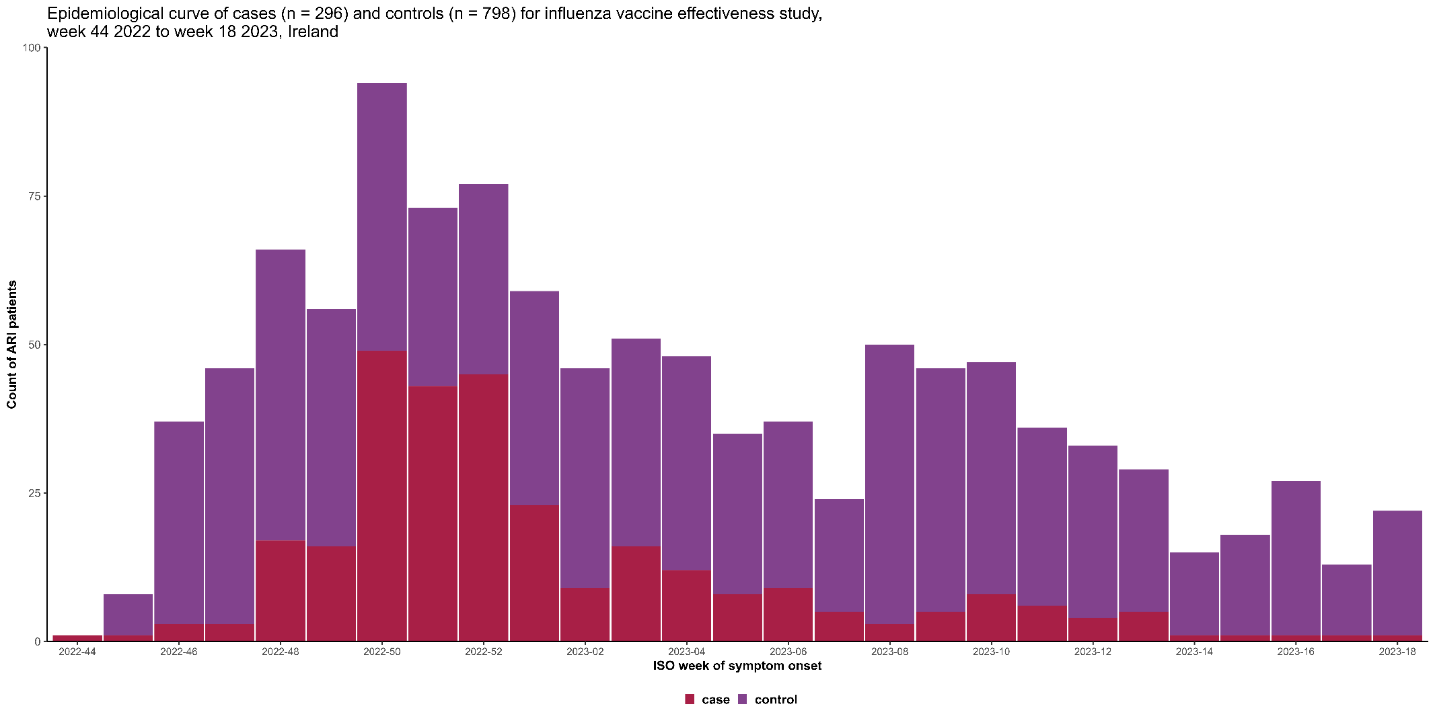
**

Figure S5. Count of cases (n = 296) and controls (n = 798), by week of symptom onset, primary care influenza vaccine effectiveness study, week 44 2022 to week 18 2023, Ireland

**
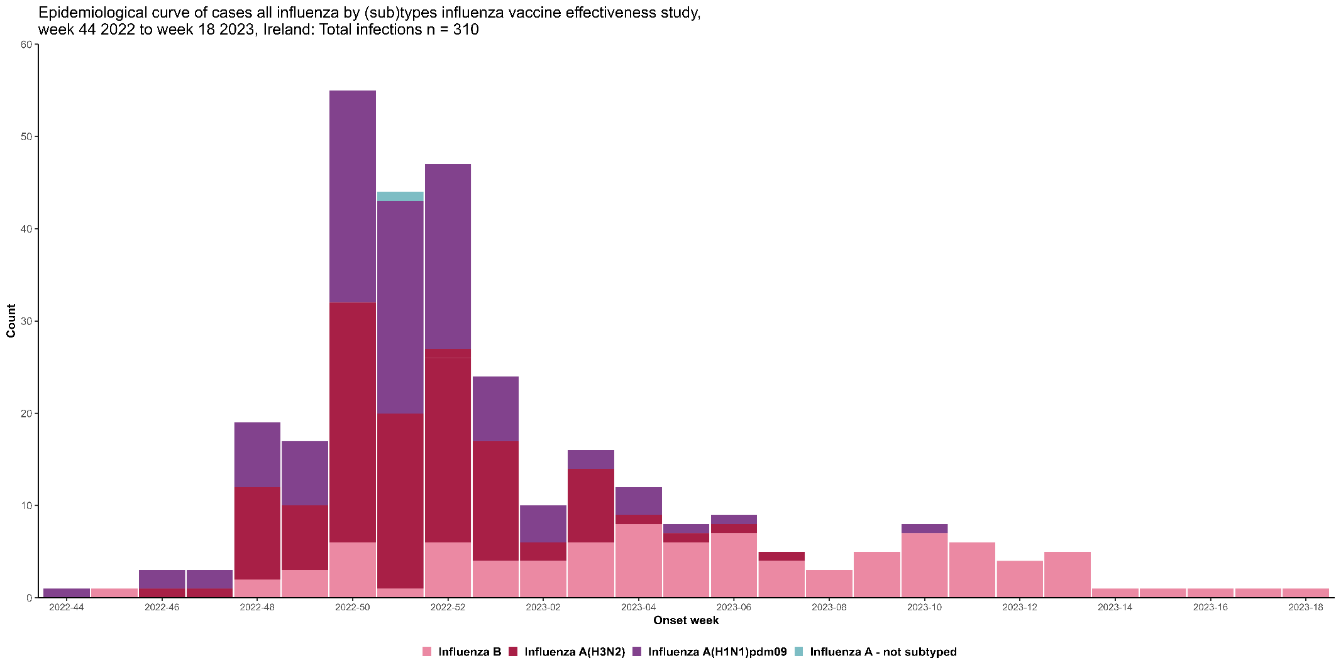
**

****Including co-infections, the total number of influenza infections shown is n = 310***

Figure S6. Count of all influenza cases (n = 296)* by week of onset and influenza (sub)type, primary care influenza vaccine effectiveness study, 44 2022 to week 18 2023, Ireland

**
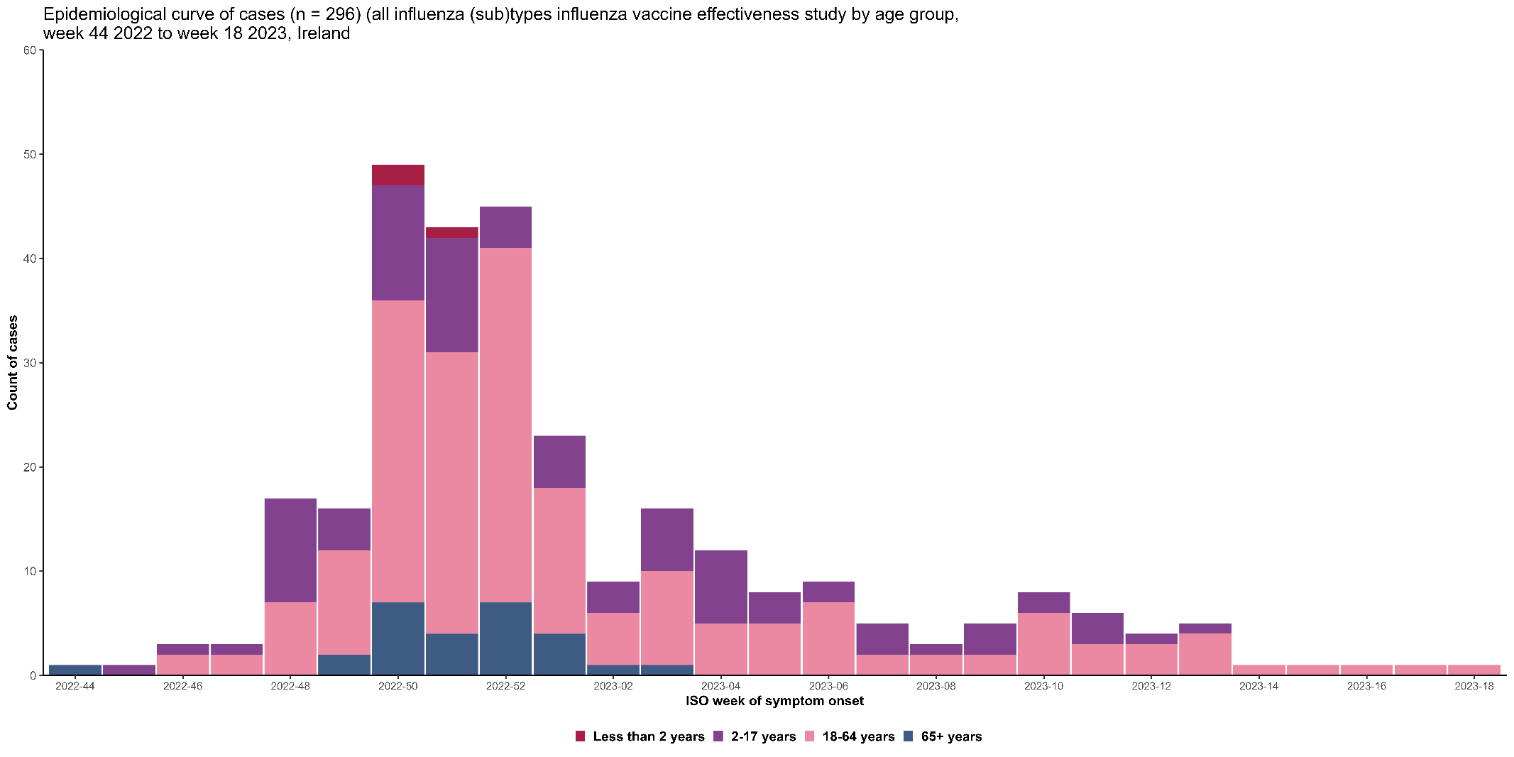
**

Figure S7. Count of cases (n = 296) with all influenza (sub)types by age group, primary care influenza vaccine effectiveness study, week 44 2022 to week 18 2023, Ireland

**
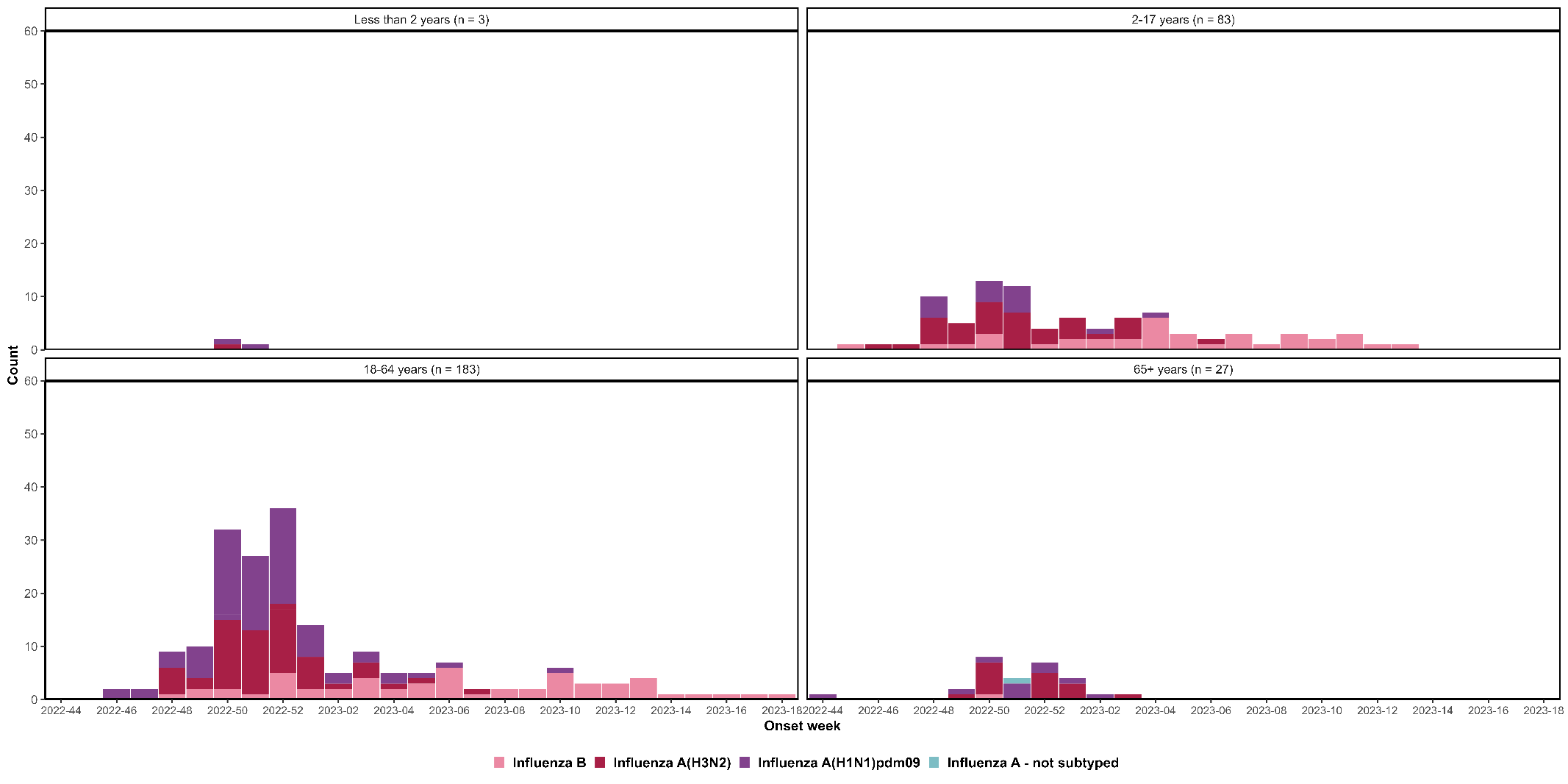
**

Figure S8. Influenza cases by age group and by influenza (sub)type, primary care influenza vaccine effectiveness study, week 44 2022 to week 18 2023, Ireland

**Epidemiological curves 2023/2024**

The peak in ARI infections among cases and controls was in Week 1 2024 (beginning 01/01/2024) (Figure S9). The peak in cases was in Week 3 2024 (ending 21/01/2024) (Figure S10). Influenza A has been the dominant circulating influenza virus in the 2023/2024 season, with both influenza A(H3N2) and A(H1N1)pdm09 viruses circulating (Figure S10); influenza A(H3N2) has predominated. Influenza B has also circulated but at lower levels during the 2023/2024 season compared to the proportion of cases attributable to influenza A in all age groups (Figures S10 and S11).


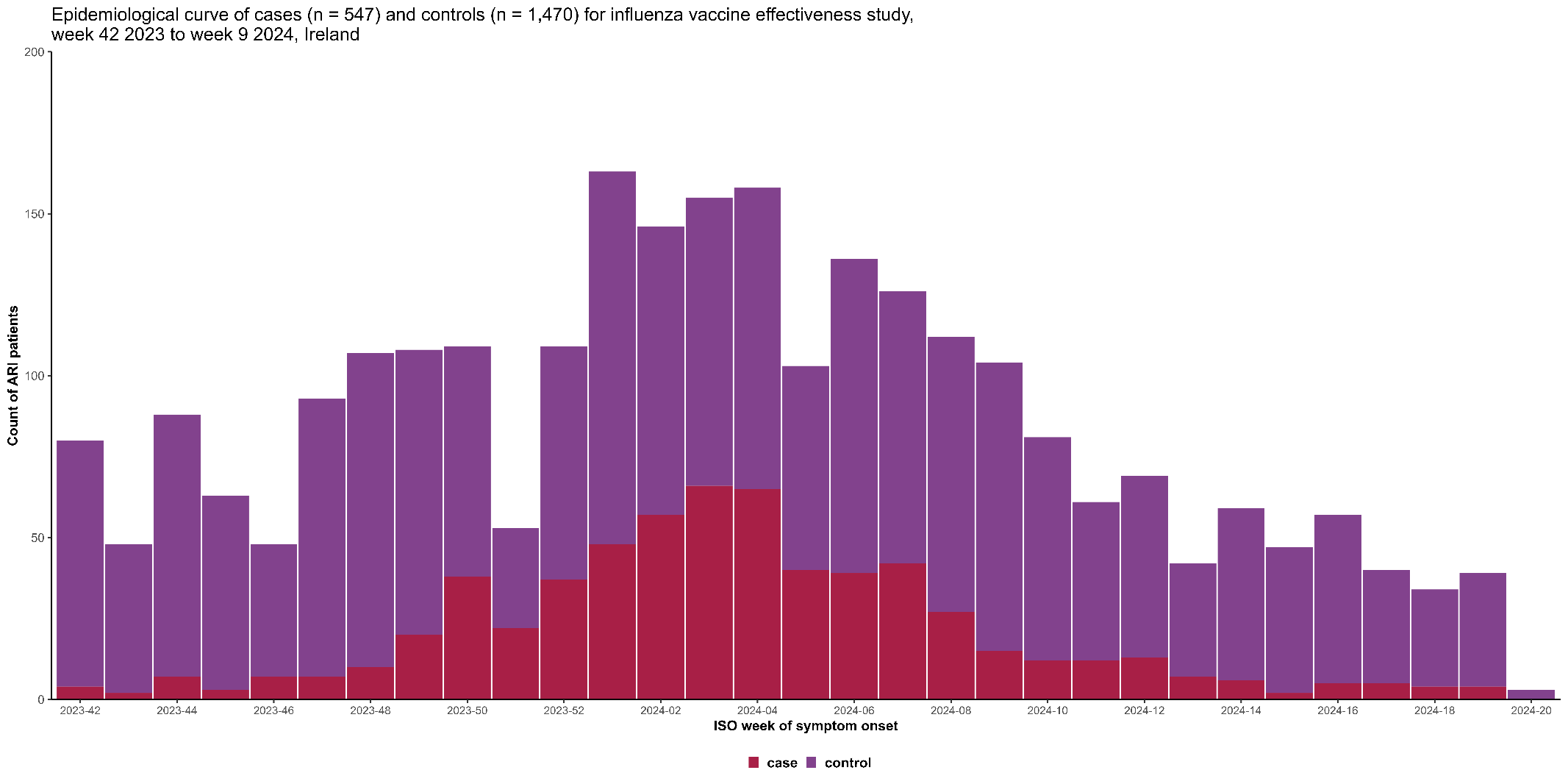


Figure S9. Count of cases (n = 626) and controls (n = 2,015) by week of symptom onset, primary care influenza vaccine effectiveness study, week 42 2023 to week 20 2024, Ireland


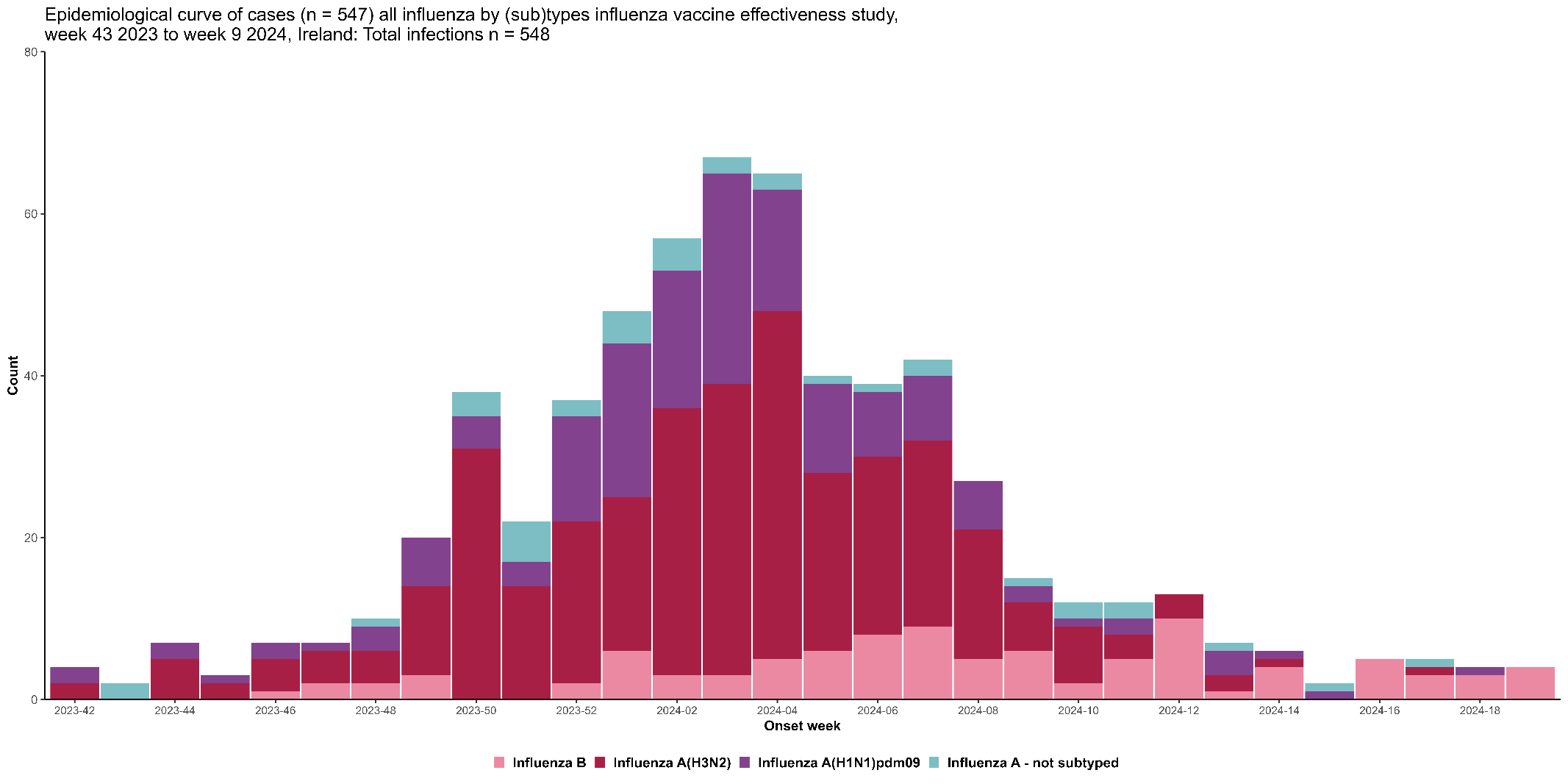


Figure S10. Epidemiological curve of all influenza cases (n = 626) by (sub)type, primary care influenza vaccine effectiveness study, 42 2023 to week 20 2024, Ireland

**
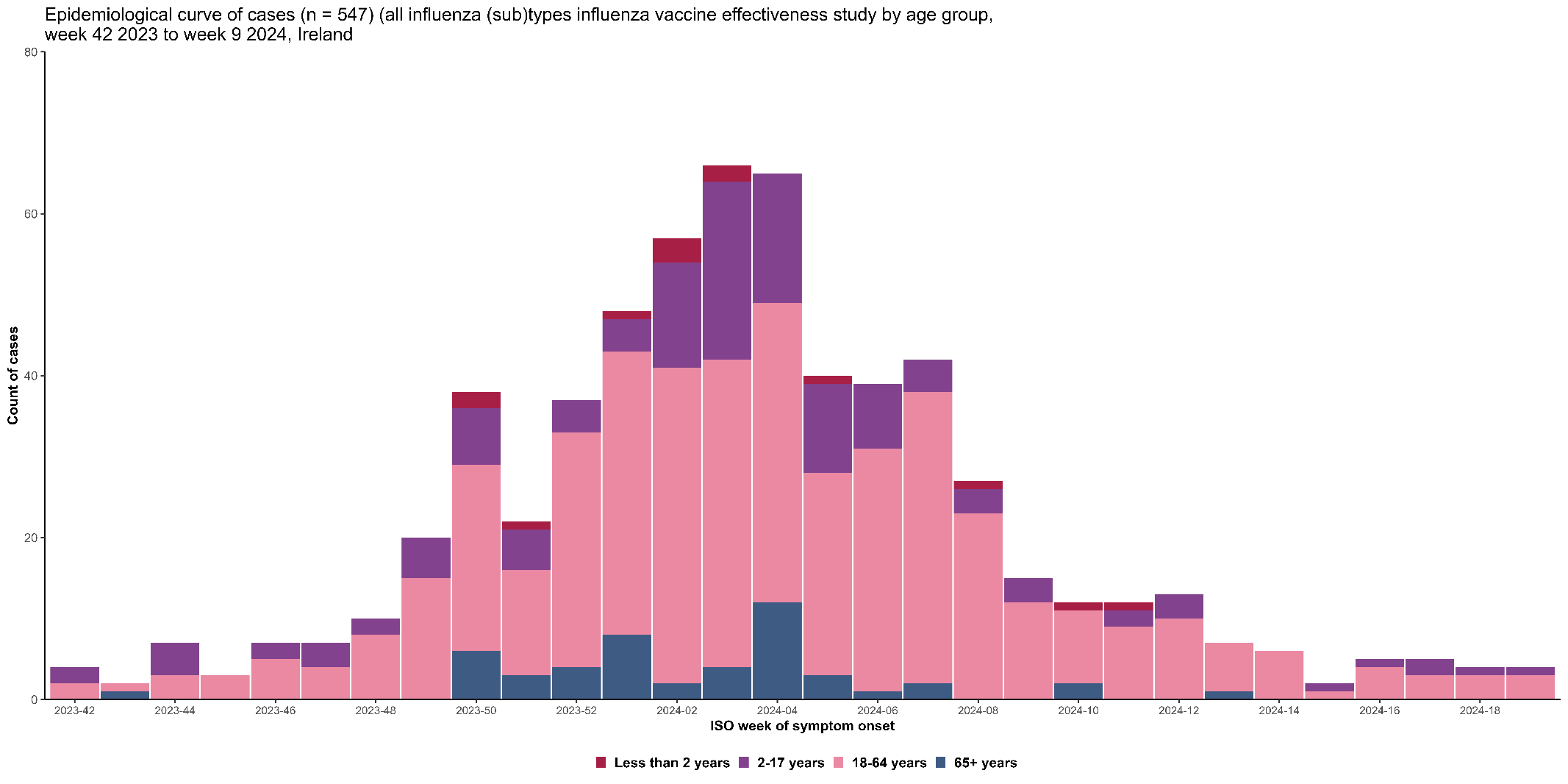
**

Figure S11. Count of cases (n = 626) all influenza (sub)types by age group, primary care influenza vaccine effectiveness study, week 42 2023 to week 20 2024, Ireland

**
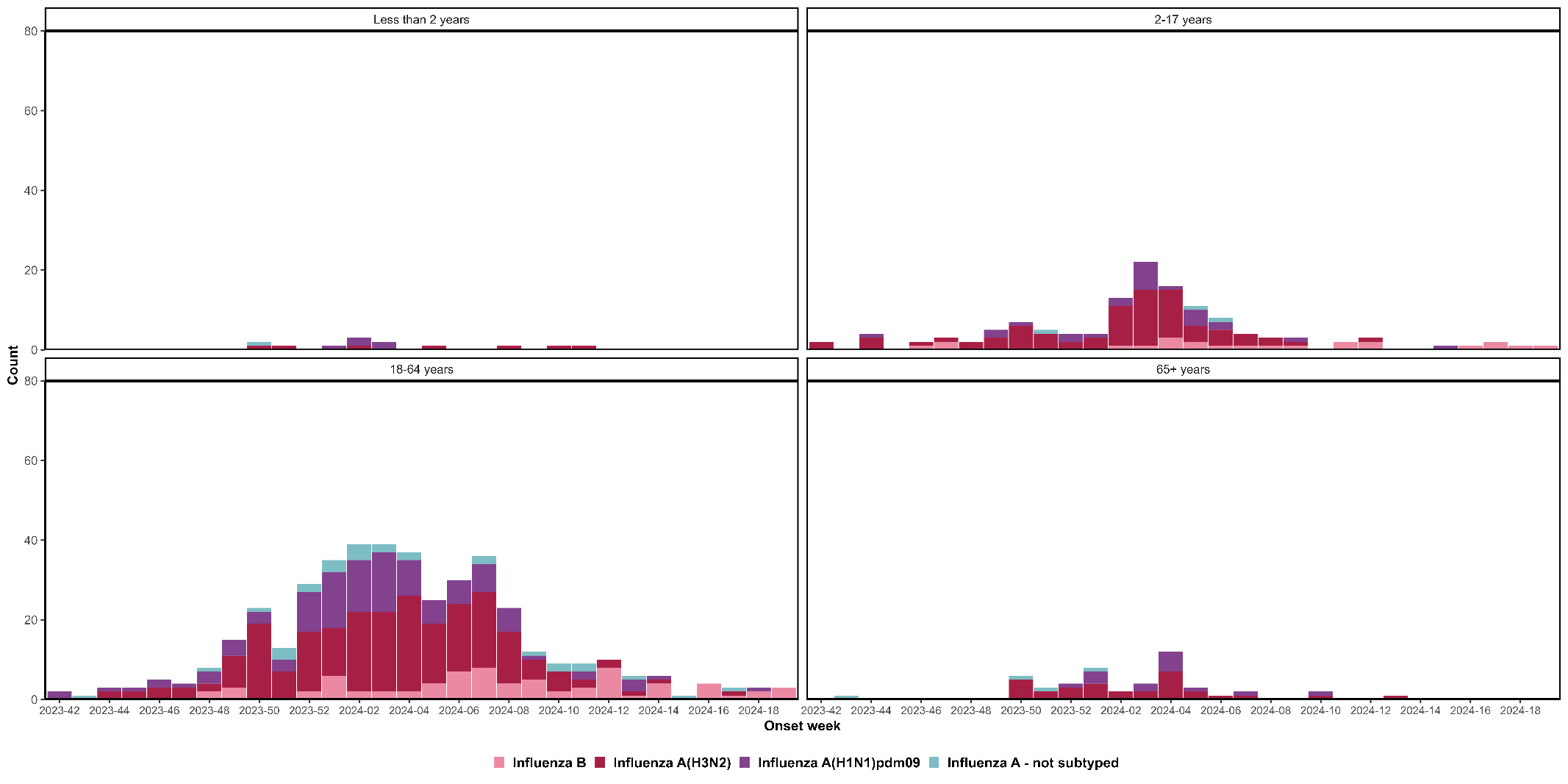
**

Figure S12. Count of influenza cases (n=626) by age group and by influenza (sub)type, primary care influenza vaccine effectiveness study, week 42 2023 to week 20 2024, Ireland

# Descriptive analysis by age group and influenza (sub)type

**Table S1. Descriptive characteristics of cases and controls aged 2-17 years, primary care influenza vaccine effectiveness study, Ireland 2022/2023**

|  | **Cases**  **(n = 83/302, 27.5%)** | | **Controls**  **(n = 219/302, 72.5%)** | |  |
| --- | --- | --- | --- | --- | --- |
| **Exposure** | **Median** | **IQR** | **Median** | **IQR** | **p-value^†^** |
| **Age (years)** | 12 | 7-14 | 7 | 4-11.5 | **<0.001** |
| **Time from symptom onset to swab (days)** | 3 | 2-4 | 3 | 2-4 | **0.046** |
| **Exposure** | **n** | **%** | **n** | **%** | **p-value^‡^** |
| **Sex** |  |  |  |  |  |
| Female | 39 | 47 | 113 | 51.8 | 0.534 |
| Male | 44 | 53 | 105 | 48.2 |  |
| Missing | 0 | 0 | 1 | 0 |  |
| **Vaccination status** | |  |  |  |  |
| Unvaccinated | 77 | 93.9 | 194 | 89 | 0.287 |
| Vaccinated | 5 | 6.1 | 24 | 11 |  |
| Missing^§^ | 1 | 0 | 1 | 0 |  |
| **Chronic condition** | |  |  |  |  |
| No chronic condition | 76 | 91.6 | 178 | 83.6 | 0.113 |
| Chronic condition | 7 | 8.4 | 35 | 16.4 |  |
| Missing | 0 | 0 | 6 | 0 |  |
| **Month of symptom onset** | | |  |  |  |
| November-December 2022 | 42 | 50.6 | 79 | 36.1 | **0.003** |
| January-February 2023 | 31 | 37.3 | 72 | 32.9 |  |
| March-May 2023 | 10 | 12 | 68 | 31 |  |
| **Time from symptom onset to swab (days)** | | | |  |  |
| 0 | 2 | 2.4 | 3 | 1.4 | 0.519 |
| 1 | 21 | 25.3 | 36 | 16.4 |  |
| 2 | 25 | 30.1 | 56 | 25.6 |  |
| 3 | 13 | 15.7 | 53 | 24.2 |  |
| 4 | 11 | 13.3 | 38 | 17.4 |  |
| 5 | 5 | 6 | 15 | 6.8 |  |
| 6 | 3 | 3.6 | 10 | 4.6 |  |
| 7 | 3 | 3.6 | 8 | 3.7 |  |
| **Influenza vaccine type** | |  |  |  |  |
| Live Attenuated Influenza Vaccine (LAIV) | 5 | 6.1 | 21 | 9.6 | 0.342 |
| Quadrivalent Influenza Vaccine (QIV) | 0 | 0 | 3 | 1.4 |  |
| Unvaccinated | 77 | 93.9 | 194 | 89.0 |  |
| Missing^§^ | 1 | - | 1 | - |  |
| **Influenza (sub)type** | |  |  |  |  |
| Control | - | - | 219 | 100 | - |
| Coinfection Influenza A (H3)/Influenza B | 3 | 3.6 | - | - |  |
| Coinfection Influenza A (H3)/influenza A(H1N1)pdm09 | 1 | 1.2 | - | - |  |
| Coinfection Influenza A (H3)/influenza A(H1N1)pdm09/Influenza B | 1 | 1.2 | - | - |  |
| Influenza A(H1N1)pdm09 | 13 | 15.7 | - | - |  |
| Influenza A(H3N2) | 32 | 38.6 | - | - |  |
| Influenza B | 33 | 39.8 | - | - |  |

**^†^***Mann Whitney U test*

**^‡^***Chi squared test or Fisher’s exact test when cell counts < 5*

^§^*Missing as vaccinated 1-13 days before onset of symptoms*

**Table S2. Descriptive characteristics of cases and controls aged 18-64 years, primary care influenza vaccine effectiveness study, Ireland 2022/2023**

|  | **Cases**  **(n = 183/595, 30.8%)** | | **Controls**  **(n = 412/595, 69.2%)** | |  |
| --- | --- | --- | --- | --- | --- |
| **Exposure** | **Median** | **IQR** | **Median** | **IQR** | **p-value^†^** |
| **Age (years)** | 40 | 29-47 | 41 | 30-51 | 0.144 |
| **Time from symptom onset to swab (days)** | 3 | 2-4 | 3 | 2-4 | **0.037** |
| **Exposure** | **n** | **%** | **n** | **%** | **p-value^‡^** |
| **Sex** |  |  |  |  |  |
| Female | 104 | 56.8 | 272 | 66 | **0.040** |
| Male | 79 | 43.2 | 140 | 34 |  |
| **Vaccination status** | | | | | |
| Unvaccinated | 164 | 89.6 | 325 | 80 | **0.006** |
| Vaccinated | 19 | 10.4 | 81 | 20 |  |
| Missing^§^ | 0 | - | 6 | - |  |
| **Chronic condition** | | | | | |
| No chronic condition | 139 | 79.0 | 289 | 72.6 | 0.131 |
| Chronic condition | 37 | 21.0 | 109 | 27.4 |  |
| Missing | 7 | - | 14 | - |  |
| **Month of symptom onset** | | | | | |
| November-December 2022 | 105 | 57.4 | 130 | 31.6 | **<0.001** |
| January-February 2023 | 55 | 30.1 | 161 | 39.1 |  |
| March-May 2023 | 23 | 12.6 | 121 | 29.4 |  |
| **Time from symptom onset to swab (days)** | | | | | |
| 0 | 3 | 1.6 | 7 | 1.7 | 0.528 |
| 1 | 28 | 15.3 | 50 | 12.1 |  |
| 2 | 44 | 24 | 81 | 19.7 |  |
| 3 | 48 | 26.2 | 105 | 25.5 |  |
| 4 | 26 | 14.2 | 72 | 17.5 |  |
| 5 | 19 | 10.4 | 41 | 10 |  |
| 6 | 5 | 2.7 | 18 | 4.4 |  |
| 7 | 10 | 5.5 | 38 | 9.2 |  |
| **Influenza vaccine type** | | | | | |
| Live Attenuated Influenza Vaccine | 0 | 0 | 2 | 0.5 | **0.031** |
| Other | 1 | 0.5 | 8 | 2.0 |  |
| Quadrivalent Influenza Vaccine | 18 | 9.8 | 71 | 17.5 |  |
| Unvaccinated | 164 | 89.6 | 325 | 80.0 |  |
| Missing^§^ | 0 | - | 6 | - |  |
| **Pregnant** | | | | | |
| No | 174 | 96.7 | 397 | 98.3 | 0.364 |
| Yes | 6 | 3.3 | 7 | 1.7 |  |
| Missing | 3 | - | 8 | - |  |
| **Influenza (sub)type** | | | | | |
| Control | - | - | 412 | 100 | - |
| Coinfection Influenza A (H3)/Influenza B | 4 | 2.2 | - | - |  |
| Coinfection Influenza A (H3)/ Influenza A(H1N1)pdm09 | 1 | 0.5 | - | - |  |
| Coinfection Influenza A (H3)/influenza A(H1N1)pdm09/ Influenza B | 1 | 0.5 | - | - |  |
| Influenza A(H1N1)pdm09 | 75 | 41 | - | - |  |
| Influenza A(H3N2) | 52 | 28.4 | - | - |  |
| Influenza B | 50 | 27.3 | - | - |  |

**^†^***Mann Whitney U test*

**^‡^***Chi squared test or Fisher’s exact test when cell counts < 5*

^§^*Missing as vaccinated 1-13 days before onset of symptoms*

**Table S3. Descriptive characteristics of cases and controls aged 65 years and older, primary care influenza vaccine effectiveness study, Ireland 2022/2023**

|  | | **Cases**  **(n =27/152 17.8%)** | | **Controls**  **(n = 125/152, 82.2%)** | | |  |
| --- | --- | --- | --- | --- | --- | --- | --- |
| **Exposure** | | **Median** | **IQR** | **Median** | **IQR** | | **p-value^†^** |
| **Age (years)** | | 69 | 67-73 | 73 | 69-80 | | **0.004** |
| **Time from symptom onset to swab (days)** | | 3 | 2-4 | 3 | 2-4 | | 0.872 |
| **Exposure** | | **n** | **%** | **n** | **%** | | **p-value^‡^** |
| **Sex** | |  |  |  |  | |  |
| Female | | 14 | 51.9 | 75 | 60 | | 0.573 |
| Male | | 13 | 48.1 | 50 | 40 | |  |
| **Vaccination status** | | | | | | | |
| Unvaccinated | | 9 | 33.3 | 26 | 20.8 | | 0.250 |
| Vaccinated | | 18 | 66.7 | 99 | 79.2 | |  |
| **Chronic condition** | | | | | | | |
| No chronic condition | | 10 | 37 | 42 | 34.7 | | 0.995 |
| Chronic condition | | 17 | 63 | 79 | 65.3 | |  |
| Missing | | 0 | 0 | 4 | 0 | |  |
| **Month of symptom onset** | | | | | | | |
| November-December 2022 | | 19 | 70.4 | 50 | 40 | | **0.001** |
| January-February 2023 | | 8 | 29.6 | 34 | 27.2 | |  |
| March-May 2023 | | 0 | 0 | 41 | 32.8 | |  |
| **Time from symptom onset to swab (days)** | | | | | | | |
| 0 | | 0 | 0 | 2 | 1.6 | | 0.669 |
| 1 | | 2 | 7.4 | 14 | 11.2 | |  |
| 2 | | 7 | 25.9 | 21 | 16.8 | |  |
| 3 | | 6 | 22.2 | 33 | 26.4 | |  |
| 4 | | 5 | 18.5 | 20 | 16 | |  |
| 5 | | 4 | 14.8 | 10 | 8 | |  |
| 6 | | 0 | 0 | 8 | 6.4 | |  |
| 7 | | 3 | 11.1 | 17 | 13.6 | |  |
| **Influenza vaccine type** |  | | |  | |  | |
| Live Attenuated Influenza Vaccine | | 0 | 0 | 3 | 2.4 | | 0.393 |
| Other | | 0 | 0 | 3 | 2.4 | |  |
| Quadrivalent Influenza Vaccine | | 18 | 66.7 | 93 | 74.4 | |  |
| Unvaccinated | | 9 | 33.3 | 26 | 20.8 | |  |
| **Influenza (sub)type** |  | | |  | |  | |
| Control | | - | - | 125 | 100 | | - |
| Coinfection Influenza A (H3)/Influenza B | | 1 | 3.7 | - | - | |  |
| Influenza A - not subtyped | | 1 | 3.7 | - | - | |  |
| influenza A(H1N1)pdm09 | | 10 | 37 | - | - | |  |
| Influenza A(H3N2) | | 15 | 55.6 | - | - | |  |

**^†^***Mann Whitney U test*

**^‡^***Chi squared test or Fisher’s exact test when cell counts < 5*

**Table S4. Descriptive characteristics of cases with Influenza A(H1N1)pdm09 and controls, primary care influenza vaccine effectiveness study, Ireland 2022/2023**

|  | | | **Cases**  **(n = 104/728, 14.3%)** | | | **Controls**  **(n = 624/728, 85.7%)** | | |  |
| --- | --- | --- | --- | --- | --- | --- | --- | --- | --- |
| **Exposure** | | | **Median** | **IQR** | | **Median** | | **IQR** | **p-value^†^** |
| **Age (years)** | | | 42 | 27-54 | | 35 | | 11-55 | **0.030** |
| **Time from symptom onset to swab (days)** | | | 3 | 2-4 | | 3 | | 2-4 | **0.032** |
| **Exposure** | | | **n** | **%** | | **n** | | **%** | **p-value^‡^** |
| **Sex** | | |  |  | |  | |  |  |
| Female | | | 61 | 58.7 | | 370 | | 59.4 | 0.973 |
| Male | | | 43 | 41.3 | | 253 | | 40.6 |  |
| Missing | | | 0 | - | | 1 | | - |  |
| **Age group (years)** |  |  | | |  | |  | | |
| <2 | | | 2 | 1.9 | | 32 | | 5.1 | **<0.001** |
| 2-17 | | | 15 | 14.4 | | 174 | | 27.9 |  |
| 18-64 | | | 77 | 74 | | 324 | | 51.9 |  |
| 65+ | | | 10 | 9.6 | | 94 | | 15.1 |  |
| **Vaccination status** |  |  | | |  | |  | | |
| Unvaccinated | | | 83 | 79.8 | | 455 | | 73.7 | 0.233 |
| Vaccinated | | | 21 | 20.2 | | 162 | | 26.3 |  |
| Missing^§^ | | | 0 | - | | 7 | | - |  |
| **Chronic condition** |  |  | | |  | |  | | |
| No chronic condition | | | 78 | 76.5 | | 424 | | 70.1 | 0.231 |
| Chronic condition | | | 24 | 23.5 | | 181 | | 29.9 |  |
| Missing | | | 2 | - | | 19 | | - |  |
| **Month of symptom onset** | |  | | |  | |  | | |
| November-December 2022 | | | 82 | 78.8 | | 273 | | 43.8 | **<0.001** |
| January-February 2023 | | | 21 | 20.1 | | 280 | | 44.9 |  |
| March-May 2023 | | | 1 | 1.0 | | 71 | | 11.3 |  |
| **Time from symptom onset to swab (days)** | | | | |  | |  | | |
| 0 | | | 3 | 2.9 | | 12 | | 1.9 | 0.436 |
| 1 | | | 19 | 18.3 | | 78 | | 12.5 |  |
| 2 | | | 27 | 26 | | 138 | | 22.1 |  |
| 3 | | | 22 | 21.2 | | 160 | | 25.6 |  |
| 4 | | | 17 | 16.3 | | 101 | | 16.2 |  |
| 5 | | | 9 | 8.7 | | 54 | | 8.7 |  |
| 6 | | | 2 | 1.9 | | 30 | | 4.8 |  |
| 7 | | | 5 | 4.8 | | 51 | | 8.2 |  |
| **Influenza vaccine type** | | | | | | | | | |
| Live Attenuated Influenza Vaccine | | | 1 | 1 | | 22 | | 3.6 | 0.393 |
| Other | | | 1 | 1 | | 11 | | 1.8 |  |
| Quadrivalent Influenza Vaccine | | | 19 | 18.3 | | 129 | | 20.9 |  |
| Unvaccinated | | | 83 | 79.8 | | 455 | | 73.7 |  |
| Missing^§^ | | | 0 | - | | 7 | | - |  |
| **Pregnant** | | |  |  | |  | |  |  |
| No | | | 100 | 98 | | 610 | | 98.9 | 0.830 |
| Yes | | | 2 | 2 | | 7 | | 1.1 |  |
| Missing | | | 2 | - | | 7 | | - |  |

**^†^***Mann Whitney U test*

**^‡^***Chi squared test or Fisher’s exact test when cell counts < 5*

^§^*Missing as vaccinated 1-13 days before onset of symptoms*

**Table S5. Descriptive characteristics of cases with Influenza A(H3N2) and controls, primary care influenza vaccine effectiveness study, Ireland 2022/2023**

|  | | | **Case**  **(n =112/602, 18.6%)** | | | **Control**  **(n = 490/602, 81.4%)** | |  |
| --- | --- | --- | --- | --- | --- | --- | --- | --- |
| **Exposure** | | | **Median** | **IQR** | | **Median** | **IQR** | **p-value^†^** |
| **Age (years)** | | | 33 | 13-50 | | 36 | 11-56 | 0.751 |
| **Time from symptom onset to swab (days)** | | | 3 | 2-4 | | 3 | 2-4 | **0.005** |
| **Exposure** | | | **n** | **%** | | **n** | **%** | **p-value^‡^** |
| **Sex** | | |  |  | |  |  |  |
| Female | | | 65 | 58 | | 289 | 59 | 0.939 |
| Male | | | 47 | 42 | | 201 | 41 |  |
| **Age group (years)** |  |  | | |  | |  | |
| <2 | | | 1 | 0.9 | | 24 | 4.9 | 0.177 |
| 2-17 | | | 37 | 33 | | 132 | 26.9 |  |
| 18-64 | | | 58 | 51.8 | | 255 | 52 |  |
| 65+ | | | 16 | 14.3 | | 79 | 16.1 |  |
| **Vaccination status** |  |  | | |  | |  | |
| Unvaccinated | | | 94 | 84.7 | | 360 | 74.4 | **0.029** |
| Vaccinated | | | 17 | 15.3 | | 124 | 25.6 |  |
| Missing^§^ | | | 1 | - | | 6 | - |  |
| **Chronic condition** |  |  | | |  | |  | |
| No chronic condition | | | 88 | 79.3 | | 335 | 70.1 | 0.068 |
| Chronic condition | | | 23 | 20.7 | | 143 | 29.9 |  |
| Missing | | | 1 | - | | 12 | - |  |
| **Month of symptom onset** | | | | | | | | |
| November-December 2022 | | | 80 | 71.4 | | 266 | 54.3 | **0.001** |
| January-February 2023 | | | 32 | 28.6 | | 224 | 45.7 |  |
| March-May 2023 | | | 0 | 0 | | 0 | 0 |  |
| **Time from symptom onset to swab (days)** | | | | | | | | |
| 0 | | | 2 | 1.8 | | 10 | 2 | 0.063 |
| 1 | | | 19 | 17 | | 57 | 11.6 |  |
| 2 | | | 37 | 33 | | 107 | 21.8 |  |
| 3 | | | 23 | 20.5 | | 118 | 24.1 |  |
| 4 | | | 10 | 8.9 | | 84 | 17.1 |  |
| 5 | | | 11 | 9.8 | | 43 | 8.8 |  |
| 6 | | | 3 | 2.7 | | 25 | 5.1 |  |
| 7 | | | 7 | 6.2 | | 46 | 9.4 |  |
| **Influenza vaccine type** | | | | | | | | |
| Live Attenuated Influenza Vaccine | | | 3 | 2.7 | | 16 | 3.3 | 0.100 |
| Other | | | 0 | 0 | | 8 | 1.7 |  |
| Quadrivalent Influenza Vaccine | | | 14 | 12.5 | | 100 | 20.7 |  |
| Unvaccinated | | | 94 | 83.9 | | 360 | 74.4 |  |
| Missing^§^ | | | 1 | - | | 6 | - |  |
| **Pregnant** | | |  |  | |  |  |  |
| No | | | 111 | 100 | | 479 | 98.8 | 0.599 |
| Yes | | | 0 | 0 | | 6 | 1.2 |  |
| Missing | | | 1 | - | | 5 | - |  |

**^†^***Mann Whitney U test*

**^‡^***Chi squared test or Fisher’s exact test when cell counts < 5*

^§^*Missing as vaccinated 1-13 days before onset of symptoms*

**Table S6. Descriptive characteristics of cases with Influenza B and controls, primary care influenza vaccine effectiveness study, Ireland 2022/2023**

|  | | | **Cases**  **(n =89/886, 10.1%)** | | **Control**  **(n = 797/886, 89.9%)** | | |  |
| --- | --- | --- | --- | --- | --- | --- | --- | --- |
| **Exposure** | | | **Median** | **IQR** | **Median** | | **IQR** | **p-value^†^** |
| **Age (years)** | | | 21 | 14-36 | 35 | | 11-55 | **0.002** |
| **Time from symptom onset to swab (days)** | | | 3 | 2-4 | 3 | | 2-4 | 0.184 |
| **Exposure** | | | **n** | **%** | **n** | | **%** | **p-value^‡^** |
| **Sex** | | |  |  |  | |  |  |
| Female | | | 43 | 48.3 | 479 | | 60.2 | **0.041** |
| Male | | | 46 | 51.7 | 317 | | 39.8 |  |
| Missing | | | 0 | - | 1 | | - |  |
| **Age group (years)** | | | | | | | | |
| <2 | | | 0 | 0 | 42 | | 5.3 | **<0.001** |
| 2-17 | | | 35 | 39.3 | 218 | | 27.4 |  |
| 18-64 | | | 53 | 59.6 | 412 | | 51.7 |  |
| 65+ | | | 1 | 1.1 | 125 | | 15.7 |  |
| **Vaccination status** |  |  | | |  |  | | |
| Unvaccinated | | | 85 | 95.5 | 586 | | 74.2 | **<0.001** |
| Vaccinated | | | 4 | 4.5 | 204 | | 25.8 |  |
| Missing^§^ | | | 0 | - | 7 | | - |  |
| **Chronic condition** |  |  | | |  |  | | |
| No chronic condition | | | 72 | 84.7 | 545 | | 70.6 | **0.009** |
| Chronic condition | | | 13 | 15.3 | 227 | | 29.4 |  |
| Missing | | | 4 | - | 25 | | - |  |
| **Month of symptom onset** | | | | | | | | |
| November-December 2022 | | | 14 | 15.7 | 273 | | 34.3 | **0.001** |
| January-February 2023 | | | 44 | 49.4 | 280 | | 35.1 |  |
| March-May 2023 | | | 31 | 34.8 | 244 | | 30.6 |  |
| **Time from symptom onset to swab (days)** | | | | | | | | |
| 0 | | | 0 | 0 | 15 | | 1.9 | 0.508 |
| 1 | | | 17 | 19.1 | 104 | | 13 |  |
| 2 | | | 18 | 20.2 | 164 | | 20.6 |  |
| 3 | | | 25 | 28.1 | 206 | | 25.8 |  |
| 4 | | | 15 | 16.9 | 137 | | 17.2 |  |
| 5 | | | 8 | 9 | 68 | | 8.5 |  |
| 6 | | | 3 | 3.4 | 36 | | 4.5 |  |
| 7 | | | 3 | 3.4 | 67 | | 8.4 |  |
| **Influenza vaccine type** | | | | | | | | |
| Live Attenuated Influenza Vaccine | | | 1 | 1.1 | 26 | | 3.3 | **<0.001** |
| Other | | | 0 | 0 | 11 | | 1.4 |  |
| Quadrivalent Influenza Vaccine | | | 3 | 3.4 | 167 | | 21.1 |  |
| Unvaccinated | | | 85 | 95.5 | 586 | | 74.2 |  |
| Missing^§^ | | | 0 | - | 7 | | - |  |
| **Pregnant** | | |  |  |  | |  |  |
| No | | | 85 | 95.5 | 780 | | 99.1 | **0.019** |
| Yes | | | 4 | 4.5 | 7 | | 0.9 |  |
| Missing | | | 0 | - | 10 | | - |  |

**^†^***Mann Whitney U test*

**^‡^***Chi squared test or Fisher’s exact test when cell counts < 5*

^§^*Missing as vaccinated 1-13 days before onset of symptoms*

**Table S7. Descriptive characteristics of cases and controls age 2-17 years: Influenza A(H3N2), primary care influenza vaccine effectiveness study, Ireland 2022/2023**

|  | **Cases**  **(n =37/169, 21.9%)** | | **Controls**  **(n = 132/169, 78.1%)** | |  |
| --- | --- | --- | --- | --- | --- |
| **Exposure** | **Median** | **IQR** | **Median** | **IQR** | **p-value^†^** |
| **Age (years)** | 10 | 6-13 | 7 | 4-11 | **0.009** |
| **Time from symptom onset to swab (days)** | 3 | 2-4 | 3 | 2-4 | 0.051 |
| **Exposure** | **n** | **%** | **n** | **%** | **p-value^‡^** |
| Sex |  |  |  |  |  |
| Female | 19 | 51.4 | 70 | 53 | 1.000 |
| Male | 18 | 48.6 | 62 | 47 |  |
| **Vaccination status** | | | | | |
| Unvaccinated | 33 | 91.7 | 117 | 89.3 | 1.000 |
| Vaccinated | 3 | 8.3 | 14 | 10.7 |  |
| Missing^§^ | 1 | - | 1 | - |  |
| **Chronic condition** | | | | | |
| No chronic condition | 34 | 91.9 | 111 | 84.7 | 0.416 |
| Chronic condition | 3 | 8.1 | 20 | 15.3 |  |
| Missing | 0 | - | 1 | - |  |
| **Month of symptom onset** | | | | | |
| November-December 2022 | 26 | 70.3 | 76 | 57.6 | 0.187 |
| January-February 2023 | 11 | 29.7 | 56 | 42.4 |  |
| March-May 2023 | 0 | 0 | 0 | 0 |  |
| **Time from symptom onset to swab (days)** | | | | | |
| 0 | 2 | 5.4 | 1 | 0.8 | 0.241 |
| 1 | 7 | 18.9 | 18 | 13.6 |  |
| 2 | 14 | 37.8 | 35 | 26.5 |  |
| 3 | 5 | 13.5 | 33 | 25 |  |
| 4 | 4 | 10.8 | 25 | 18.9 |  |
| 5 | 3 | 8.1 | 9 | 6.8 |  |
| 6 | 0 | 0 | 4 | 3 |  |
| 7 | 2 | 5.4 | 7 | 5.3 |  |
| **Influenza vaccine type** | | | | | |
| Live Attenuated Influenza Vaccine | 3 | 8.3 | 13 | 9.9 | 1.000 |
| Quadrivalent Influenza Vaccine | 0 | 0 | 1 | 0.8 |  |
| Unvaccinated | 33 | 91.7 | 117 | 89.3 |  |
| Missing^§^ | 1 | - | 1 | - |  |

**^†^***Mann Whitney U test*

**^‡^***Chi squared test or Fisher’s exact test when cell counts < 5*

^§^*Missing as vaccinated 1-13 days before onset of symptoms*

**Table S8. Descriptive characteristics of cases and controls aged 18-64 years: Influenza A(H3N2), primary care influenza vaccine effectiveness study, Ireland 2022/2023**

|  | **Cases**  **(n =58/313, 18.5%)** | | **Controls**  **(n = 255/313, 81.5%)** | |  |
| --- | --- | --- | --- | --- | --- |
| **Exposure** | **Median** | **IQR** | **Median** | **IQR** | **p-value^†^** |
| **Age (years)** | 40 | 29-50 | 42 | 32-52 | 0.329 |
| **Time from symptom onset to swab (days)** | 3 | 2-4 | 3 | 2-4 | 0.071 |
| **Exposure** | **n** | **%** | **n** | **%** | **p-value^‡^** |
| **Sex** |  |  |  |  |  |
| Female | 38 | 65.5 | 163 | 63.9 | 0.939 |
| Male | 20 | 34.5 | 92 | 36.1 |  |
| **Vaccination status** | |  |  |  |  |
| Unvaccinated | 54 | 93.1 | 199 | 79.6 | **0.013** |
| Vaccinated | 4 | 6.9 | 51 | 20.4 |  |
| Missing^§^ | 0 | 0 | 5 | 0 |  |
| **Chronic condition** | |  |  |  |  |
| No chronic condition | 48 | 82.5 | 175 | 70.6 | 0.072 |
| Chronic condition | 10 | 17.5 | 73 | 29.4 |  |
| Missing | 0 | 0 | 7 | 0 |  |
| **Month of symptom onset** | |  |  |  |  |
| November-December 2022 | 43 | 74.1 | 127 | 49.8 | **0.001** |
| January-February 2023 | 15 | 25.9 | 128 | 50.2 |  |
| March-May 2023 | 0 | 0 | 0 | 0 |  |
| **Time from symptom onset to swab (days)** | | |  |  |  |
| 0 | 0 | 0 | 6 | 2.4 | 0.255 |
| 1 | 10 | 17.2 | 29 | 11.4 |  |
| 2 | 18 | 31 | 54 | 21.2 |  |
| 3 | 13 | 22.4 | 56 | 22 |  |
| 4 | 4 | 6.9 | 42 | 16.5 |  |
| 5 | 7 | 12.1 | 26 | 10.2 |  |
| 6 | 3 | 5.2 | 14 | 5.5 |  |
| 7 | 3 | 5.2 | 28 | 11 |  |
| **Influenza vaccine type** | |  |  |  |  |
| Live Attenuated Influenza Vaccine | 0 | 0 | 1 | 0.4 | 0.090 |
| Other | 0 | 0 | 5 | 2.0 |  |
| Quadrivalent Influenza Vaccine | 4 | 6.9 | 45 | 18.0 |  |
| Unvaccinated | 54 | 93.1 | 199 | 79.6 |  |
| Missing^§^ | 0 | - | 5 | - |  |

**^†^***Mann Whitney U test*

**^‡^***Chi squared test or Fisher’s exact test when cell counts < 5*

^§^*Missing as vaccinated 1-13 days before onset of symptoms*

**Table S9. Descriptive characteristics of cases and controls aged 65+ years: Influenza A(H3N2) , primary care influenza vaccine effectiveness study, Ireland 2022/2023**

|  | **Cases**  **(n = 16/95, 16.8%)** | | **Controls**  **(n = 79/95, 83.2%)** | |  |
| --- | --- | --- | --- | --- | --- |
| **Exposure** | **Median** | **IQR** | **Median** | **IQR** | **p-value^†^** |
| **Age (years)** | 71.5 | 67-74 | 73 | 69-80 | 0.124 |
| **Time from symptom onset to swab (days)** | 3 | 2-4 | 3 | 2-4 | 0.310 |
| **Exposure** | **n** | **%** | **n** | **%** | **p-value^‡^** |
| Sex |  |  |  |  |  |
| Female | 7 | 43.8 | 46 | 58.2 | 0.431 |
| Male | 9 | 56.2 | 33 | 41.8 |  |
| **Vaccination status** | |  |  |  |  |
| Unvaccinated | 6 | 37.5 | 20 | 25.3 | 0.362 |
| Vaccinated | 10 | 62.5 | 59 | 74.7 |  |
| **Chronic condition** | |  |  |  |  |
| No chronic condition | 6 | 37.5 | 26 | 34.7 | 1.000 |
| Chronic condition | 10 | 62.5 | 49 | 65.3 |  |
| Missing | 0 | 0 | 4 | 0 |  |
| **Month of symptom onset** | |  |  |  |  |
| November-December 2022 | 10 | 62.5 | 49 | 62 | 1.000 |
| January-February 2023 | 6 | 37.5 | 30 | 38 |  |
| March-May 2023 | 0 | 0 | 0 | 0 |  |
| **Time from symptom onset to swab (days)** | | |  |  |  |
| 0 | 0 | 0 | 1 | 1.3 | 0.942 |
| 1 | 2 | 12.5 | 8 | 10.1 |  |
| 2 | 5 | 31.2 | 15 | 19 |  |
| 3 | 4 | 25 | 19 | 24.1 |  |
| 4 | 2 | 12.5 | 12 | 15.2 |  |
| 5 | 1 | 6.2 | 7 | 8.9 |  |
| 6 | 0 | 0 | 7 | 8.9 |  |
| 7 | 2 | 12.5 | 10 | 12.7 |  |
| **Influenza vaccine type** | |  |  |  |  |
| Live Attenuated Influenza Vaccine | 0 | 0 | 2 | 2.5 | 0.822 |
| Other | 0 | 0 | 3 | 3.8 |  |
| Quadrivalent Influenza Vaccine | 10 | 62.5 | 54 | 68.4 |  |
| Unvaccinated | 6 | 37.5 | 20 | 25.3 |  |

**^†^***Mann Whitney U test*

**^‡^***Chi squared test or Fisher’s exact test when cell counts < 5*

**Table S10. Descriptive characteristics of cases and controls aged 2-17 years: Influenza A(H1N1)pdm09, primary care influenza vaccine effectiveness study, Ireland 2022/2023**

|  | **Cases**  **(n =15/189, 7.9%)** | | **Controls**  **(n =174/189, 92.1%)** | |  |
| --- | --- | --- | --- | --- | --- |
| **Exposure** | **Median** | **IQR** | **Median** | **IQR** | **p-value^†^** |
| **Age (years)** | 14 | 9-15 | 7 | 4-11 | 0.008 |
| **Time from symptom onset to swab (days)** | 3 | 2-4 | 3 | 2-4 | 0.565 |
| **Exposure** | **n** | **%** | **n** | **%** | **p-value^‡^** |
| **Sex** |  |  |  |  |  |
| Female | 6 | 40 | 91 | 52.6 | 0.504 |
| Male | 9 | 60 | 82 | 47.4 |  |
| Missing | 0 | - | 1 | - |  |
| **Vaccination status** | |  |  |  |  |
| Unvaccinated | 14 | 93.3 | 151 | 87.3 | 0.699 |
| Vaccinated | 1 | 6.7 | 22 | 12.7 |  |
| Missing^§^ | 0 | - | 1 | - |  |
| **Chronic condition** | |  |  |  |  |
| No chronic condition | 14 | 93.3 | 143 | 84.1 | 0.475 |
| Chronic condition | 1 | 6.7 | 27 | 15.9 |  |
| Missing | 0 | - | 4 | - |  |
| **Month of symptom onset** | |  |  |  |  |
| November-December 2022 | 13 | 86.7 | 79 | 45.4 | 0.010 |
| January-February 2023 | 2 | 13.3 | 71 | 40.8 |  |
| March-May 2023 | 0 | 0 | 24 | 13.8 |  |
| **Time from symptom onset to swab (days)** | | |  |  |  |
| 0 | 0 | 0 | 2 | 1.1 | 0.364 |
| 1 | 5 | 33.3 | 25 | 14.4 |  |
| 2 | 3 | 20 | 44 | 25.3 |  |
| 3 | 1 | 6.7 | 45 | 25.9 |  |
| 4 | 3 | 20 | 30 | 17.2 |  |
| 5 | 1 | 6.7 | 12 | 6.9 |  |
| 6 | 1 | 6.7 | 8 | 4.6 |  |
| 7 | 1 | 6.7 | 8 | 4.6 |  |
| **Influenza vaccine type** | |  |  |  |  |
| Live Attenuated Intranasal Vaccine | 1 | 6.7 | 19 | 11.0 | 1.000 |
| Quadrivalent Influenza Vaccine | 0 | 0 | 3 | 1.7 |  |
| Unvaccinated | 14 | 93.3 | 151 | 87.3 |  |
| Missing^§^ | 0 | - | 1 | - |  |

**^†^***Mann Whitney U test*

**^‡^***Chi squared test or Fisher’s exact test when cell counts < 5*

^§^*Missing as vaccinated 1-13 days before onset of symptoms*

**Table S11. Descriptive characteristics of cases and controls aged 18-64 years: Influenza A(H1N1)pdm09, primary care influenza vaccine effectiveness study, Ireland 2022/2023**

|  | **Cases**  **(n =77/401, 19.2%)** | | **Controls**  **(n = 324/401, 80.8%)** | |  |
| --- | --- | --- | --- | --- | --- |
| **Exposure** | **Median** | **IQR** | **Median** | **IQR** | **p-value^†^** |
| **Age (years)** | 43 | 35-54 | 42 | 32-52 | 0.515 |
| **Time from symptom onset to swab (days)** | 3 | 2-4 | 3 | 2-4 | **0.013** |
| **Exposure** | **n** | **%** | **n** | **%** | **p-value^‡^** |
| Sex |  |  |  |  |  |
| Female | 47 | 61 | 211 | 65.1 | 0.589 |
| Male | 30 | 39 | 113 | 34.9 |  |
| **Vaccination status** | |  |  |  |  |
| Unvaccinated | 64 | 83.1 | 251 | 78.9 | 0.527 |
| Vaccinated | 13 | 16.9 | 67 | 21.1 |  |
| Missing^§^ | 0 | - | 6 | - |  |
| **Chronic condition** | |  |  |  |  |
| No chronic condition | 58 | 77.3 | 225 | 71.7 | 0.387 |
| Chronic condition | 17 | 22.7 | 89 | 28.3 |  |
| Missing | 0 | - | 10 | - |  |
| **Month of symptom onset** | |  |  |  |  |
| November-December 2022 | 59 | 76.6 | 130 | 40.1 | **<0.001** |
| January-February 2023 | 17 | 22.1 | 161 | 49.7 |  |
| March-May 2023 | 1 | 1.3 | 33 | 10.2 |  |
| **Time from symptom onset to swab (days)** | | |  |  |  |
| 0 | 3 | 3.9 | 6 | 1.9 | 0.444 |
| 1 | 13 | 16.9 | 39 | 12 |  |
| 2 | 21 | 27.3 | 71 | 21.9 |  |
| 3 | 19 | 24.7 | 79 | 24.4 |  |
| 4 | 11 | 14.3 | 51 | 15.7 |  |
| 5 | 5 | 6.5 | 32 | 9.9 |  |
| 6 | 1 | 1.3 | 15 | 4.6 |  |
| 7 | 4 | 5.2 | 31 | 9.6 |  |
| **Influenza vaccine type** | |  |  |  |  |
| Live Attenuated Influenza Vaccine | 0 | 0 | 1 | 0.3 | 0.784 |
| Other | 1 | 1.3 | 8 | 2.5 |  |
| Quadrivalent Influenza Vaccine | 12 | 15.6 | 58 | 18.2 |  |
| Unvaccinated | 64 | 83.1 | 251 | 78.9 |  |
| Missing^§^ | 0 | - | 6 | - |  |

**^†^***Mann Whitney U test*

**^‡^***Chi squared test or Fisher’s exact test when cell counts < 5*

^§^*Missing as vaccinated 1-13 days before onset of symptoms*

**Table S12. Descriptive characteristics of cases and controls aged 65+ years: Influenza A(H1N1)pdm09, primary care influenza vaccine effectiveness study, Ireland 2022/2023**

|  | **Cases**  **(n =10/104, 9.6%)** | | **Controls**  **(n = 94/104, 90.4%)** | |  |
| --- | --- | --- | --- | --- | --- |
| **Exposure** | **Median** | **IQR** | **Median** | **IQR** | **p-value^†^** |
| **Age (years)** | 69 | 66-70 | 74 | 70-80 | **0.002** |
| **Time from symptom onset to swab (days)** | 3 | 2-4 | 3 | 2-4 | 0.527 |
| **Exposure** | **n** | **%** | **n** | **%** | **p-value^‡^** |
| **Sex** |  |  |  |  |  |
| Female | 6 | 60 | 56 | 59.6 | 1.000 |
| Male | 4 | 40 | 38 | 40.4 |  |
| **Vaccination status** | |  |  |  |  |
| Unvaccinated | 3 | 30 | 21 | 22.3 | 0.693 |
| Vaccinated | 7 | 70 | 73 | 77.7 |  |
| **Chronic condition** | |  |  |  |  |
| No chronic condition | 4 | 40 | 29 | 32.2 | 0.887 |
| Chronic condition | 6 | 60 | 61 | 67.8 |  |
| Missing | 0 | 0 | 4 | 0 |  |
| **Month of symptom onset** | |  |  |  |  |
| November-December 2022 | 8 | 80 | 50 | 53.2 | 0.356 |
| January-February 2023 | 2 | 20 | 34 | 36.2 |  |
| March-May 2023 | 0 | 0 | 10 | 10.6 |  |
| **Time from symptom onset to swab (days)** | | |  |  |  |
| 0 | 0 | 0 | 1 | 1.1 | 0.377 |
| 1 | 0 | 0 | 11 | 11.7 |  |
| 2 | 2 | 20 | 19 | 20.2 |  |
| 3 | 2 | 20 | 23 | 24.5 |  |
| 4 | 3 | 30 | 14 | 14.9 |  |
| 5 | 3 | 30 | 8 | 8.5 |  |
| 6 | 0 | 0 | 7 | 7.4 |  |
| 7 | 0 | 0 | 11 | 11.7 |  |
| **Influenza vaccine type** | |  |  |  |  |
| Live Attenuated Influenza Vaccine | 0 | 0 | 2 | 2.1 | 0.822 |
| Other | 0 | 0 | 3 | 3.2 |  |
| Quadrivalent Influenza Vaccine | 7 | 70 | 68 | 72.3 |  |
| Unvaccinated | 3 | 30 | 21 | 22.3 |  |

**^†^***Mann Whitney U test*

**^‡^***Chi squared test or Fisher’s exact test when cell counts < 5*

**Table S13. Descriptive characteristics of cases and controls aged 2-17years: Influenza B, primary care influenza vaccine effectiveness study, Ireland 2022/2023**

|  | **Cases**  **(n =35/253, 13.8%)** | | **Controls**  **(n = 218/253, 86.2%)** | |  |
| --- | --- | --- | --- | --- | --- |
| **Exposure** | **Median** | **IQR** | **Median** | **IQR** | **p-value^†^** |
| **Age (years)** | 12 | 8-14 | 7 | 4-12 | **0.001** |
| **Time from symptom onset to swab (days)** | 3 | 2-4 | 3 | 2-4 | **0.049** |
| **Exposure** | **n** | **%** | **n** | **%** | **p-value^‡^** |
| **Sex** |  |  |  |  |  |
| Female | 18 | 51.4 | 113 | 52.1 | 1.000 |
| Male | 17 | 48.6 | 104 | 47.9 |  |
| Missing | 0 | 0 | 1 | 0 |  |
| **Vaccination status** | |  |  |  |  |
| Unvaccinated | 34 | 97.1 | 193 | 88.9 | 0.219 |
| Vaccinated | 1 | 2.9 | 24 | 11.1 |  |
| Missing^§^ | 0 | 0 | 1 | 0 |  |
| **Chronic condition** | |  |  |  |  |
| No chronic condition | 32 | 91.4 | 178 | 84 | 0.315 |
| Chronic condition | 3 | 8.6 | 34 | 16 |  |
| Missing | 0 | 0 | 6 | 0 |  |
| **Month of symptom onset** | |  |  |  |  |
| November-December 2022 | 6 | 17.1 | 79 | 36.2 | **0.014** |
| January-February 2023 | 20 | 57.1 | 71 | 32.6 |  |
| March-May 2023 | 9 | 25.7 | 68 | 31.2 |  |
| **Time from symptom onset to swab (days)** | | |  |  |  |
| 0 | 0 | 0 | 3 | 1.4 | 0.585 |
| 1 | 10 | 28.6 | 35 | 16.1 |  |
| 2 | 11 | 31.4 | 56 | 25.7 |  |
| 3 | 7 | 20 | 53 | 24.3 |  |
| 4 | 4 | 11.4 | 38 | 17.4 |  |
| 5 | 1 | 2.9 | 15 | 6.9 |  |
| 6 | 2 | 5.7 | 10 | 4.6 |  |
| 7 | 0 | 0 | 8 | 3.7 |  |
| **Influenza vaccine type** | |  |  |  |  |
| Live Attenuated Influenza Vaccine | 1 | 2.9 | 21 | 9.7 | 0.495 |
| Quadrivalent Influenza Vaccine | 0 | 0 | 3 | 1.4 |  |
| Unvaccinated | 34 | 97.1 | 193 | 88.9 |  |
| Missing^§^ | 0 | - | 1 | - |  |

**^†^***Mann Whitney U test*

**^‡^***Chi squared test or Fisher’s exact test when cell counts < 5*

^§^*Missing as vaccinated 1-13 days before onset of symptoms*

**Table S14. Descriptive characteristics of cases and controls aged 18-64 years: Influenza B, primary care influenza vaccine effectiveness study, Ireland 2022/2023**

|  | **Cases**  **(n =53/465, 11.4%)** | | **Controls**  **(n = 412/465, 88.6%)** | |  |
| --- | --- | --- | --- | --- | --- |
| **Exposure** | **Median** | **IQR** | **Median** | **IQR** | **p-value^†^** |
| **Age (years)** | 34 | 24-41 | 41 | 30-51 | **<0.001** |
| **Time from symptom onset to swab (days)** | 3 | 2-4 | 3 | 2-4 | 0.835 |
| **Exposure** | **n** | **%** | **n** | **%** | **p-value^‡^** |
| **Sex** |  |  |  |  |  |
| Female | 24 | 45.3 | 272 | 66 | **0.005** |
| Male | 29 | 54.7 | 140 | 34 |  |
| **Vaccination status** | |  |  |  |  |
| Unvaccinated | 51 | 96.2 | 325 | 80 | **0.002** |
| Vaccinated | 2 | 3.8 | 81 | 20 |  |
| Missing^§^ | 0 | - | 6 | - |  |
| **Chronic condition** | |  |  |  |  |
| No chronic condition | 39 | 79.6 | 289 | 72.6 | 0.392 |
| Chronic condition | 10 | 20.4 | 109 | 27.4 |  |
| Missing | 0 | - | 14 | - |  |
| **Month of symptom onset** | |  |  |  |  |
| November-December 2022 | 7 | 13.2 | 130 | 31.6 | **0.012** |
| January-February 2023 | 24 | 45.3 | 161 | 39.1 |  |
| March-May 2023 | 22 | 41.5 | 121 | 29.4 |  |
| **Time from symptom onset to swab (days)** | | |  |  |  |
| 0 | 0 | 0 | 7 | 1.7 | 0.750 |
| 1 | 7 | 13.2 | 50 | 12.1 |  |
| 2 | 7 | 13.2 | 81 | 19.7 |  |
| 3 | 17 | 32.1 | 105 | 25.5 |  |
| 4 | 11 | 20.8 | 72 | 17.5 |  |
| 5 | 7 | 13.2 | 41 | 10 |  |
| 6 | 1 | 1.9 | 18 | 4.4 |  |
| 7 | 3 | 5.7 | 38 | 9.2 |  |
| **Influenza vaccine type** | |  |  |  |  |
| Live Attenuated Influenza Vaccine | 0 | 0 | 2 | 0.5 | **0.032** |
| Other | 0 | 0 | 8 | 2 |  |
| Quadrivalent Influenza Vaccine | 2 | 3.8 | 71 | 17.5 |  |
| Unvaccinated | 51 | 96.2 | 325 | 80 |  |
| Missing^§^ | 0 | - | 6 | - |  |

**^†^***Mann Whitney U test*

**^‡^***Chi squared test or Fisher’s exact test when cell counts < 5*

^§^*Missing as vaccinated 1-13 days before onset of symptoms*

**Table S15. Descriptive characteristics of cases and controls aged 65+ years: Influenza B, primary care influenza vaccine effectiveness study, Ireland 2022/2023**

|  | **Cases**  **(n =1/126, 0.8%)** | | **Controls**  **(n=125/126/99.2%)** | |  |
| --- | --- | --- | --- | --- | --- |
| **Exposure** | **Median** | **IQR** | **Median** | **IQR** | **p-value^†^** |
| **Age (years)** | 68 | 68-68 | 73 | 69-80 | 0.259 |
| **Time from symptom onset to swab (days)** | 3 | 2-4 | 3 | 2-4 | 0.812 |
| **Exposure** | **n** | **%** | **n** | **%** | **p-value^‡^** |
| **Sex** |  |  |  |  |  |
| Female | 1 | 100 | 75 | 60 | 1.000 |
| Male | 0 | 0 | 50 | 40 |  |
| **Vaccination status** | |  |  |  |  |
| Unvaccinated | 0 | 0 | 26 | 20.8 | 1.000 |
| Vaccinated | 1 | 100 | 99 | 79.2 |  |
| **Chronic condition** | |  |  |  |  |
| No chronic condition | 1 | 100 | 42 | 34.7 | 0.756 |
| Chronic condition | 0 | 0 | 79 | 65.3 |  |
| Missing | 0 | - | 4 | - |  |
| **Month of symptom onset** | |  |  |  |  |
| November-December 2022 | 1 | 100 | 50 | 40 | 1.000 |
| January-February 2023 | 0 | 0 | 34 | 27.2 |  |
| March-May 2023 | 0 | 0 | 41 | 32.8 |  |
| **Time from symptom onset to swab (days)** | | |  |  |  |
| 0 | 0 | 0 | 2 | 1.6 | 1.000 |
| 1 | 0 | 0 | 14 | 11.2 |  |
| 2 | 0 | 0 | 21 | 16.8 |  |
| 3 | 1 | 100 | 33 | 26.4 |  |
| 4 | 0 | 0 | 20 | 16 |  |
| 5 | 0 | 0 | 10 | 8 |  |
| 6 | 0 | 0 | 8 | 6.4 |  |
| 7 | 0 | 0 | 17 | 13.6 |  |
| **Influenza vaccine type** | |  |  |  |  |
| Live Attenuated Influenza Vaccine | 0 | 0 | 3 | 2.4 | 1.000 |
| Other | 0 | 0 | 3 | 2.4 |  |
| Quadrivalent Influenza Vaccine | 1 | 100 | 93 | 74.4 |  |
| Unvaccinated | 0 | 0 | 26 | 20.8 |  |

**^†^***Mann Whitney U test*

**^‡^***Chi squared test or Fisher’s exact test when cell counts < 5*

**Table S16. Descriptive characteristics of cases and controls aged 2-17 years, primary care influenza vaccine effectiveness study, Ireland 2023/2024**

| **Exposure** | **Cases (n = 129/527, 24.5%)** | | **Control (398/527, 75.5%)** | |  |
| --- | --- | --- | --- | --- | --- |
|  | **Median** | **IQR** | **Median** | **IQR** | **p-value^†^** |
| **Age (years)** | 10 | 6-14 | 9 | 5-13 | **0.014** |
| **Symptom onset to swab (days)** | 3 | 2-4 | 3 | 2-5 | 0.059 |
| **Exposure** | **n** | **%** | **n** | **%** | **p-value^‡^** |
| **Sex** |  |  |  |  |  |
| Female | 61 | 47.3 | 199 | 50 | 0.664 |
| Male | 68 | 52.7 | 199 | 50 |  |
| **Vaccination status** | |  |  |  |  |
| Unvaccinated | 119 | 93 | 342 | 85.9 | 0.051 |
| Vaccinated | 9 | 7 | 56 | 14.1 |  |
| Missing^§^ | 1 | - | 0 | - |  |
| **Chronic condition** | |  |  |  |  |
| No chronic condition | 108 | 86.4 | 321 | 84.9 | 0.796 |
| Chronic condition | 17 | 13.6 | 57 | 15.1 |  |
| Missing | 4 | - | 20 | - |  |
| **Month of symptom onset** | |  |  |  |  |
| October-December 2023 | 34 | 26.4 | 156 | 39.2 | **<0.001** |
| January-February 2024 | 82 | 63.6 | 143 | 35.9 |  |
| March-April 2024 | 13 | 10.1 | 99 | 24.9 |  |
| **Time from symptom onset to swab (days)** | | |  |  |  |
| 0 | 4 | 3.1 | 12 | 3 | 0.087 |
| 1 | 13 | 10.1 | 43 | 10.8 |  |
| 2 | 47 | 36.4 | 98 | 24.6 |  |
| 3 | 26 | 20.2 | 96 | 24.1 |  |
| 4 | 16 | 12.4 | 61 | 15.3 |  |
| 5 | 14 | 10.9 | 29 | 7.3 |  |
| 6 | 4 | 3.1 | 23 | 5.8 |  |
| 7 | 5 | 3.9 | 36 | 9 |  |
| **Influenza vaccine type** | |  |  |  |  |
| Live attenuated influenza vaccine | 8 | 6.2 | 52 | 13.1 | 0.186 |
| Quadrivalent influenza vaccine | 1 | 0.8 | 3 | 0.8 |  |
| Other (not specified) | 0 | 0 | 1 | 0.3 |  |
| Unvaccinated | 119 | 93 | 342 | 85.9 |  |
| Missing^§^ | 1 | - | 0 | - |  |
| **Influenza (sub)type** | |  |  |  |  |
| Control | - | - | 398 | 100.0 | - |
| Influenza A - not subtyped | 3 | 2.3 | - | - |  |
| Influenza A(H1N1)pdm09 | 25 | 19.4 | - | - |  |
| Influenza A(H3N2) | 78 | 60.5 | - | - |  |
| Influenza B | 23 | 17.8 | - | - |  |

**^†^***Mann Whitney U test*

**^‡^***Chi squared test or Fisher’s exact test when cell counts < 5*

^§^*Missing as vaccinated 1-13 days before onset of symptoms*

**Table S17. Descriptive characteristics of cases and controls aged 18-64 years, primary care influenza vaccine effectiveness study, Ireland 2023/2024**

| **Exposure** | **Case**  **(435/1,656, 26.3%)** | | **Control**  **(1,221/1,656, 73.7%)** | |  |
| --- | --- | --- | --- | --- | --- |
|  | **Median** | **IQR** | **Median** | **IQR** | **p-value^†^** |
| **Age (years)** | 39 | 29-49 | 42 | 32-52 | **<0.001** |
| **Symptom onset to swab (days)** | 3 | 2-4 | 3 | 2-5 | **<0.001** |
| **Exposure** | **n** | **%** | **n** | **%** | **p-value^‡^** |
| **Sex** |  |  |  |  |  |
| Female | 270 | 62.2 | 821 | 67.2 | 0.066 |
| Male | 164 | 37.8 | 400 | 32.8 |  |
| Missing | 1 | - | 0 | - |  |
| **Vaccination status** | |  |  |  |  |
| Unvaccinated | 369 | 85.2 | 945 | 78.8 | **0.005** |
| Vaccinated | 64 | 14.8 | 254 | 21.2 |  |
| Missing^§^ | 2 | - | 22 | - |  |
| **Chronic condition** | |  |  |  |  |
| No chronic condition | 304 | 78.1 | 791 | 71.2 | **0.010** |
| Chronic condition | 85 | 21.9 | 320 | 28.8 |  |
| Missing | 46 | - | 110 | - |  |
| **Month of symptom onset** | |  |  |  |  |
| October-December 2023 | 106 | 24.4 | 438 | 35.9 | **<0.001** |
| January-February 2024 | 271 | 62.3 | 480 | 39.3 |  |
| March-May 2024 | 58 | 13.3 | 303 | 24.8 |  |
| **Time from symptom onset to swab (days)** | | |  |  |  |
| 0 | 5 | 1.1 | 24 | 2 | **<0.001** |
| 1 | 60 | 13.8 | 163 | 13.4 |  |
| 2 | 125 | 28.7 | 259 | 21.2 |  |
| 3 | 106 | 24.4 | 245 | 20.1 |  |
| 4 | 65 | 14.9 | 182 | 14.9 |  |
| 5 | 29 | 6.7 | 112 | 9.2 |  |
| 6 | 23 | 5.3 | 69 | 5.7 |  |
| 7 | 22 | 5.1 | 167 | 13.7 |  |
| **Influenza vaccine type** | |  |  |  |  |
| Live attenuated influenza vaccine | 2 | 0.5 | 10 | 0.8 | **0.015** |
| Quadrivalent influenza vaccine | 62 | 14.3 | 244 | 20.3 |  |
| Unvaccinated | 369 | 85.2 | 945 | 78.8 |  |
| Missing^§^ | 2 | - | 22 | - |  |
| **Pregnant** |  |  |  |  |  |
| No | 403 | 97.6 | 1152 | 97.4 | 0.968 |
| Yes | 10 | 2.4 | 31 | 2.6 |  |
| Missing | 22 | - | 38 | - |  |
| **Influenza (sub)type** | |  |  |  |  |
| Control | - | - | 1221 | 100 | - |
| Coinfection Influenza A (H3N1)/Influenza B | 1 | 0.2 | - | - |  |
| Influenza A - not subtyped | 29 | 6.7 | - | - |  |
| Influenza A(H1N1)pdm09 | 113 | 26 | - | - |  |
| Influenza A(H3N2) | 218 | 50.1 | - | - |  |
| Influenza B/lineage unknown | 74 | 17 | - | - |  |

**^†^***Mann Whitney U test*

**^‡^***Chi squared test or Fisher’s exact test when cell counts < 5*

^§^*Missing as vaccinated 1-13 days before onset of symptoms*

**Table S18. Descriptive characteristics of cases and controls aged 65 years and older, primary care influenza vaccine effectiveness study, Ireland 2023/2024**

| **Exposure** | **Case (49/366, 13.4%)** | | **Control (317/366, 86.6%)** | |  |
| --- | --- | --- | --- | --- | --- |
|  | **Median** | **IQR** | **Median** | **IQR** | **p-value^†^** |
| **Age (years)** | 72 | 68-77 | 72 | 69-77 | 0.374 |
| **Symptom onset to swab (days)** | 3 | 2-4 | 3 | 2-5 | 0.059 |
| **Exposure** | **n** | **%** | **n** | **%** | **p-value^‡^** |
| **Sex** |  |  |  |  |  |
| Female | 28 | 57.1 | 201 | 63.4 | 0.494 |
| Male | 21 | 42.9 | 116 | 36.6 |  |
| **Vaccination status** | |  |  |  |  |
| Unvaccinated | 15 | 31.9 | 104 | 35.3 | 1.000 |
| Vaccinated | 32 | 68.1 | 191 | 64.7 |  |
| Missing^§^ | 2 | - | 22 | - |  |
| **Chronic condition** | |  |  |  |  |
| No chronic condition | 18 | 39.1 | 104 | 35.3 | 0.730 |
| Chronic condition | 28 | 60.9 | 191 | 64.7 |  |
| Missing | 3 | - | 22 | - |  |
| **Month of symptom onset** | |  |  |  |  |
| October-December 2023 | 14 | 28.6 | 116 | 36.6 | **<0.001** |
| January-February 2024 | 32 | 65.3 | 113 | 35.6 |  |
| March-May 2024 | 3 | 6.1 | 88 | 27.8 |  |
| **Time from symptom onset to swab (days)** | | |  |  |  |
| 0 | 0 | 0 | 3 | 0.9 | 0.147 |
| 1 | 5 | 10.2 | 27 | 8.5 |  |
| 2 | 17 | 34.7 | 57 | 18 |  |
| 3 | 8 | 16.3 | 84 | 26.5 |  |
| 4 | 10 | 20.4 | 58 | 18.3 |  |
| 5 | 4 | 8.2 | 30 | 9.5 |  |
| 6 | 3 | 6.1 | 20 | 6.3 |  |
| 7 | 2 | 4.1 | 38 | 12 |  |
| **Influenza vaccine type** | |  |  |  |  |
| Live attenuated influenza vaccine | 0 | 0 | 3 | 1.0 | 0.790 |
| Quadrivalent influenza vaccine | 32 | 68.1 | 206 | 66.7 |  |
| Unvaccinated | 15 | 31.9 | 100 | 32.4 |  |
| Missing^§^ | 2 | - | 8 | - |  |
| **Influenza (sub)type** | |  |  |  |  |
| Control | - | - | 317 | 100 | - |
| Influenza A - not subtyped | 4 | 8.2 | - | - |  |
| Influenza A(H1N1)pdm09 | 14 | 28.6 | - | - |  |
| Influenza A(H3N2) | 31 | 63.3 | - | - |  |

**^†^***Mann Whitney U test*

**^‡^***Chi squared test or Fisher’s exact test when cell counts < 5*

^§^*Missing as vaccinated 1-13 days before onset of symptoms*

**Table S19. Descriptive characteristics of cases with influenza A(H1N1)pdm09 and controls, primary care influenza vaccine effectiveness study, Ireland 2023/2024**

| **Exposure** | **Case**  **(158/2,167, 7.3%)** | | **Control**  **(2,009/2,167, 92.7%)** | |  |
| --- | --- | --- | --- | --- | --- |
|  | **Median** | **IQR** | **Median** | **IQR** | **p-value^†^** |
| **Age (years)** | 41 | 29-52 | 39 | 19-57 | 0.836 |
| **Symptom onset to swab (days)** | 3 | 2-4 | 3 | 2-5 | **0.005** |
| **Exposure** | **n** | **%** | **n** | **%** | **p-value^‡^** |
| **Sex** |  |  |  |  |  |
| Female | 105 | 66.5 | 1251 | 62.3 | 0.336 |
| Male | 53 | 33.5 | 758 | 37.7 |  |
| **Age group (years)** |  |  |  |  |  |
| <2 | 5 | 3.2 | 79 | 3.9 | **0.028** |
| 2-17 | 25 | 15.8 | 397 | 19.8 |  |
| 18-64 | 114 | 72.2 | 1217 | 60.6 |  |
| 65+ | 14 | 8.9 | 316 | 15.7 |  |
| **Vaccination status** | |  |  |  |  |
| Unvaccinated | 125 | 79.6 | 1462 | 73.9 | 0.136 |
| Vaccinated | 32 | 20.4 | 517 | 26.1 |  |
| Missing^§^ | 1 | - | 30 | - |  |
| **Chronic condition** | |  |  |  |  |
| No chronic condition | 125 | 79.6 | 1462 | 73.9 | **0.006** |
| Chronic condition | 32 | 20.4 | 517 | 26.1 |  |
| Missing | 1 | - | 30 | - |  |
| **Month of symptom onset** | |  |  |  |  |
| October-December 2023 | 37 | 23.4 | 749 | 37.3 | **<0.001** |
| January-February 2024 | 110 | 69.6 | 757 | 37.7 |  |
| March-May 2024 | 11 | 7.0 | 503 | 25.0 |  |
| **Time from symptom onset to swab (days)** | | |  |  |  |
| 0 | 2 | 1.3 | 40 | 2.0 | **0.018** |
| 1 | 18 | 11.4 | 242 | 12.0 |  |
| 2 | 49 | 31 | 428 | 21.3 |  |
| 3 | 41 | 25.9 | 439 | 21.9 |  |
| 4 | 23 | 14.6 | 318 | 15.8 |  |
| 5 | 12 | 7.6 | 178 | 8.9 |  |
| 6 | 6 | 3.8 | 120 | 6.0 |  |
| 7 | 7 | 4.4 | 244 | 12.1 |  |
| **Influenza vaccine type** | |  |  |  |  |
| Live attenuated influenza vaccine | 4 | 2.5 | 64 | 3.2 | 0.464 |
| Other | 0 | 0 | 1 | 0.1 |  |
| Quadrivalent influenza vaccine | 28 | 17.8 | 452 | 22.8 |  |
| Unvaccinated | 125 | 79.6 | 1462 | 73.9 |  |
| Missing^§^ | 1 | - | 30 | - |  |
| **Pregnant** |  |  |  |  |  |
| No | 148 | 97.4 | 1934 | 98.4 | 0.515 |
| Yes | 4 | 2.6 | 31 | 1.6 |  |
| Missing | 6 | - | 44 | - |  |

**^†^***Mann Whitney U test*

**^‡^***Chi squared test or Fisher’s exact test when cell counts < 5*

^§^*Missing as vaccinated 1-13 days before onset of symptoms*

**Table S20. Descriptive characteristics of cases with influenza A(H3N2) and controls, primary care influenza vaccine effectiveness study, Ireland 2023/2024**

| **Exposure** | **Case**  **(334/2,346, 14.2%)** | | **Control**  **(2,012/2,346, 85.8%)** | |  |
| --- | --- | --- | --- | --- | --- |
|  | **Median** | **IQR** | **Median** | **IQR** | **p-value^†^** |
| **Age (years)** | 34 | 17-51 | 39 | 19-57 | **0.004** |
| **Symptom onset to swab (days)** | 3 | 2-4 | 3 | 2-5 | **<0.001** |
| **Exposure** | **n** | **%** | **n** | **%** | **p-value^‡^** |
| **Sex** |  |  |  |  |  |
| Female | 189 | 56.6 | 1254 | 62.3 | 0.053 |
| Male | 145 | 43.4 | 758 | 37.7 |  |
| **Age group (years)** |  |  |  |  |  |
| <2 | 7 | 2.1 | 79 | 3.9 | **0.004** |
| 2-17 | 78 | 23.4 | 398 | 19.8 |  |
| 18-64 | 218 | 65.3 | 1218 | 60.5 |  |
| 65+ | 31 | 9.3 | 317 | 15.8 |  |
| **Vaccination status** | |  |  |  |  |
| Unvaccinated | 267 | 80.7 | 1464 | 73.9 | **0.010** |
| Vaccinated | 64 | 19.3 | 518 | 26.1 |  |
| Missing^§^ | 3 | - | 30 | - |  |
| **Chronic condition** | |  |  |  |  |
| No chronic condition | 230 | 74.2 | 1285 | 69.2 | 0.085 |
| Chronic condition | 80 | 25.8 | 573 | 30.8 |  |
| Missing | 24 | - | 154 | - |  |
| **Month of symptom onset** | |  |  |  |  |
| October-December 2023 | 97 | 29 | 749 | 37.2 | **<0.001** |
| January-March 2024 | 219 | 65.6 | 757 | 37.6 |  |
| March-May 2024 | 18 | 5.4 | 506 | 25.1 |  |
| **Time from symptom onset to swab (days)** | | |  |  |  |
| 0 | 3 | 0.9 | 41 | 2 | **<0.001** |
| 1 | 48 | 14.4 | 243 | 12.1 |  |
| 2 | 101 | 30.2 | 430 | 21.4 |  |
| 3 | 79 | 23.7 | 439 | 21.8 |  |
| 4 | 50 | 15 | 317 | 15.8 |  |
| 5 | 22 | 6.6 | 178 | 8.8 |  |
| 6 | 13 | 3.9 | 120 | 6 |  |
| 7 | 18 | 5.4 | 244 | 12.1 |  |
| **Influenza vaccine type** | |  |  |  |  |
| Live attenuated influenza vaccine | 6 | 1.8 | 65 | 3.3 | 0.058 |
| Other (not specified) | 0 | 0 | 1 | 0 |  |
| Quadrivalent influenza vaccine | 58 | 17.5 | 452 | 22.8 |  |
| Unvaccinated | 267 | 80.7 | 1464 | 73.9 |  |
| Missing^§^ | 3 | - | 30 | - |  |
| **Pregnant** |  |  |  |  |  |
| No | 320 | 99.1 | 1937 | 98.4 | 0.521 |
| Yes | 3 | 0.9 | 31 | 1.6 |  |
| Missing | 11 | 0 | 44 | 0 |  |

**^†^***Mann Whitney U test*

**^‡^***Chi squared test or Fisher’s exact test when cell counts < 5*

^§^*Missing as vaccinated 1-13 days before onset of symptoms*

**Table S21. Descriptive characteristics of cases with influenza B and controls, primary care influenza vaccine effectiveness study, Ireland 2023/2024**

| **Exposure** | **Case**  **(98/2,113, 4.6%)** | | **Control**  **(2,015/2,113, 95.4%)** | |  |
| --- | --- | --- | --- | --- | --- |
|  | **Median** | **IQR** | **Median** | **IQR** | **p-value^†^** |
| **Age (years)** | 26 | 18-37 | 39 | 19-57 | **<0.001** |
| **Symptom onset to swab (days)** | 3 | 2-4 | 3 | 2-5 | 0.054 |
| **Exposure** | **n** | **%** | **n** | **%** | **p-value^‡^** |
| **Sex** |  |  |  |  |  |
| Female | 47 | 48.5 | 1256 | 62.3 | **0.008** |
| Male | 50 | 51.5 | 759 | 37.7 |  |
| Missing | 1 | - | 0 | - |  |
| **Age group (years)** | |  |  |  |  |
| <2 | 0 | 0 | 79 | 3.9 | **<0.001** |
| 2-17 | 23 | 23.5 | 398 | 19.8 |  |
| 18-64 | 75 | 76.5 | 1221 | 60.6 |  |
| 65+ | 0 | 0 | 317 | 15.7 |  |
| **Vaccination status** | |  |  |  |  |
| Unvaccinated | 96 | 98 | 1466 | 73.9 | **<0.001** |
| Vaccinated | 2 | 2 | 519 | 26.1 |  |
| Missing^§^ | 0 | 0 | 30 | 0 |  |
| **Chronic condition** | |  |  |  |  |
| No chronic condition | 70 | 81.4 | 1288 | 69.2 | **0.022** |
| Chronic condition | 16 | 18.6 | 573 | 30.8 |  |
| Missing | 12 | - | 154 | - |  |
| **Month of symptom onset** | |  |  |  |  |
| October-December 2023 | 10 | 10.2 | 749 | 37.2 | **<0.001** |
| January-February 2024 | 49 | 50 | 758 | 37.6 |  |
| March-May 2024 | 39 | 39.8 | 508 | 25.2 |  |
| **Time from symptom onset to swab (days)** | | |  |  |  |
| 0 | 4 | 4.1 | 41 | 2 | **0.026** |
| 1 | 8 | 8.2 | 243 | 12.1 |  |
| 2 | 33 | 33.7 | 431 | 21.4 |  |
| 3 | 19 | 19.4 | 440 | 21.8 |  |
| 4 | 15 | 15.3 | 318 | 15.8 |  |
| 5 | 7 | 7.1 | 178 | 8.8 |  |
| 6 | 8 | 8.2 | 120 | 6 |  |
| 7 | 4 | 4.1 | 244 | 12.1 |  |
| **Influenza vaccine type** | |  |  |  |  |
| Live attenuated influenza vaccine | 0 | 0 | 65 | 3.3 | **<0.001** |
| Other | 0 | 0 | 1 | 0 |  |
| Quadrivalent influenza vaccine | 2 | 2 | 453 | 22.8 |  |
| Unvaccinated | 96 | 98 | 1466 | 73.9 |  |
| Missing^§^ | 0 | - | 30 | - |  |
| **Pregnant** |  |  |  |  |  |
| No | 91 | 96.8 | 1940 | 98.4 | 0.430 |
| Yes | 3 | 3.2 | 31 | 1.6 |  |
| Missing | 0 | - | 44 | - |  |

**^†^***Mann Whitney U test*

**^‡^***Chi squared test or Fisher’s exact test when cell counts < 5*

^§^*Missing as vaccinated 1-13 days before onset of symptoms*

**Table S22. Descriptive characteristics of cases and controls age 2-17 years: Influenza A(H3N2), primary care influenza vaccine effectiveness study, Ireland 2023/2024**

|  | **Cases**  **(n = 78/476, 16.4%)** | | **Controls**  **(n = 398/476, 83.6%)** | |  |
| --- | --- | --- | --- | --- | --- |
| **Exposure** | **Median** | **IQR** | **Median** | **IQR** | **p-value^†^** |
| **Age (years)** | 10 | 6-14 | 9 | 5-13 | 0.179 |
| **Time from symptom onset to swab (days)** | 3 | 2-4 | 3 | 2-5 | **0.038** |
| **Exposure** | **n** | **%** | **n** | **%** | **p-value^‡^** |
| Sex |  |  |  |  |  |
| Female | 36 | 46.2 | 199 | 50 | 0.619 |
| Male | 42 | 53.8 | 199 | 50 |  |
| **Vaccination status** | | | | | |
| Unvaccinated | 72 | 92.3 | 342 | 85.9 | 0.144 |
| Vaccinated | 6 | 7.7 | 56 | 14.1 |  |
| **Chronic condition** | | | | | |
| No chronic condition | 65 | 84.4 | 321 | 84.9 | 0.863 |
| Chronic condition | 12 | 15.6 | 57 | 15.1 |  |
| Missing | 0 | - | 20 | - |  |
| **Month of symptom onset** | | | | | |
| October-December 2023 | 24 | 30.8 | 156 | 39.2 | **<0.001** |
| January-February 2024 | 53 | 67.9 | 143 | 35.9 |  |
| March-May 2024 | 1 | 1.3 | 99 | 24.9 |  |
| **Time from symptom onset to swab (days)** | | | | | |
| 0 | 1 | 1.3 | 12 | 3 | 0.094 |
| 1 | 12 | 15.4 | 43 | 10.8 |  |
| 2 | 27 | 34.6 | 98 | 24.6 |  |
| 3 | 17 | 21.8 | 96 | 24.1 |  |
| 4 | 9 | 11.5 | 61 | 15.3 |  |
| 5 | 8 | 10.3 | 29 | 7.3 |  |
| 6 | 0 | 0 | 23 | 5.8 |  |
| 7 | 4 | 5.1 | 36 | 9 |  |
| **Influenza vaccine type** | | | | | |
| Live Attenuated Influenza Vaccine | 5 | 6.4 | 52 | 13.1 | 0.292 |
| Other (not specified) | 0 | 0 | 1 | 0.3 |  |
| Quadrivalent Influenza Vaccine | 1 | 1.3 | 3 | 0.8 |  |
| Unvaccinated | 72 | 92.3 | 342 | 85.9 |  |

**^†^***Mann Whitney U test*

**^‡^***Chi squared test or Fisher’s exact test when cell counts < 5*

**Table S23. Descriptive characteristics of cases and controls aged 18-64 years: Influenza A(H3N2), primary care influenza vaccine effectiveness study, Ireland 2023/2024**

|  | **Cases**  **(n = 218/1,436, 15.2%)** | | **Controls**  **(n = 1,218/1,436, 84.8%)** | |  |
| --- | --- | --- | --- | --- | --- |
| **Exposure** | **Median** | **IQR** | **Median** | **IQR** | **p-value^†^** |
| **Age (years)** | 40 | 29-51 | 42 | 32-52 | **0.011** |
| **Time from symptom onset to swab (days)** | 3 | 2-4 | 3 | 2-5 | **0.003** |
| **Exposure** | **n** | **%** | **n** | **%** | **p-value^‡^** |
| **Sex** |  |  |  |  |  |
| Female | 134 | 61.5 | 819 | 67.2 | 0.113 |
| Male | 84 | 38.5 | 399 | 32.8 |  |
| **Vaccination status** | |  |  |  |  |
| Unvaccinated | 177 | 81.9 | 943 | 78.8 | 0.317 |
| Vaccinated | 39 | 18.1 | 253 | 21.2 |  |
| Missing^§^ | 0 | 0 | 22 | 0 |  |
| **Chronic condition** | |  |  |  |  |
| No chronic condition | 148 | 75.5 | 788 | 71.1 | 0.228 |
| Chronic condition | 48 | 24.5 | 320 | 28.9 |  |
| Missing | 0 | - | 110 | - |  |
| **Month of symptom onset** | |  |  |  |  |
| October-December 2023 | 61 | 28 | 438 | 36 | **0.001** |
| January-February 2024 | 144 | 66.1 | 479 | 39.3 |  |
| March-May 2024 | 13 | 6 | 301 | 24.7 |  |
| **Time from symptom onset to swab (days)** | | |  |  |  |
| 0 | 1 | 0.5 | 24 | 2 | **0.001** |
| 1 | 32 | 14.7 | 163 | 13.4 |  |
| 2 | 60 | 27.5 | 258 | 21.2 |  |
| 3 | 57 | 26.1 | 244 | 20 |  |
| 4 | 34 | 15.6 | 181 | 14.9 |  |
| 5 | 11 | 5 | 112 | 9.2 |  |
| 6 | 10 | 4.6 | 69 | 5.7 |  |
| 7 | 13 | 6 | 167 | 13.7 |  |
| **Influenza vaccine type** | |  |  |  |  |
| Live Attenuated Influenza Vaccine | 1 | 0.5 | 10 | 0.8 | 0.636 |
| Quadrivalent Influenza Vaccine | 38 | 17.6 | 243 | 20.3 |  |
| Unvaccinated | 177 | 81.9 | 943 | 78.8 |  |
| Missing^§^ | 2 | - | 22 | - |  |

**^†^***Mann Whitney U test*

**^‡^***Chi squared test or Fisher’s exact test when cell counts < 5*

^§^*Missing as vaccinated 1-13 days before onset of symptoms*

**Table S24. Descriptive characteristics of cases and controls aged 65+ years: Influenza A(H3N2), primary care influenza vaccine effectiveness study, Ireland 2023/2024**

|  | **Cases**  **(n = 31/348, 8.9%)** | | **Controls**  **(n = 317/348, 91.1%)** | |  |
| --- | --- | --- | --- | --- | --- |
| **Exposure** | **Median** | **IQR** | **Median** | **IQR** | **p-value^†^** |
| **Age (years)** | 69 | 68-80 | 72 | 69-77 | 0.310 |
| **Time from symptom onset to swab (days)** | 3 | 2-4 | 3 | 2-5 | 0.067 |
| **Exposure** | **n** | **%** | **n** | **%** | **p-value^‡^** |
| Sex |  |  |  |  |  |
| Female | 18 | 58.1 | 201 | 63.4 | 0.694 |
| Male | 13 | 41.9 | 116 | 36.6 |  |
| **Vaccination status** | |  |  |  |  |
| Unvaccinated | 11 | 36.7 | 100 | 32.4 | 0.685 |
| Vaccinated | 19 | 63.3 | 209 | 67.6 |  |
| Missing^§^ | 1 | - | 8 | - |  |
| **Chronic condition** | |  |  |  |  |
| No chronic condition | 10 | 33.3 | 104 | 35.3 | 1.000 |
| Chronic condition | 20 | 66.7 | 191 | 64.7 |  |
| Missing | 0 | - | 22 | - |  |
| **Month of symptom onset** | |  |  |  |  |
| October-December 2023 | 10 | 32.3 | 116 | 36.6 | **0.005** |
| January-February 2024 | 19 | 61.3 | 113 | 35.6 |  |
| March-May 2024 | 2 | 6.5 | 88 | 27.8 |  |
| **Time from symptom onset to swab (days)** | | |  |  |  |
| 0 | 0 | 0 | 3 | 0.9 | 0.186 |
| 1 | 4 | 12.9 | 27 | 8.5 |  |
| 2 | 11 | 35.5 | 57 | 18 |  |
| 3 | 4 | 12.9 | 84 | 26.5 |  |
| 4 | 7 | 22.6 | 58 | 18.3 |  |
| 5 | 2 | 6.5 | 30 | 9.5 |  |
| 6 | 2 | 6.5 | 20 | 6.3 |  |
| 7 | 1 | 3.2 | 38 | 12 |  |
| **Influenza vaccine type** | |  |  |  |  |
| Live Attenuated Influenza Vaccine | 0 | 0 | 3 | 1 | 0.763 |
| Quadrivalent Influenza Vaccine | 19 | 63.3 | 206 | 66.7 |  |
| Unvaccinated | 11 | 36.7 | 100 | 32.4 |  |
| Missing^§^ | 1 | - | 8 | - |  |

**^†^***Mann Whitney U test*

**^‡^***Chi squared test or Fisher’s exact test when cell counts < 5*

^§^*Missing as vaccinated 1-13 days before onset of symptoms*

**Table S25. Descriptive characteristics of cases and controls aged 2-17 years: Influenza A(H1N1)pdm09, primary care influenza vaccine effectiveness study, Ireland 2023/2024**

|  | **Cases**  **(n = 25/422, 5.9%)** | | **Controls**  **(n = 397/422, 94.1%)** | |  |
| --- | --- | --- | --- | --- | --- |
| **Exposure** | **Median** | **IQR** | **Median** | **IQR** | **p-value^†^** |
| **Age (years)** | 8 | 6-14 | 9 | 5-13 | 0.769 |
| **Time from symptom onset to swab (days)** | 3 | 2-4 | 3 | 2-5 | 0.584 |
| **Exposure** | **n** | **%** | **n** | **%** | **p-value^‡^** |
| **Sex** |  |  |  |  |  |
| Female | 16 | 64 | 198 | 49.9 | 0.244 |
| Male | 9 | 36 | 199 | 50.1 |  |
| **Vaccination status** | |  |  |  |  |
| Unvaccinated | 21 | 87.5 | 342 | 86.1 | 1.000 |
| Vaccinated | 3 | 12.5 | 55 | 13.9 |  |
| Missing^§^ | 1 | - | 0 | - |  |
| **Chronic condition** | |  |  |  |  |
| No chronic condition | 22 | 91.7 | 320 | 84.9 | 0.553 |
| Chronic condition | 2 | 8.3 | 57 | 15.1 |  |
| Missing | 0 | - | 20 | - |  |
| **Month of symptom onset** | |  |  |  |  |
| October-December 2023 | 6 | 24 | 156 | 39.3 | **0.006** |
| January-February 2024 | 17 | 68 | 143 | 36 |  |
| March-May 2024 | 2 | 8 | 98 | 24.7 |  |
| **Time from symptom onset to swab (days)** | | |  |  |  |
| 0 | 1 | 4 | 11 | 2.8 | 0.368 |
| 1 | 1 | 4 | 43 | 10.8 |  |
| 2 | 9 | 36 | 98 | 24.7 |  |
| 3 | 6 | 24 | 96 | 24.2 |  |
| 4 | 3 | 12 | 61 | 15.4 |  |
| 5 | 4 | 16 | 29 | 7.3 |  |
| 6 | 1 | 4 | 23 | 5.8 |  |
| 7 | 0 | 0 | 36 | 9.1 |  |
| **Influenza vaccine type** | |  |  |  |  |
| Live Attenuated Intranasal Vaccine | 3 | 12.5 | 51 | 12.8 | 1.000 |
| Other (not specified) | 0 | 0 | 1 | 0.3 |  |
| Quadrivalent Influenza Vaccine | 0 | 0 | 3 | 0.8 |  |
| Unvaccinated | 21 | 87.5 | 342 | 86.1 |  |
| Missing^§^ | 1 | 0 | 0 | 0 |  |

**^†^***Mann Whitney U test*

**^‡^***Chi squared test or Fisher’s exact test when cell counts < 5*

^§^*Missing as vaccinated 1-13 days before onset of symptoms*

**Table S26. Descriptive characteristics of cases and controls aged 18-64 years: Influenza A(H1N1)pdm09, primary care influenza vaccine effectiveness study, Ireland 2023/2024**

|  | **Cases**  **(n = 114/1,331, 8.6%)** | | **Controls**  **(n = 1,217/1,331, 91.4%)** | |  |
| --- | --- | --- | --- | --- | --- |
| **Exposure** | **Median** | **IQR** | **Median** | **IQR** | **p-value^†^** |
| **Age (years)** | 43 | 34-50 | 42 | 32-52 | 0.374 |
| **Time from symptom onset to swab (days)** | 3 | 2-4 | 3 | 2-5 | **0.016** |
| **Exposure** | **n** | **%** | **n** | **%** | **p-value^‡^** |
| **Sex** |  |  |  |  |  |
| Female | 79 | 69.3 | 818 | 67.2 | 0.727 |
| Male | 35 | 30.7 | 399 | 32.8 |  |
| **Vaccination status** | |  |  |  |  |
| Unvaccinated | 96 | 84.2 | 941 | 78.7 | 0.185 |
| Vaccinated | 18 | 15.8 | 254 | 21.3 |  |
| Missing^§^ | 0 | - | 22 | - |  |
| **Chronic condition** | |  |  |  |  |
| No chronic condition | 81 | 79.4 | 787 | 71.1 | 0.084 |
| Chronic condition | 21 | 20.6 | 320 | 28.9 |  |
| Missing | 12 | - | 110 | - |  |
| **Month of symptom onset** | |  |  |  |  |
| October-December 2023 | 30 | 26.3 | 438 | 36 | **<0.001** |
| January-February 2024 | 76 | 66.7 | 479 | 39.4 |  |
| March-May 2024 | 8 | 7 | 300 | 24.7 |  |
| **Time from symptom onset to swab (days)** | | |  |  |  |
| 0 | 1 | 0.9 | 24 | 2 | 0.055 |
| 1 | 15 | 13.2 | 163 | 13.4 |  |
| 2 | 35 | 30.7 | 256 | 21 |  |
| 3 | 28 | 24.6 | 244 | 20 |  |
| 4 | 17 | 14.9 | 182 | 15 |  |
| 5 | 7 | 6.1 | 112 | 9.2 |  |
| 6 | 5 | 4.4 | 69 | 5.7 |  |
| 7 | 6 | 5.3 | 167 | 13.7 |  |
| **Influenza vaccine type** | |  |  |  |  |
| Live Attenuated Influenza Vaccine | 1 | 0.9 | 10 | 0.8 | 0.296 |
| Quadrivalent Influenza Vaccine | 17 | 14.9 | 244 | 20.4 |  |
| Unvaccinated | 96 | 84.2 | 941 | 78.7 |  |
| Missing^§^ | 0 | - | 22 | - |  |

**^†^***Mann Whitney U test*

**^‡^***Chi squared test or Fisher’s exact test when cell counts < 5*

^§^*Missing as vaccinated 1-13 days before onset of symptoms*

**Table S27. Descriptive characteristics of cases and controls aged 65+ years: Influenza A(H1N1)pdm09, primary care influenza vaccine effectiveness study, Ireland 2023/2024**

|  | **Cases**  **(n = 14/330, 4.2%)** | | **Controls**  **(n = 316/330, 95.8%)** | |  |
| --- | --- | --- | --- | --- | --- |
| **Exposure** | **Median** | **IQR** | **Median** | **IQR** | **p-value^†^** |
| **Age (years)** | 72 | 69-76 | 72 | 69-77 | 0.860 |
| **Time from symptom onset to swab (days)** | 3 | 2-4 | 3 | 2-4 | 0.260 |
| **Exposure** | **n** | **%** | **n** | **%** | **p-value^‡^** |
| **Sex** |  |  |  |  |  |
| Female | 7 | 50 | 200 | 63.3 | 0.469 |
| Male | 7 | 50 | 116 | 36.7 |  |
| **Vaccination status** | |  |  |  |  |
| Unvaccinated | 3 | 21.4 | 100 | 32.5 | 0.560 |
| Vaccinated | 11 | 78.6 | 208 | 67.5 |  |
| Missing^§^ | 0 | - | 8 | - |  |
| **Chronic condition** | |  |  |  |  |
| No chronic condition | 7 | 58.3 | 104 | 35.4 | 0.129 |
| Chronic condition | 5 | 41.7 | 190 | 64.6 |  |
| Missing | 2 | - | 22 | - |  |
| **Month of symptom onset** | |  |  |  |  |
| October-December 2023 | 1 | 7.1 | 116 | 36.7 | **0.001** |
| January-February 2024 | 12 | 85.7 | 113 | 35.8 |  |
| March-May 2024 | 1 | 7.1 | 87 | 27.5 |  |
| **Time from symptom onset to swab (days)** | | |  |  |  |
| 0 | 0 | 0 | 3 | 0.9 | 0.874 |
| 1 | 1 | 7.1 | 26 | 8.2 |  |
| 2 | 5 | 35.7 | 57 | 18 |  |
| 3 | 3 | 21.4 | 84 | 26.6 |  |
| 4 | 3 | 21.4 | 58 | 18.4 |  |
| 5 | 1 | 7.1 | 30 | 9.5 |  |
| 6 | 0 | 0 | 20 | 6.3 |  |
| 7 | 1 | 7.1 | 38 | 12 |  |
| **Influenza vaccine type** | |  |  |  |  |
| Live Attenuated Influenza Vaccine | 0 | 0 | 3 | 1.0 | 0.616 |
| Quadrivalent Influenza Vaccine | 11 | 78.6 | 205 | 66.6 |  |
| Unvaccinated | 3 | 21.4 | 100 | 32.5 |  |
| Missing^§^ | 0 | - | 8 | - |  |

**^†^***Mann Whitney U test*

**^‡^***Chi squared test or Fisher’s exact test when cell counts < 5*

^§^*Missing as vaccinated 1-13 days before onset of symptoms*

**Table S28. Descriptive characteristics of cases and controls aged 2-17years: Influenza B, primary care influenza vaccine effectiveness study, Ireland 2023/2024**

|  | **Cases**  **(n = 23/421, 5.5%)** | | **Controls**  **(n = 398/421, 94.5%)** | |  |
| --- | --- | --- | --- | --- | --- |
| **Exposure** | **Median** | **IQR** | **Median** | **IQR** | **p-value^†^** |
| **Age (years)** | 14 | 11-16 | 9 | 5-13 | **0.001** |
| **Time from symptom onset to swab (days)** | 3 | 2-4 | 3 | 2-5 | 0.622 |
| **Exposure** | **n** | **%** | **n** | **%** | **p-value^‡^** |
| **Sex** |  |  |  |  |  |
| Female | 8 | 34.8 | 199 | 50 | 0.228 |
| Male | 15 | 65.2 | 199 | 50 |  |
| **Vaccination status** | |  |  |  |  |
| Unvaccinated | 23 | 100 | 342 | 85.9 | 0.056 |
| Vaccinated | 0 | 0 | 56 | 14.1 |  |
| **Chronic condition** | |  |  |  |  |
| No chronic condition | 18 | 85.7 | 321 | 84.9 | 1.000 |
| Chronic condition | 3 | 14.3 | 57 | 15.1 |  |
| Missing | 2 | - | 20 | - |  |
| **Month of symptom onset** | |  |  |  |  |
| October-December 2023 | 3 | 13 | 156 | 39.2 | **0.019** |
| January-February 2024 | 10 | 43.5 | 143 | 35.9 |  |
| March-May 2024 | 10 | 43.5 | 99 | 24.9 |  |
| **Time from symptom onset to swab (days)** | | |  |  |  |
| 0 | 2 | 8.7 | 12 | 3 | 0.084 |
| 1 | 0 | 0 | 43 | 10.8 |  |
| 2 | 10 | 43.5 | 98 | 24.6 |  |
| 3 | 2 | 8.7 | 96 | 24.1 |  |
| 4 | 4 | 17.4 | 61 | 15.3 |  |
| 5 | 2 | 8.7 | 29 | 7.3 |  |
| 6 | 2 | 8.7 | 23 | 5.8 |  |
| 7 | 1 | 4.3 | 36 | 9 |  |
| **Influenza vaccine type** | |  |  |  |  |
| Live Attenuated Influenza Vaccine | 0 | 0 | 52 | 13.1 | 0.209 |
| Other (not specified) | 0 | 0 | 1 | 0.3 |  |
| Quadrivalent Influenza Vaccine | 0 | 0 | 3 | 0.8 |  |
| Unvaccinated | 23 | 100 | 342 | 85.9 |  |

**^†^***Mann Whitney U test*

**^‡^***Chi squared test or Fisher’s exact test when cell counts < 5*

**Table S29. Descriptive characteristics of cases and controls aged 18-64 years: Influenza B, primary care influenza vaccine effectiveness study, Ireland 2023/2024**

|  | **Cases**  **(n = 75/1,296, 5.8%)** | | **Controls**  **(n = 1,221/1,296, 94.2%)** | |  |
| --- | --- | --- | --- | --- | --- |
| **Exposure** | **Median** | **IQR** | **Median** | **IQR** | **p-value^†^** |
| **Age (years)** | 30 | 23-39 | 42 | 32-52 | **<0.001** |
| **Time from symptom onset to swab (days)** | 3 | 2-4 | 3 | 2-5 | 0.094 |
| **Exposure** | **n** | **%** | **n** | **%** | **p-value^‡^** |
| **Sex** |  |  |  |  |  |
| Female | 39 | 52.7 | 821 | 67.2 | **0.015** |
| Male | 35 | 47.3 | 400 | 32.8 |  |
| Missing | 1 | 0 | 0 | 0 |  |
| **Vaccination status** | |  |  |  |  |
| Unvaccinated | 73 | 97.3 | 945 | 78.8 | **<0.001** |
| Vaccinated | 2 | 2.7 | 254 | 21.2 |  |
| Missing^§^ | 0 | - | 22 | - |  |
| **Chronic condition** | |  |  |  |  |
| No chronic condition | 52 | 80 | 791 | 71.2 | 0.156 |
| Chronic condition | 13 | 20 | 320 | 28.8 |  |
| Missing | 10 | - | 110 | - |  |
| **Month of symptom onset** | |  |  |  |  |
| October-December 2023 | 7 | 9.3 | 438 | 35.9 | **<0.001** |
| January-February 2024 | 39 | 52 | 480 | 39.3 |  |
| March-May 2024 | 29 | 38.7 | 303 | 24.8 |  |
| **Time from symptom onset to swab (days)** | | |  |  |  |
| 0 | 2 | 2.7 | 24 | 2 | 0.125 |
| 1 | 8 | 10.7 | 163 | 13.4 |  |
| 2 | 23 | 30.7 | 259 | 21.2 |  |
| 3 | 17 | 22.7 | 245 | 20.1 |  |
| 4 | 11 | 14.7 | 182 | 14.9 |  |
| 5 | 5 | 6.7 | 112 | 9.2 |  |
| 6 | 6 | 8 | 69 | 5.7 |  |
| 7 | 3 | 4 | 167 | 13.7 |  |
| **Influenza vaccine type** | |  |  |  |  |
| Live Attenuated Influenza Vaccine | 0 | 0 | 10 | 0.8 | **<0.001** |
| Quadrivalent Influenza Vaccine | 2 | 2.7 | 244 | 20.3 |  |
| Unvaccinated | 73 | 97.3 | 945 | 78.8 |  |
| Missing^§^ | 0 | - | 22 | - |  |

**^†^***Mann Whitney U test*

**^‡^***Chi squared test or Fisher’s exact test when cell counts < 5*

^§^*Missing as vaccinated 1-13 days before onset of symptoms*

*There were no cases of influenza B in those aged 65+ years in the 2023/2024 influenza season and therefore no descriptive table is presented.*


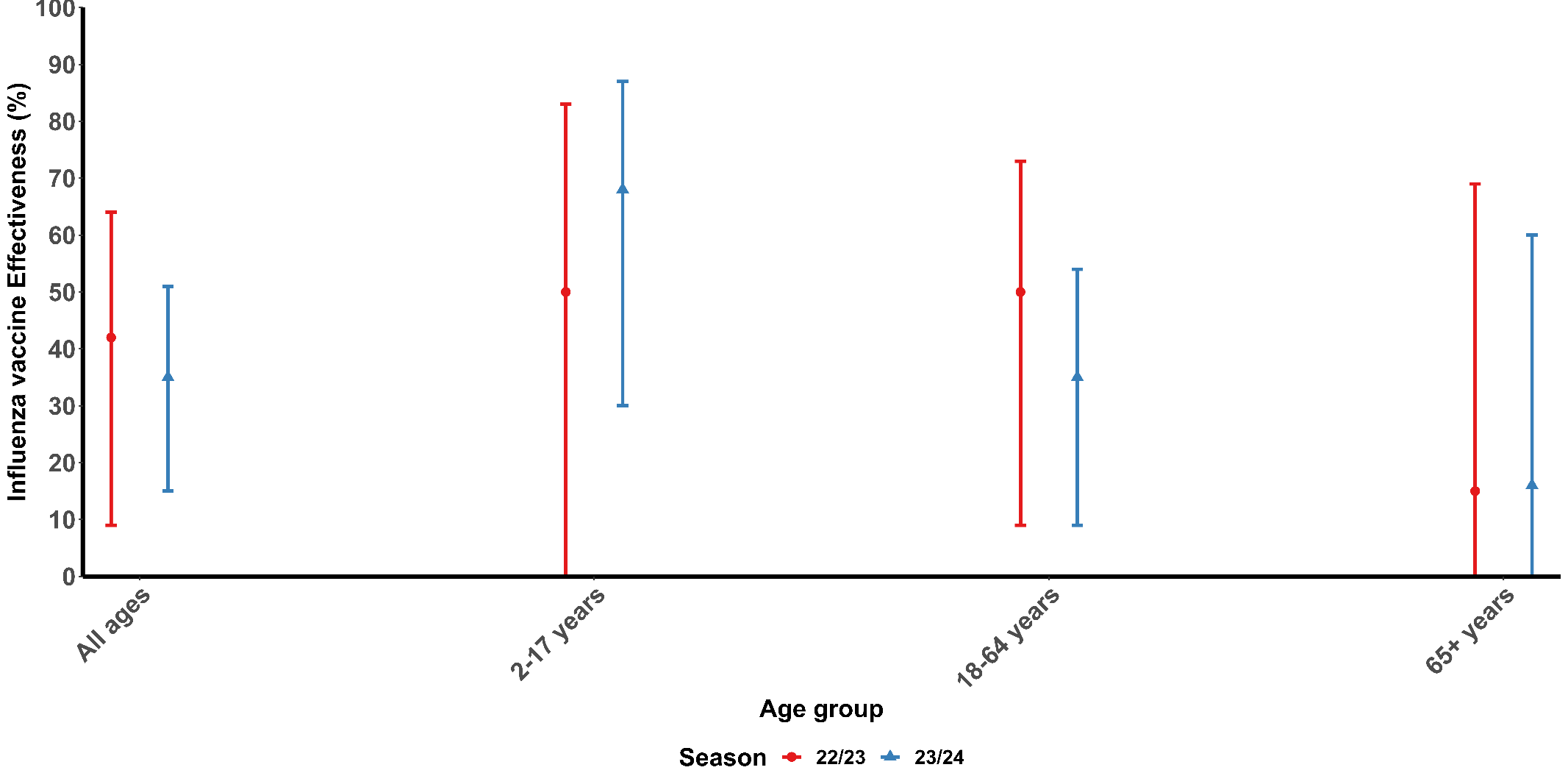
*IVE for the 2-17 year age group in 2022/2023 and IVE for the 65+ age group in both influenza seasons was estimated using Firths penalised logistic regression*

**Figure S13. Influenza vaccine effectiveness (IVE) by age group, primary care IVE study Ireland 2022/2023 and 2023/2024**

*
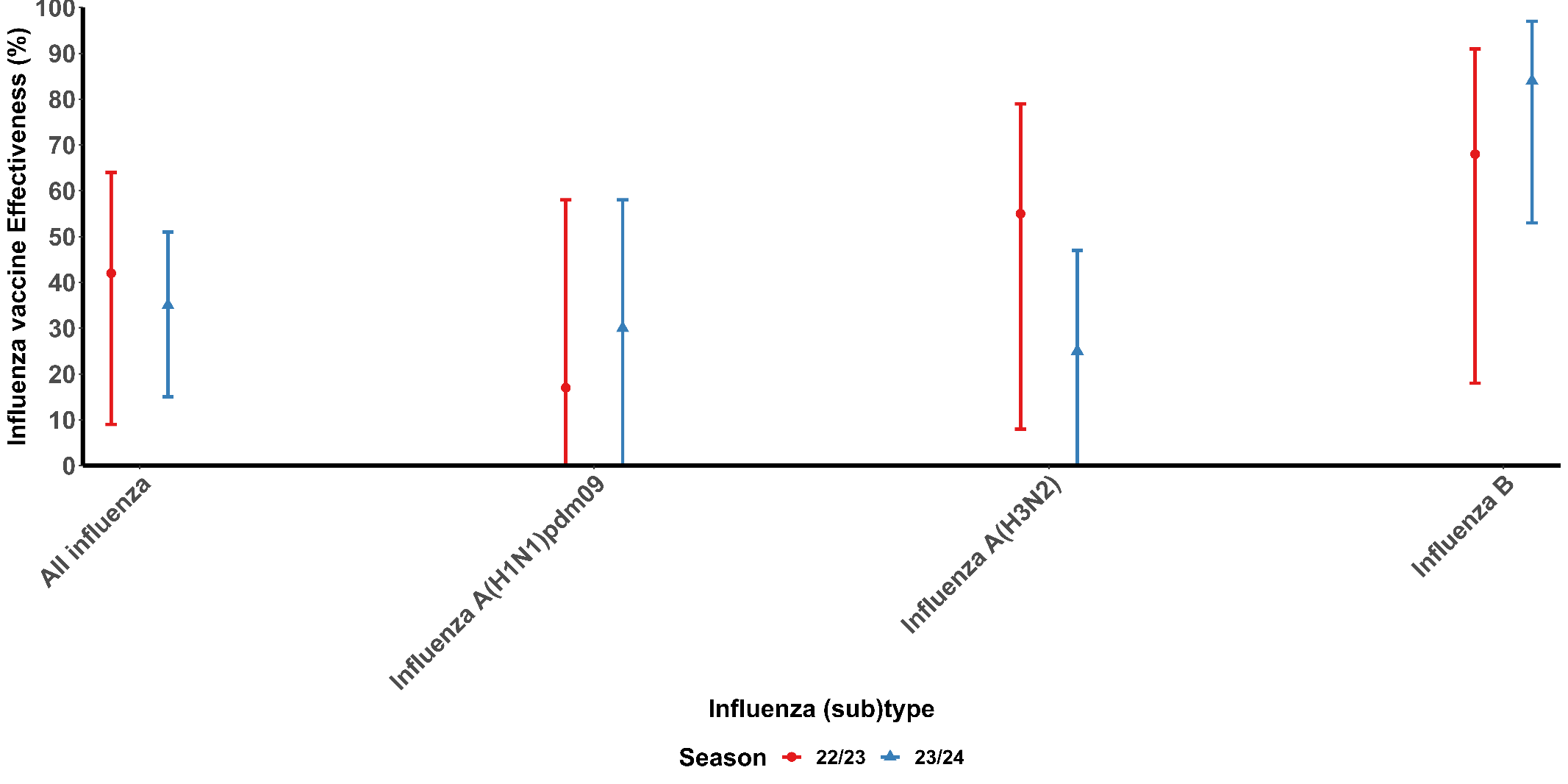
*

*IVE for influenza B in 2023/2024 was estimated using Firths penalised logistic regression*

**Figure S14. Influenza vaccine effectiveness (IVE) by influenza (sub)type, primary care IVE study Ireland 2022/2023 and 2023/2024**

**Table S30 Sensitivity analysis: Overall influenza vaccine effectiveness (IVE) estimates, for all age groups and all (sub)types primary care IVE study, Ireland 2022/2023 and 2023/2024, excluding SARS-CoV-2 positive controls**

| **Season** | **Influenza type** | **Age category** | **Total** | **Cases** | | | **Controls** | | |  |  |  |
| --- | --- | --- | --- | --- | --- | --- | --- | --- | --- | --- | --- | --- |
|  |  |  |  | **Total** | **Vaccinated** | | **Total** | **Vaccinated** | | **IVE^†^** | **CI lower** | **CI Upper** |
|  |  |  | **n** | **n** | **n** | **%** | **n** | **n** | **%** | **%** | **%** | **%** |
| **2022/2023** | All influenza | All ages | 985 | 288 | 42 | 14.6 | 697 | 175 | 25.1 | 42 | 7 | 64 |
| **2023/2024** | All influenza | All ages | 2,268 | 567 | 97 | 17.1 | 1,701 | 458 | 26.9 | 37 | 16 | 53 |

**^†^***Adjusted for age, time of symptom onset, medical condition and sex*

*In 2022/2023 there were 765 controls in the original complete case analysis. In the sensitivity analysis., 68 SARS-COV-2 positive controls were excluded. Therefore 8.9% (68/765) of controls were positive for SARS-CoV-2 in the original complete case analysis.*

*In 2023/2024 there were 1,832 controls in the in original complete case analysis. In the sensitivity analysis. 131 SARS-CoV-2 positive controls were excluded. Overall, 7.2% (131/1,832) of controls were positive for SARS-CoV-2 in the original complete case analysis.*
